# Examining sterically demanding lysine analogs for histone lysine methyltransferase catalysis

**DOI:** 10.1038/s41598-020-60337-3

**Published:** 2020-02-28

**Authors:** Abbas H. K. Al Temimi, Vu Tran, Ruben S. Teeuwen, Arthur J. Altunc, Helene I. V. Amatdjais-Groenen, Paul B. White, Danny C. Lenstra, Giordano Proietti, Yali Wang, Anita Wegert, Richard H. Blaauw, Ping Qian, Wansheng Ren, Hong Guo, Jasmin Mecinović

**Affiliations:** 1 Institute for Molecules and Materials, Radboud University, Heyendaalseweg 135, 6525 AJ Nijmegen, The Netherlands; 2 Department of Physics, Chemistry and Pharmacy, University of Southern Denmark, Campusvej 55, 5230 Odense, Denmark; 3 Department of Blood Transfusion, China-Japan Union Hospital, Jilin University, 126 Xiantai Street, Changchun, 130033 P.R. China; 4 Mercachem B.V., Kerkenbos 1013, 6546 BB Nijmegen, The Netherlands; 5 Chiralix B.V., Kerkenbos 1013, 6546 BB Nijmegen, The Netherlands; 6 Chemistry and Material Science Faculty, Shandong Agricultural University, 271018 Tai’an, Shandong P.R. China; 7 Department of Biochemistry and Cellular and Molecular Biology, University of Tennessee, Knoxville, TN 37996 USA

**Keywords:** Biocatalysis, Transferases, Peptides, Computational chemistry

## Abstract

Methylation of lysine residues in histone proteins is catalyzed by *S*-adenosylmethionine (SAM)-dependent histone lysine methyltransferases (KMTs), a genuinely important class of epigenetic enzymes of biomedical interest. Here we report synthetic, mass spectrometric, NMR spectroscopic and quantum mechanical/molecular mechanical (QM/MM) molecular dynamics studies on KMT-catalyzed methylation of histone peptides that contain lysine and its sterically demanding analogs. Our synergistic experimental and computational work demonstrates that human KMTs have a capacity to catalyze methylation of slightly bulkier lysine analogs, but lack the activity for analogs that possess larger aromatic side chains. Overall, this study provides an important chemical insight into molecular requirements that contribute to efficient KMT catalysis and expands the substrate scope of KMT-catalyzed methylation reactions.

## Introduction

**Figure 1:**
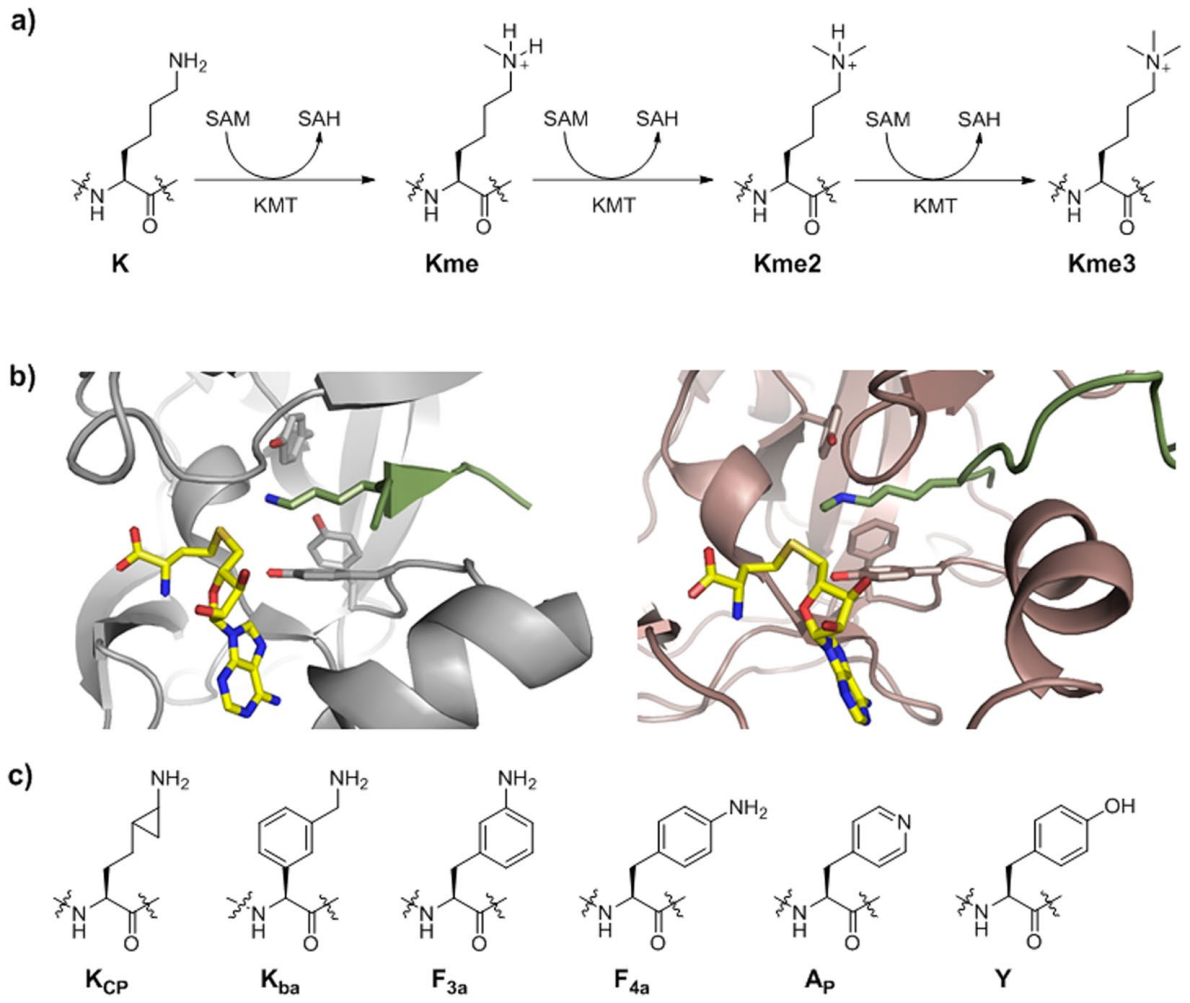
(**a**) Methylation of lysine residues by histone lysine methyltransferase in the presence of SAM cosubstrate. (**b**) View on the crystal structures of SETD8 complexed with H4K20 (green) and SAH (yellow) (left), and GLP complexed with H3K9me (green) peptide and SAH (yellow) (right). (**c**) A panel of sterically demanding lysine analogs.

Posttranslational modifications on histone proteins regulate the structure and function of human chromatin^1–3^. Well-established examples include lysine acetylation, which is linked with the transcriptionally active region of human genome, and lysine methylation, which is associated with gene activation and suppression, depending on the histone sequence and methylation state^4,5^. Histone lysine methylation is catalyzed by *S*-adenosylmethionine (SAM)-dependent histone lysine methyltransferases (KMTs), and can lead to a formation of monomethyllysine (Kme), dimethyllysine (Kme2) and trimethyllysine (Kme3)^6,7^. It is generally believed that the methylation state depends on the constitution of the KMT active site (Fig. 1a)^8^. With the exception of DOT1L, all members of KMT family possess the SET (Su(var)3–9, Enhancer-of-zeste and Trithorax) domain^9–11^. Structural analyses of KMTs complexed with histone peptide/methylated peptide and *S*-adenosylhomocysteine product (SAH) revealed that the lysine side chain occupies a narrow, hydrophobic channel, typically comprised of side chains of several tyrosine and phenylalanine residues (Fig. 1b)^8^. The positioning of the lysine’s N^ε^ amino group towards the electrophilic methyl group of the SAM cosubstrate results in an efficient methyl transfer via S_N_2 reaction^12,13^.

Recent examinations of lysine analogs as substrates for human histone lysine methyltransferases revealed that KMTs possess a high degree of specificity for lysine residues. Enzymatic assays employing MALDI-TOF MS verified that human KMTs preferentially catalyze methylation of lysine residues with L-stereochemistry over D-stereochemistry^14^. Combined experimental and computational studies on histone peptides that bear lysine analogs of different chain length revealed that lysine exhibits an optimal chain length for KMT-catalyzed methylation^15^, and that the enzymatic methylation is limited to N-nucleophiles^16^. Members of KMTs were also found to catalyze methylation of the cysteine-derived γ-thialysine on intact histones and histone peptides^17,18^. Substrate capturing studies using the genetically encoding photo-lysine showed that slightly bulkier γ-diaza-lysine undergoes efficient SETD7-catalyzed methylation in cells^19^. In addition to the essential role of the lysine’s side chain, its main chain also plays an important role in productive KMT catalysis^20^. Despite these recent findings that shed light on basic understanding of KMT catalysis, a broader scope of lysine analogs as substrates for KMTs has not been explored yet. Here we report enzymatic evaluations of sterically demanding lysine analogs as substrates for human KMTs employing MALDI-TOF MS assays, NMR spectroscopic analyses, and quantum mechanical/molecular mechanical (QM/MM) molecular dynamics and free energy studies.

The lysine’s side chain is comprised of four hydrophobic methylene groups and the terminal nucleophilic N^ε^ amino group. The zig-zag orientation of the flexible C-C bonds might enable a proper orientation of the lysine’s side chain in a narrow hydrophobic pocket of KMTs, leading to efficient KMT catalysis. It remains to be established whether this narrow lysine-binding pocket can accommodate larger moieties that resemble lysine. The objective of this work is to explore whether KMTs do have a capacity to catalyze methylation of bulkier lysine analogs present on histone peptides. We selected six sterically demanding lysine analogs: (i) cyclopropyllysine (K_CP_), which bears an additional methylene group adjacent to the N^ε^ amino group; (ii) benzylamine (K_ba_), an analog with a larger but highly nucleophilic side chain; (iii) *meta*-aminophenylalanine (F_3a_), a significantly larger aromatic lysine analog that possesses the terminal N^ε^ amino group with a weaker nucleophilic character; (iv) *para*-aminophenylalanine (F_4a_), another aniline derivative with less nucleophilic N^ε^ amino group; (v) pyridylalanine (A_P_), which possesses a nucleophilic pyridine functionality; and (vi) tyrosine (Y), an electron-rich aromatic system with a potential to undergo O- or C-methylation (Fig. 1c).

## Results and Discussion

**Figure 2:**
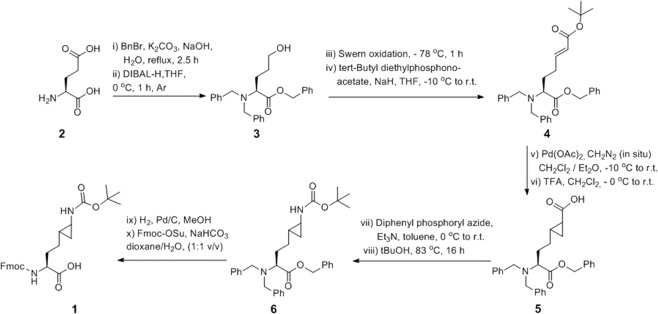
Synthetic strategy for preparation of Fmoc-K_CP_(Boc)-OH **1**.

Fmoc- and Boc-protected cyclopropyllysine (Fmoc-K_CP_(Boc)-OH, **1**) was synthesized in nine steps using a modification of the reported procedure (Fig. 2)^21^. To install an alcohol on the side chain, perbenzylation of L-glutamic acid **2** produced a tetra-substituted compound that underwent selective reduction of the side chain ester in the presence of DIBAL-H to afford the intermediate **3**. Swern oxidation was applied to give the amino aldehyde, which reacted directly with *t*-butyl diethylphosphonoacetate *via* Horner-Wadsworth-Emmons reaction to produce the α,β-unsaturated *t*-butyl ester **4**. ^1^H NMR data confirmed that **4** exists as the *E*-isomer. Diazomethane was then generated *in situ* and distilled directly into a solution of **4** containing catalytic amounts of palladium(II) acetate, to yield the α,β-cyclopropyl *t*-butyl ester, which was selectively hydrolyzed with TFA to yield compound **5**. The ^13^C NMR spectrum of **5** revealed an ~1:1 “doubling” of many of the signals into small doublets. This finding was indicative of either diastereomeric cyclopropylation whereby the methylene is added above or below the alkene plane in roughly equal percentages or that the compound had some form of hindered rotation that resulted in two identical molecules with nearly identical conformations. The latter is unlikely as there are no stereotypical bond-types that form rotamers, and nonspecific cyclopropylation is the more reasonable explanation as two different diastereomers are formed due to the chiral C_α_ of the backbone. The Boc-protected cyclopropyl amine **6** was then produced through a Curtius rearrangement after refluxing in *t*-butanol. Subsequently, deprotection of the benzyl group on amine/carboxylate with Pd/C under hydrogen atmosphere, followed by the Fmoc-protection of the free amine afforded Fmoc-K_CP_(Boc)-OH **1**. The presence of ^13^C “doubling” was also present in the final building block **1**. High-resolution 2D ^1^H-^13^C HSQC-TOCSY spectra were able to produce ^1^H spectra of each of the two diastereomers from the projection of the cross-peaks. These ^1^Hs exhibited very small but noticeable differences in chemical shift (Supplementary Fig. 1). Furthermore, exploring the effect of temperature on the lineshape of the ^1^H signals from 25 °C to 50 °C revealed no significant effects, further supporting the explanation of diastereomers versus rotamers for the observed spectral doubling (Supplementary Fig. 2). The other five Fmoc-protected lysine analogs, *i.e*. Fmoc-K_ba_(Boc)-OH, Fmoc-F_3a_(Boc)-OH), Fmoc-F_4a_(Boc)-OH, Fmoc-A_P_-OH, and Fmoc-Tyr(^t^Bu)-OH, are commercially available. All sterically demanding lysine analogs were incorporated into histone peptides using solid-phase peptide synthesis; H4K20 analogs (GGAKRHR**K**VLRDNIQ), H3K4 analogs (sequence ART**K**QTARKSTGGKA), and H3K9 analogs (sequence ARTKQTAR**K**STGGKA) were synthesized. All histone peptides were purified by preparative HPLC, and the purity of synthetic histone peptides bearing lysine analogs was confirmed by analytical HPLC and ESI-MS analyses (Supplementary Tables 1–2 and Supplementary Figs. 3–13).

**Figure 3:**
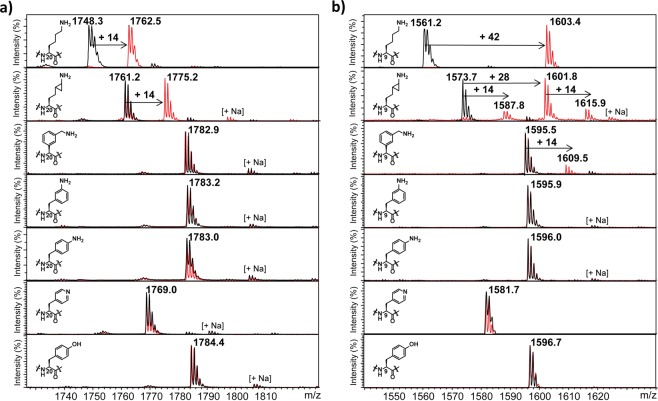
MALDI-TOF MS data showing SAM-dependent methylation of histone peptides in the presence of (**a**) SETD8 and (**b**) GLP. H4K20/H3K9 (first panels), H4K_CP_20/H3K_CP_9 (second panels), H4K_ba_20/H3K_ba_9 (third panels), H4F_3a_20/H3F_3a_9 (fourth panels), H4F_4a_20/H3F_4a_9 (fifth panels), H4A_P_20/H3A_P_9 (sixth panels), and H4Y20/H3Y9 (seventh panels). (Black = control reaction showing the histone peptide in the absence of KMT, red = KMT-catalyzed reaction).

We examined histone peptides bearing lysine and its sterically demanding analogs as potential substrates for human KMTs employing MALDI-TOF MS assays. Enzymatic assays with SETD8 (2 µM) and SAM cosubstrate (200 µM) showed different degrees of methylation of H4K20 peptides (100 µM) after 1 hour at 37 °C. While natural sequence H4K20 underwent quantitative monomethylation, cyclopropyl-containing H4K_CP_20 peptide appeared to be monomethylated to a comparatively lesser extent (60% of H4K_CP_20me) (Fig. 3a). Under the same conditions, none of the other five aromatic lysine analogs were observed to be methylated within limits of detection in the presence of SETD8 (Fig. 3). Increased amounts of SETD8/SAM and prolonged incubation at 37 °C resulted in almost complete formation of the monomethylated H4K_CP_20me product (Supplementary Fig. 14), but still did not lead to appearance of detectable amounts of the monomethylated products of the remaining five lysine analogs (Supplementary Figs. 15–19). As expected, control experiments in the absence of SETD8 or SAM verified that monomethylation of H4K_CP_20 is SETD8-catalyzed and also requires the presence of SAM cosubstrate (Supplementary Figs. 20–21). MALDI-TOF analyses of SETD7-catalyzed methylation of H3K4 peptides showed that none of histone peptides that contain sterically demanding lysine analogs was methylated within limits of detection (only traces of H3K_CP_4 were observed); SETD7 in the presence of SAM indeed catalyzed the formation of monomethylated H3K4 with a natural sequence (Supplementary Fig. 22). At high concentration of SETD7 (10 µM) and SAM (1 mM) and longer incubation (3 hours), an increased amount of the monomethylated H3K_CP_4me product was observed (Supplementary Fig. 23). Despite being monomethyltransferases, SETD7 and SETD8 appear to have somewhat different abilities to accept substrates other than lysine. In line with our work on γ-thialysine^18^, SETD8 seems to have a slightly broader substrate scope than SETD7, possibly due to subtle differences of the active sites (e.g. positioning of Y273 in SETD8 and Y305 in SETD7).

Our recent investigations demonstrated that, in contrast to monomethyltransferases SETD8 and SETD7, H3K9 trimethyltransferases G9a and GLP appear to exhibit a somewhat broader substrate scope for the enzymatic methylation reaction. Enzymatic studies of natural and unnatural H3K9 peptide sequences (100 µM) in the presence of G9a/GLP (2 µM) and SAM (500 µM) at 37 °C showed that both enzymes do have a potential to catalyze methylation of H3K_CP_9, minor methylation of H3K_ba_9 (traces detected), whereas we did not observe any methylated products with other four bulkier lysine analogs within limits of detection (Fig. 3b and Supplementary Fig. 24). H3K_CP_9 underwent predominant GLP-catalyzed dimethylation (75%), while monomethylated (10%) and trimethylated (10%) products were also observed after 1 hour under standard conditions; longer incubation times led to slightly increased amounts of H3K_CP_9me3 (Supplementary Fig. 25). Under the same conditions, H3K_CP_9me2 (60%) and H3K_CP_9me3 (40%) were formed in the presence of G9a after 1 hour, whereas equal amounts of both methylated products were found after 3 hours at 37 °C (Supplementary Fig. 26). Notably, increased amounts of GLP (10 µM) and SAM (1 mM) afforded almost exclusive formation of H3K_CP_9me3 and significant (55%) monomethylation of H3K_ba_9 after 5 hours at 37 °C, whereas other sterically demanding lysine analogs were still not methylated within detection limits (Supplementary Fig. 27). Control experiments in the absence of G9a/GLP or SAM additionally confirmed that both the enzyme and the cosubstrate are required for methylation on H3K_CP_9 to occur (Supplementary Figs. 28–29).

To establish the substrate efficiency of lysine- and K_CP_-containing histone peptides, we carried out enzyme kinetics analysis, employing the MALDI-TOF MS assays^22^. Both enzymes preferentially catalyze methylation of natural histone sequences, however, bulkier K_CP_-containing peptides still underwent favorable kinetics profiles (Table 1 and Supplementary Fig. 30). The lower substrate efficiencies for H4K_CP_20 and H3K_CP_9 compared to natural sequences were a result of higher K_M_ values, implying a less favorable association of bulkier K_CP_ in a narrow binding pocket of KMTs.

**Table 1:** Kinetics parameters for SETD8-catalyzed methylation of H4K20 and H4K_CP_20, and G9a-catalyzed methylation of H3K9 and H3K_CP_9.

	*K*_*M*_ (μM)	*k*_*cat*_ (min^−1^)	*k*_*cat*_/*K*_*M*_ (mM^−1^ min^−1^)
H4K20	103 ± 39	0.54 ± 0.08	5.24
H4K_CP_20	172 ± 37	0.68 ± 0.08	3.96
H3K9	3.45 ± 0.6	20.7 ± 0.03	6012
H3K_CP_9	22.6 ± 5.0	2.78 ± 0.03	124

Next, we carried out competition studies between histone peptides that bear lysine and its analogs. In the presence of SETD8, SAM and equimolar amounts of H4K20 and H4K_CP_20, we observed the formation of both monomethylated products, albeit a comparatively larger degree of monomethylation of H4K20 was found (70% of H4K20me, 40% of H4K_CP_20me). This result implies that H4K20 and H4K_CP_20 do compete for binding with SETD8, and that H4K20 possesses a somewhat higher binding affinity, which presumably leads to being a better substrate for SETD8. It is also possible that subtle differences in sterics and electronics of H4K_CP_20 when compared to H4K20 do contribute to observed differences in the degree of methylation in the competition experiment. In line with observations that sterically demanding lysine analogs do not undergo SETD8-catalyzed methylation, we found that they also do not significantly inhibit monomethylation of H4K20 (Supplementary Fig. 31). These results are in agreement with inhibition and binding studies of related aromatic lysine analogs that exhibited limited ability to associate with SETD8^23^. Similarly, we observed that H3K_CP_9 competes with H3K9 for G9a-catalyzed methylation, however, other bulkier lysine analogs do not significantly inhibit G9a-catalyzed methylation of H3K9 (Supplementary Fig. 32).

**Figure 4:**
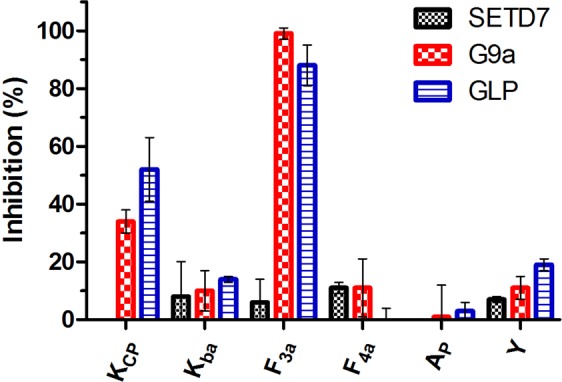
Inhibition of SETD7, G9a and GLP (100 nM) in the presence of 100 µM of H3K*4 (SETD7) or H3K*9 (G9a/GLP) peptides.

We then moved on to investigate in more detail whether the histone peptides bearing unnatural lysine analogs that are not substrates for methyltransferase catalysis, have an ability to inhibit KMT-catalyzed methylation of H3K4 and H3K9. Inhibition studies were carried out employing MALDI-TOF MS assays^24–26^. Initially, all unnatural histone peptides were screened for inhibition at 100 µM (Fig. 4). For H3K4 analogs it was found that all peptides have a very limited ability (IC_50_ > 100 µM) to inhibit SETD7’s methyltransferase activity, at most 11% inhibition was observed at 100 µM of H3F_4a_4. From the peptides bearing unnatural lysine analogs at position 9, we were pleased to find that H3F_3a_9 showed significant inhibition against G9a (IC_50_ = 14.8 µM) and GLP (IC_50_ = 26.0 µM), whereas other histone peptides showed a limited inhibition activity (Fig. 4 and Supplementary Figs. 33–34). For inhibition of GLP by H3K_CP_9, we found that IC_50_ ≈ 100 µM, whereas for the other analogs we observed IC_50_ > 100 µM for both GLP and G9a.

**Figure 5:**
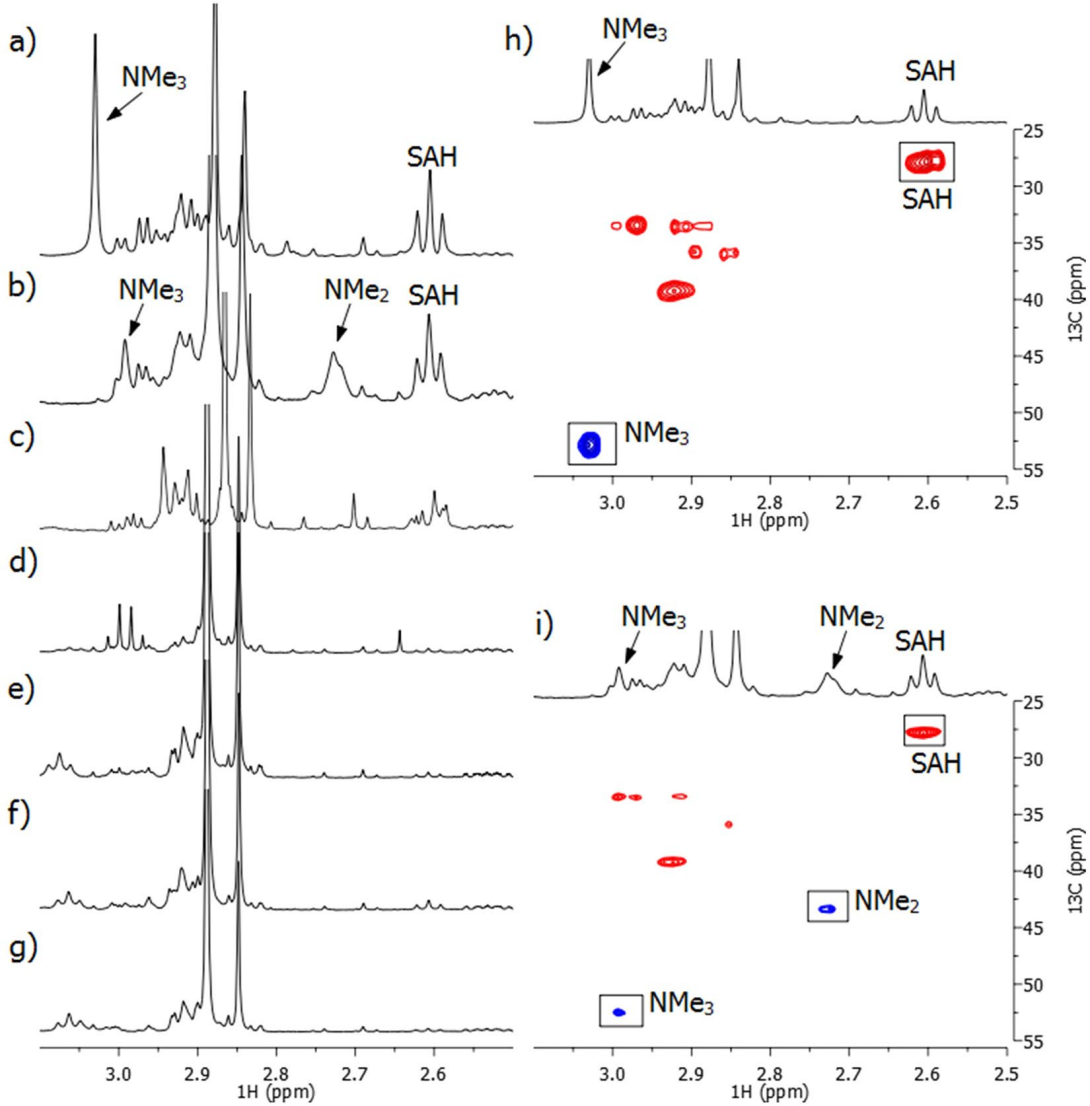
^1^H NMR spectra showing methylations of histone peptides (400 µM) in the presence GLP (8 µM) and SAM (2 mM). (**a**) H3K9; (**b**) H3K_CP_9; (**c**) H3K_ba_9; (**d**) H3F_3a_9; (**e**) H3F_4a_9; (**f**) H3A_P_9; (**g**) H3Y9; (**h**) ^1^H-^13^C HSQC data of H3K9 with the assignment of cross-peaks; (**i**) ^1^H-^13^C HSQC data of H3K_CP_9 with the assignment of cross-peaks.

Having shown that H3K_CP_9 acts as a substrate for GLP, 1D and 2D NMR spectra were acquired to further elucidate the chemical structure of the methylated H3K_CP_9 product (Fig. 5). To characterize the methylated H3K_CP_9 product of GLP-catalyzed reaction, ^1^H NMR and ^1^H-^13^C HSQC (Heteronuclear Single Quantum Coherence) spectra of the H3K_CP_9 peptide were recorded prior to enzymatic reaction (Supplementary Fig. 35). We verified by NMR spectroscopy that GLP-catalyzed methylation of lysine residue in the H3K9 peptide gives indicative signals in the ^1^H NMR spectrum, as also previously examined (Fig. 5a)^15,27^. The appearance of a triplet at 2.62 ppm was assigned to the SAH-CH_2_γ, a characteristic coproduct signal that appears during the methylation reaction of lysine residues by KMTs. In addition, a new resonance at 3.03 ppm indicated the formation of the trimethylated species of lysine residue at position 9. These data were also supported by ^1^H-^13^C HSQC analysis (Fig. 5h). GLP-catalyzed methylation of histone peptides that bear unnatural lysine analogs was also examined by NMR spectroscopy (Fig. 5). As shown in Fig. 5b, ^1^H NMR data of H3K_CP_9 in the presence of SAM and GLP after 1 h at 37 °C showed new resonance peaks of the dimethylated product (H3K_CP_9me2) at 2.73 ppm and the trimethylated product (H3K_CP_9me3) at 2.99 ppm. A triplet of SAH-CH_2_γ was also observed at 2.62 ppm. A conversion of the cyclopropyllysine residue at position 9 to di- and trimethylated products was additionally confirmed by multiplicity-edited HSQC. The resonance at 2.73 ppm in the ^1^H NMR spectrum is in a correlation with (^13^C: 43.1 ppm) and represents the dimethylated product, whereas the resonance at 2.99 ppm is in a correlation with (^13^C: 52.5 ppm) and represents the trimethylated product (Fig. 5i). The methylene protons of the attached cyclopropyl were unable to be observed due to very low concentration, however, chemical shift changes and the addition of new resonances for the cyclopropyl methylene indicate a transformation in the vicinity of the cyclopropyl group. Control reactions with H3K9 and H3K_CP_9 in the absence of GLP showed no formation of methylated products and SAH, again demonstrating that methylation reactions are GLP-catalyzed (Supplementary Figs. 36–37). After showing that the H3K_CP_9 peptide is dimethylated and trimethylated in the presence of GLP and SAM by NMR, we tested whether GLP catalyzed methylation of H3K_ba_9, H3F_3a_9_,_ H3F_4a_9, H3A_P_9 and H3Y9 peptides, and whether GLP mediated the conversion of SAM to SAH. In line with results from MALDI-TOF MS assays, a lack of new characteristic resonances, namely a triplet at 2.62 ppm (SAH-CH_2_γ) and a singlet in the range of 2.5–3.1 ppm (NMe, NMe_2_ or NMe_3_), indicates that these sterically demanding lysine analogs were not methylated in the presence of GLP (Fig. 5c–g and Supplementary Figs. 38–41).

**Figure 6:**
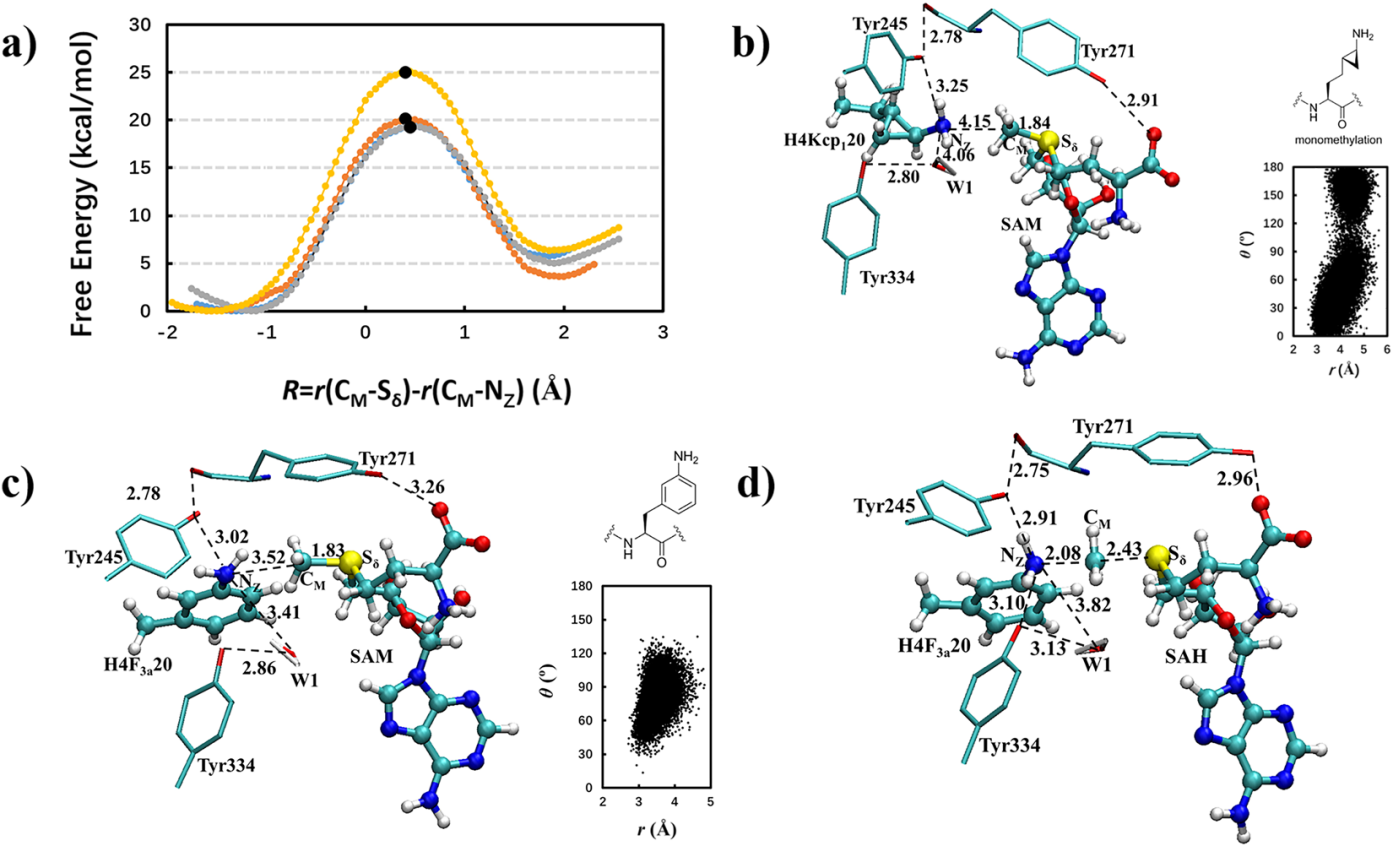
(**a**) Free energy (potential of mean force) profiles for the first methylation reaction in SETD8 involving K, two K_CP_ and F_3a_ as a function of the reaction coordinate [*R* = r(C_M_···S_δ_) – r(C_M_···N_ζ_)]. Blue: H4K20 with a free energy barrier of 19.4 kcal mol^−1^; Orange: K_CP_ with a barrier of 20.0 kcal mol^−1^; Gray: another K_CP_ with a barrier of 19.3 kcal mol^−1^; Yellow: F_3a_ with a barrier of 25.1 kcal mol^−1^. (**b**) Representative active site structure of the reactant complex of SETD8 containing one of the two K_CP_ corresponding the orange line in Fig. 6a (see also the chemical structure inserted). The distribution map on the right shows the alignment of N_ζ_H_2_ and the transferable methyl group in the reactant complex in terms of the distance (*r*) between N_ζ_ and C_M_ and the angle (θ) between the direction of electron lone pair on N_ζ_ and the C_M_-S bond. (**c**) Representative active site structure of the reactant complexes of SETD8 with F_3a_. (**d**) Representative active site structure of the near transition state for the methylation involving F_3a_.

To gain additional insight into KMT-catalyzed methylation of bulkier lysine analogs, we carried out quantum mechanical/molecular mechanical (QM/MM) molecular dynamics and free energy studies on SETD8 and GLP in complex with K_CP_ and F_3a_. The free-energy profiles for the monomethylation reactions in SETD8 involving H4K20, two diastereoisomers of K_CP_ (see the structure inserted in Fig. 6b and Supplementary Fig. 42) and F_3a_ are plotted in Fig. 6a. The free energy barriers for the methyl transfers obtained here are 20.0 and 19.3 kcal mol^−1^ for the two diastereoisomers of K_CP_, respectively, that are quite similar to the barrier when H4K20 was used as the substrate (19.4 kcal mol^−1^). The active site structures of the reactant complexes for the methylations (Fig. 6b and Supplementary Fig. 42) show that the lone pair of electrons on N_ζ_ of K_CP_ can be aligned with the transferable methyl group even with the constrains of the three-membered rings. The free-energy profile for the methylation reaction in SETD8 involving F_3a_ shows that the free energy barrier becomes much higher (25.1 kcal mol^−1^), suggesting that the methylation reaction could not occur with this sterically demanding lysine analog even if this molecule was able to bind to the active site (Fig. 6c,d). The active site structure demonstrates that the transferable methyl group from SAM could not be aligned with the lone pair of electrons on N_ζ_ for the methyl transfer to F_3a_. In fact, the N_ζ_H_2_ group is expected to be a part of the conjugated system containing the benzene ring, and one of the hydrogen atoms on N_ζ_ (rather than the lone pair of electrons) would point to the transferable methyl group. Indeed, the distribution map on the right shows that the angle (θ) between the direction of electron lone pair on N_ζ_ and the C_M_-S bond is between 45 and 120 degrees. In order to have the methylation reaction to occur, the N_ζ_H_2_ group needs to undergo some rotations so that the lone pair of electrons can be aligned with the methyl group. Figure 6d shows that this is the case near the transition state where the N_ζ_H_2_ group has undergone rotations with the lone pair of electrons pointing to the transferable methyl group.

**Figure 7:**
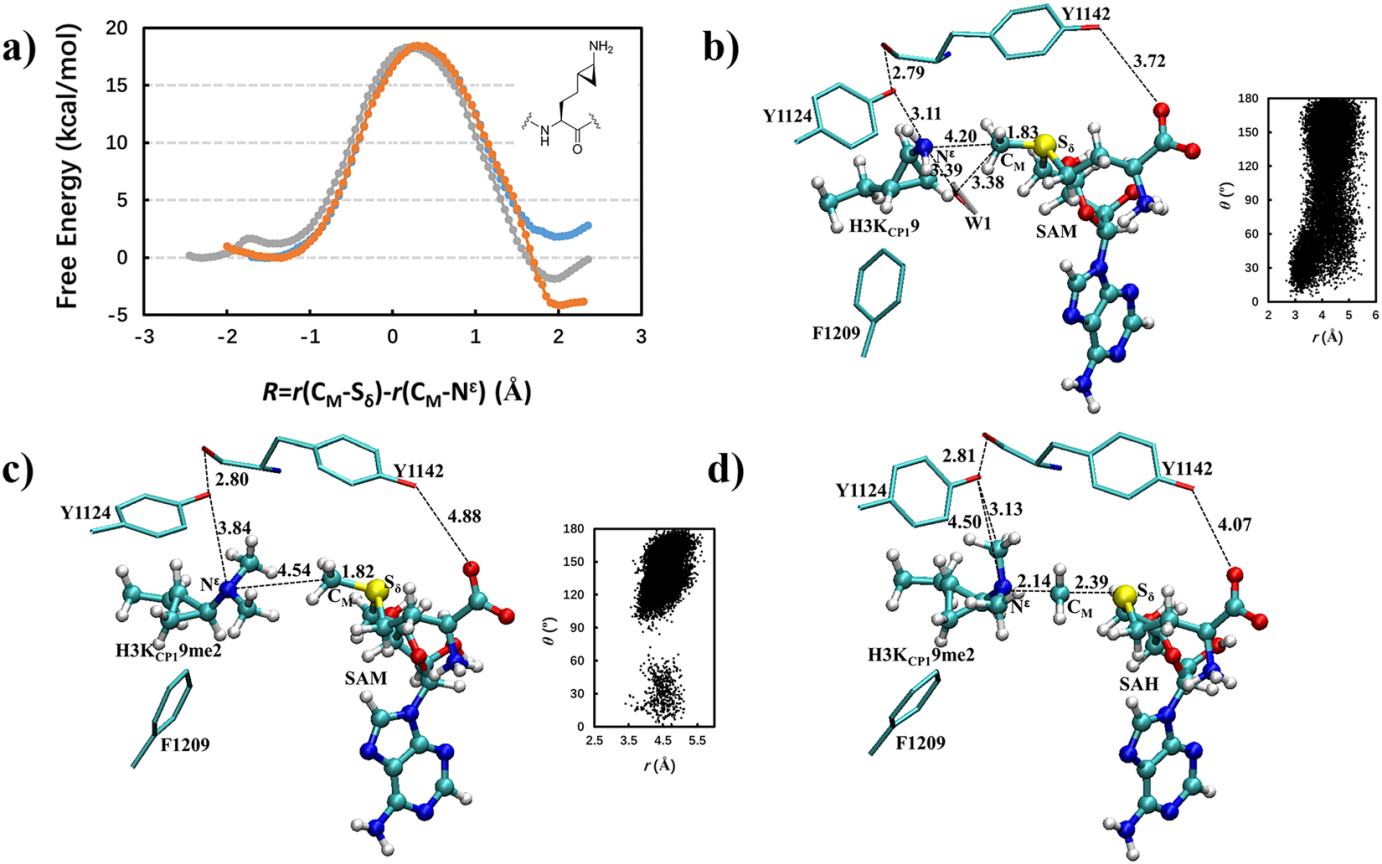
(**a**) Free energy (potential of mean force) profiles for the first, second and third methylation reactions in GLP involving one of the two K_CP_ molecules (see the structure inserted) as a function of the reaction coordinate [*R* = r(C_M_···S_δ_) – r(C_M_···N_ζ_)]; the results for the other K_CP_ are giving in the Supporitng Information. Blue: the first methyl transfer with a free energy barrier of 18.3 kcal mol^−1^; Orange: the second methyl transfer with a barrier of 18.5 kcal mol^−1^; Gray: the third methyl transfer with a barrier of 18.4 kcal mol^−1^. (**b**) Representative active site structure of the reactant complex of GLP containing K_CP_ for the first methyl transfer. (**c**) Representative active site structure of the reactant complex of GLP for the third methyl transfer. (**d**) Representative active site structure of the near transition state for the third methyl transfer.

The free energy profiles for the first, second and third methylation reactions in GLP involving K_CP_ are given in Fig. 7a. As evident from Fig. 7a, all the free energy barriers are rather low and similar (~18–19 kcal mol^−1^), suggesting that GLP is a trimethyltransferase for K_CP_, in agreement with the experiments. Figure 7b shows that for the reactant complex of the first methyl transfer, the transferable methyl group from SAM can be aligned with the lone pair of electrons on N_ζ_. By contrast, for the reactant complex of the third methyl transfer the transferable methyl group from SAM cannot be well aligned with the lone pair of electrons on N_ζ_ (Fig. 7c). Nevertheless, the free energy barrier is rather low as well for the third methyl transfer to K_CP_ (18.4 kcal mol^−1^), indicating that the methylation can still occur. The structure near the transition state for the third methyl transfer is plotted in Fig. 7d (and Supplementary Fig. 43). It is of interest to note that there seems to be some additional transition state stabilization through the interactions involving one of the methyl groups and Y1124. Such interactions may lower the free energy barrier, leading to the third methyl transfer. A similar explanation has been used to understand the substrate/product specificities of Suv4–20h2^28^.

The free energy profile for the first methyl transfer to F_3a_ in GLP shows that the free energy barrier for the methyl transfer is quite high (23.6 kcal mol^−1^), suggesting that GLP cannot catalyze the methylation reaction for F_3a_, as already verified experimentally (Supplementary Fig. 44). Similar to the case involving SETD8, the active site structure shows that the transferable methyl group from SAM cannot be aligned with the lone pair of electrons on N_ζ_ in GLP for the methyl transfer to F_3a_ (Supplementary Fig. 45).

## Conclusion

Overall, our combined synthetic, enzymatic and computational studies, which examine histone peptides that contain sterically demanding lysine analogs, reveal that human histone lysine methyltransferases exhibit a limited ability to catalyze methylation of bulky lysine analogs. Although members of human KMTs do have an ability to catalyze methylation of cyclopropyl-containing lysine (K_CP_) and to a lesser extent benzylamine-containing glycine (K_ba_), they cannot methylate significantly bulkier and less nucleophilic aminophenylalanine, pyridine and tyrosine residues. Despite the biomedical importance of members of KMT family of enzymes, basic molecular requirements for efficient KMT catalysis are only partially understood. Our work provides an important insight into chemical aspects of KMT catalysis by highlighting that human KMTs can accommodate and catalyze methylation of lysine analogs that possess a slightly larger side chain (e.g. K_CP_). Furthermore, we showed that the H3F_3a_9 peptide has an ability to inhibit G9a and GLP methyltransferase activity. This peptide may serve as a starting point for the development of more potent peptide-based inhibitors of G9a and GLP. Along with recent work that has demonstrated that KMTs accept chemically diverse SAM analogs as cosubstrates^29–31^, our study shows that KMTs also possess an ability to catalyze methylation of substrates that mimic lysine. It is envisioned that similar approaches that rely on modern experimental and computational tools will advance our fundamental understanding of epigenetic processes that play essential roles in human health and disease.

## Methods

### Expression and purification of KMTs

Proteins expression and purification were performed as described^15^. Briefly, the four human proteins (SETD8, SETD7, G9a and GLP) were expressed in *E. coli* BL21 (DE3)pLysS-rosetta cells in TB growth medium supplemented with Kanamycin and chloramphenicol. Cells were grown at 37 °C until an OD_600_ of 0.5–0.6. The temperature was then reduced to 16 °C and isopropyl β-D-1-thiogalacttopyranoside (IPTG) was added. Cells were then harvested and lysed by sonication. Purification of the N-terminally his6-tagged KMTs was carried out using Ni-NTA affinity chromatography. Further purification was carried out using size-exclusion chromatography (SEC) using a Superdex-75 preparative grade column on an AKTA system. Protein was separated by SDS-PAGE on a 4–15% gradient polyacrylamide gel (Bio-Rad) and the concentrations were determined using the Nanodrop DeNovix DS-11 spectrophotometer.

### Histone peptides synthesis

The peptides, carboxylated at their C termini for SETD8, G9a and GLP, were synthesized manually using a cartridge (6 mL, 20 µm, Screening Devices B.V., The Netherlands). Amino acids residues protected with acid labile moieties employing fluorenylmethyloxycarbonyl (Fmoc) chemistry. Deprotected peptide H4K20 and its unnatural bulkier lysine derivatives for SETD8 substrate examination were prepared possessing the residues (GGAKRHRK^20^VLRDNIQ). Deprotected peptide H3K4 and its unnatural bulkier lysine derivatives for SETD7 substrate examination were prepared possessing the residues (ARTK^4^QTARKSTGGKA). Deprotected peptide H3K9 and its lysine analogs for G9a and GLP were prepared bearing the residues (ARTKQTARK^9^STGGKA). From a loading batch 0.5 mmol/g, a capacity of 0.21 mmol (100 mg) per each synthesis was employed to obtain the required sequence. All standard amino acids (3.0 equivalents) were coupled using HOBt (3.6 equivalents) and DIPCDI (3.3 equivalents) in dimethylformamide (DMF) for 1 h at room temperature. In case of cyclopropylamine peptide substrate, (1.5 equivalents) of the protected unnatural amino acid was used for the coupling. Fmoc deprotection was performed using 20% piperidine in DMF for 30 min. Modified amino acid residues at position 20 of H4 and positions 9 and 4 of H3 coupled with elongated time overnight to ensure efficient coupling. The Fmoc deprotection and the coupling of the residues were monitored using Kaiser test on few resin-beads. Coupling of the amino acids and Fmoc-deprotection were performed by rolling on a rotating-mixer RM-5 (CAT Zipperer, Staufen, Germany). After the final Fmoc removal, peptides were cleaved from the resin using a 2.5% triisopropylsilane (TIS) and 2.5% water in 95% trifluoroacetic acid (TFA). The peptides were precipitated in cold diethyl ether (−20 °C) and purified via preparative HPLC. The yields of SPPS were estimated as isolated yields, in which the molecular weights of individual peptides were calculated as TFA salts at Lys and Arg positions. The peptides were purified by RP-HPLC on a Phenomenex Gemini-NX C18 column and their purities were assessed using analytical HPLC.

### MALDI-TOF MS assays

MALDI-MS methyltransferases activity experiments were performed using a Bruker instrument in the reflectron positive mode. For regular methyltransferase standard conditions experiment which carried out in 30 µL total volume, the mixture contains peptide (100 µM), SAM (200 µM), SETD8 or SETD7 (2 µM), in assay buffer 50 mM Tris at optimal pH 8.0. In case of G9a and GLP, similar conditions were used, except (500 µM) of SAM was added to the reaction mixture. Samples were incubated in an Eppendorf vial 1.5 mL using thermomixer for 1 h at 37 °C. A 5 µL aliquot of the solution was mixed with 5 µL of MeOH, after which 5 µL of this mixture was mixed with 5 µL of α-cyano-4-hydroxycinamic acid matrix (CHCA, 5 mg/mL in 125:125 µL acetonitrile/water). The spots were placed on a stainless steel MALDI plate (MS 96 target ground steel BC of Bruker, Germany). The mass corresponding to one monomethylation observed as +14 Da, dimethylation observed as +28 Da and trimethylation observed as +42 Da. Data from a set of 100 laser shots (3×) were accumulated to give an acceptable spectrum. The enzymatic activity was determined by taking the peak areas of each methylation state, including all isotopes and adducts, and was annotated using FlexAnalysis software (Bruker Daltonics, Germany). None-enzyme and none-SAM controls experiments were carried out to ensure that the conditions of MS assay did not affect the noticeable methylation states. Methylated peptide substrates were repeated five times and the unmethylated substrates were triplicated. Sequences of the examined peptides are given in (Supplementary Table 1).

### Inhibition studies

A mixture of histone peptide (0–100 µM final conc.) and SETD7, G9a or GLP (100 nM final conc.) was preincubated for 5 minutes at 37 °C in 18 µL of 50 mM glycine pH 8.8 containing 2.5% glycerol as assay buffer. Then 2 µL of a pre-mixture of SAM (20 µM final conc.) and 21-mer H3 histone peptide (residues 1–21, 5 µM final conc.) was added to afford a final assay mixture (20 µL) and the enzymatic reaction was incubated for an additional 30 minutes at 37 °C before quenching with 20 μL of MeOH. The quenched reaction (1 μL) was mixed with a solution of saturated α-cyano-4-hydroxycinnamic acid (5 μL) and spotted on the MALDI plate for crystallisation. The enzymatic activity was determined by taking the peak areas of each methylation state (including all isotopes and adducts) and is expressed relative to a control reaction in the absence of unnatural histone peptides. Each inhibition experiment was carried out in replicate.

### NMR assays

NMR enzymatic experiments for methyl transferase activities were performed with G9a. Incubations by an Eppendorf vials using thermomixer were carried out in 50 mM Tris-*d*_11_.HCl (pD 8.0) and 37 °C for 1 h. The samples (300 µL) typically contained G9a (8 μM) and SAM (2 mM), and H3K9 peptide (400 μM) or any of its sterically demanding analogs H3K_cp_9/H3F_3a_9/H3F_4a_9/H3A_p_9/H3Y9 peptide. After 1 h, the sample diluted to 550 μL and measured by ^1^H NMR at 298 K. Controls were run in parallel at the same time. NMR spectra were acquired using a Bruker Avance III 500 MHz NMR spectrometer equipped with a Prodigy BB cryoprobe. The probe temperature was at 298 K in all instances. The 1D ^1^H spectra were acquired in manual mode, whereas subsequent 2D experiments were acquired in full automation mode. Analysis parameters for ^1^H NMR acquisition were: numbers of scans (NS) 256, relaxation delay 4 seconds, and spectral width (SW) 10 ppm. All the 1D experiments were performed with suppression of residual water signal by presaturation during the relaxation delay using presaturation (pulse program zgpr). Analysis parameters for 2D HSQC acquisition were: NS is 32, relaxation delay 1.5 seconds, acquired size 512, spectral width (SW) for ^1^H was 11 ppm and ^13^C was 160 ppm. When processing HSQC, additional measures such as a *t1* noise reduction produced cleaner spectra. Spectral resolution for HSQC was enhanced by apodization. NMR data were processed using MestreNova software (version 10.0.2). All the spectra were phase and baseline corrected.

### QM/MD methods

To understand the experimental observations, the QM/MM free energy (potential of mean force) and MD simulations were undertaken for SETD8 and GLP to calculate the free energy profiles of the methyl transfers from SAM to some of the unnatural amino acid residues. Three-membered and six-membered rings were introduced into lysine sidechain using the CHARMM program^32^. The QM part of the systems included a portion (–CH_2_–CH_2_–S^+^ (Me) –CH_2_–) of SAM and the lysine analog chains, and the rest of the system was described by MM. To separate the QM and MM parts, the link-atom approach^33^ was applied; a modified TIP3P water model^34^ was used for the solvent. The QM/MM simulations were based on the stochastic boundary molecular dynamics method^35^, which partitions the system into a reaction zone and a reservoir region. The reaction zone was further divided into a reaction region and a buffer region. The radius *r* for reaction region was 20 Å with the buffer region extended over 20 Å ≤ *r* ≤ 22 Å. The N_ζ_ atom of the lysine analogs was used the reference center for partitioning the system. The final systems for the QM/MM simulations had around 5300 atoms (including roughly 900–1000 water molecules). For the QM atoms, the DFTB3 method^35^ was used. This semi-empirical approach has been used on a number of systems previously with reasonable results obtained^36^. The PARAM27 of all-hydrogen CHARMM potential function^37^ was adopted here for the MM atoms.

The reactant complexes of the methylation were generated based on the crystal structures of the enzyme complexes (SETD8: PDB ID = 2BQZ; GLP: PDB ID = 3HNA); SAM was generated by adding a methyl group to SAH. The methyl lysine was changed to lysine by removing the methyl group manually. The two three-membered rings and one six-membered ring were introduced to the lysine sidechain to generate the three lysine analogs with steric constrains. The stochastic boundary systems were first optimized based on the steepest descent (SD) and adopted-basis Newton-Raphson (ABNR) methods and then gradually heated from 50.0 to 298.15 K in 50 ps. The time step used for integration of the equation of motion was 1-fs, and for every 50 fs the coordinates were saved for analyses. 1.5 ns QM/MM MD simulations were performed for each of the reactant complexes^28,38^.

To determine the changes of the free energy (potential of mean force) as a function of the reaction coordinate for the methyl transfer in SETD8 and GLP, respectively, the umbrella sampling method^39^ along with the Weighted Histogram Analysis Method (WHAM)^40^ was applied. The linear combination of *r*(C_M_-N_ζ_) and *r*(C_M_-S_δ_) [*R* = *r*(C_M_-S_δ_)– *r*(C_M_-N_ζ_)] (See Fig. 6b for the atom designation) was used as the reaction coordinate. Thirty windows were obtained, and for each window 50 ps production runs were performed after 50 ps equilibration. For the PMF simulations, the force constants of the harmonic biasing potentials were 50–400 kcal mol^–1^ Å^–2^.

## Supporting information

Supporting information.

## References

[1] Bannister AJ, Kouzarides T (2011). Regulation of chromatin by histone modifications. Cell Res..

[2] Kouzarides T (2007). Chromatin Modifications and Their Function. Cell.

[3] Strahl BD, Allis CD (2000). The language of covalent histone modifications. Nature.

[4] Black JC, Van Rechem C, Whetstine JR (2012). Histone Lysine Methylation Dynamics: Establishment, Regulation, and Biological Impact. Mol. Cell.

[5] Shahbazian MD, Grunstein M (2007). Functions of Site-Specific Histone Acetylation and Deacetylation. Annu. Rev. Biochem..

[6] Luo M (2018). Chemical and Biochemical Perspectives of Protein Lysine Methylation. Chem. Rev..

[7] Martin C, Zhang Y (2005). The diverse functions of histone lysine methylation. Nat. Rev. Mol. Cell Biol..

[8] Schapira M (2011). Structural Chemistry of Human SET Domain Protein Methyltransferases. Curr. Chem. Genomics.

[9] Del Rizzo PA, Trievel RC (2011). Substrate and product specificities of SET domain methyltransferases. Epigenetics.

[10] Dillon SC, Zhang X, Trievel RC, Cheng X (2005). The SET-domain protein superfamily: protein lysine methyltransferases. Genome Biol..

[11] Qian C, Zhou M-M (2006). SET domain protein lysine methyltransferases: Structure, specificity and catalysis. Cell. Mol. Life Sci..

[12] Linscott JA (2016). Kinetic isotope effects reveal early transition state of protein lysine methyltransferase SET8. Proc. Natl. Acad. Sci. USA.

[13] Poulin MB (2016). Transition state for the NSD2-catalyzed methylation of histone H3 lysine 36. Proc. Natl. Acad. Sci. USA.

[14] Belle R (2017). Investigating d-lysine stereochemistry for epigenetic methylation, demethylation and recognition. Chem. Commun..

[15] Al Temimi AHK (2017). Lysine Possesses the Optimal Chain Length for Histone Lysine Methyltransferase Catalysis. Sci. Rep..

[16] Al Temimi AHK (2019). The nucleophilic amino group of lysine is central for histone lysine methyltransferase catalysis. Commun. Chem..

[17] Simon MD (2007). The Site-Specific Installation of Methyl-Lysine Analogs into Recombinant Histones. Cell.

[18] Al Temimi AHK (2019). γ-Thialysine versus Lysine: An Insight into the Epigenetic Methylation of Histones. Bioconjugate Chem..

[19] Yang T, Li X-M, Bao X, Fung YME, Li XD (2015). Photo-lysine captures proteins that bind lysine post-translational modifications. Nat. Chem. Biol..

[20] Al Temimi AHK (2019). Importance of the Main Chain of Lysine for Histone Lysine Methyltransferase Catalysis. Org. Biomol. Chem..

[21] Culhane JC, Wang D, Yen PM, Cole PA (2010). Comparative Analysis of Small Molecules and Histone Substrate Analogues as LSD1 Lysine Demethylase Inhibitors. J. Am. Chem. Soc..

[22] Guitot K (2014). Label-free measurement of histone lysine methyltransferases activity by matrix-assisted laser desorption/ionization time-of-flight mass spectrometry. Anal. Biochem..

[23] Judge RA (2016). Turning a Substrate Peptide into a Potent Inhibitor for the Histone Methyltransferase SETD8. ACS Med. Chem. Lett..

[24] Guitot K (2017). A direct label-free MALDI-TOF mass spectrometry based assay for the characterization of inhibitors of protein lysine methyltransferases. Anal. Bioanal. Chem..

[25] Lenstra DC, Al Temimi AHK, Mecinović J (2018). Inhibition of histone lysine methyltransferases G9a and GLP by ejection of structural Zn(II). Bioorg. Med. Chem. Lett..

[26] Lenstra DC (2018). Structure–Activity Relationship Studies on (R)-PFI-2 Analogues as Inhibitors of Histone Lysine Methyltransferase SETD7. ChemMedChem.

[27] Theillet F-X (2012). Site-Specific Mapping and Time-Resolved Monitoring of Lysine Methylation by High-Resolution NMR Spectroscopy. J. Am. Chem. Soc..

[28] Qian P, Guo H, Wang L, Guo H (2017). QM/MM Investigation of Substrate and Product Specificities of Suv4-20h2: How Does This Enzyme Generate Dimethylated H4K20 from Monomethylated Substrate?. J. Chem. Theory Comput..

[29] Islam K, Zheng W, Yu H, Deng H, Luo M (2011). Expanding Cofactor Repertoire of Protein Lysine Methyltransferase for Substrate Labeling. ACS Chem. Biol..

[30] Luo M (2012). Current Chemical Biology Approaches to Interrogate Protein Methyltransferases. ACS Chem. Biol..

[31] Zhang J, Zheng YG (2016). SAM/SAH Analogs as Versatile Tools for SAM-Dependent Methyltransferases. ACS Chem. Biol..

[32] Brooks BR (1983). CHARMM: A program for macromolecular energy, minimization, and dynamics calculations. J. Comput. Chem..

[33] Field MJ, Bash PA, Karplus M (1990). A combined quantum mechanical and molecular mechanical potential for molecular dynamics simulations. J. Comput. Chem..

[34] Jorgensen WL, Chandrasekhar J, Madura JD, Impey RW, Klein ML (1983). Comparison of simple potential functions for simulating liquid water. J. Chem. Phys..

[35] Brooks CL, Brünger A, Karplus M (1985). Active site dynamics in protein molecules: A stochastic boundary molecular-dynamics approach. Biopolymers.

[36] Cui Q, Elstner M, Kaxiras E, Frauenheim T, Karplus M (2001). A QM/MM Implementation of the Self-Consistent Charge Density Functional Tight Binding (SCC-DFTB) Method. J. Phys. Chem. B.

[37] MacKerell AD (1998). All-Atom Empirical Potential for Molecular Modeling and Dynamics Studies of Proteins. J. Phys. Chem. B.

[38] Guo H-B, Guo H (2007). Mechanism of histone methylation catalyzed by protein lysine methyltransferase SET7/9 and origin of product specificity. Proc. Natl. Acad. Sci. USA.

[39] Torrie GM, Valleau JP (1974). Monte Carlo free energy estimates using non-Boltzmann sampling: Application to the sub-critical Lennard-Jones fluid. Chem. Phys. Lett..

[40] Kumar S, Rosenberg JM, Bouzida D, Swendsen RH, Kollman PA (1992). THE weighted histogram analysis method for free-energy calculations on biomolecules. I. The method. J. Comput. Chem..

