# Supplementary material for: Examining sterically demanding lysine analogs for histone lysine methyltransferase catalysis
Source: Sci Rep. 2020 Feb 28;10:3671. doi: 10.1038/s41598-020-60337-3 (PMC7048932; doi:10.1038/s41598-020-60337-3)
Supplement: Supplementary file 1 — Supporting information. [file 41598_2020_60337_MOESM1_ESM.pdf]

Supplementary Information

Examining sterically demanding lysine analogs for histone lysine methyltransferase catalysis

Abbas H. K. Al Temimi[1], Vu Tran[1], Ruben S. Teeuwen[1], Arthur J. Altunc[1], Helene I. V. Amatdjais-Groenen[1], Paul B. White[1], Danny C. Lenstra[1], Giordano Proietti[2], Yali Wang[1,3], Anita Wegert[4], Richard H. Blaauw[5], Ping Qian[6], Wansheng Ren[6], Hong Guo*[7] and Jasmin Mecinović*[1,2]

[1] Institute for Molecules and Materials, Radboud University, Heyendaalseweg 135, 6525 AJ, Nijmegen, The Netherlands

[2] Department of Physics, Chemistry and Pharmacy, University of Southern Denmark, Campusvej 55, 5230, Odense, Denmark

[3] Department of Blood Transfusion, China-Japan Union Hospital, Jilin University, 126 Xiantai Street, Changchun 130033, P. R. China

[4] Mercachem B.V., Kerkenbos 1013, 6546 BB, Nijmegen, The Netherlands

[5] Chiralix B.V., Kerkenbos 1013, 6546 BB, Nijmegen, The Netherlands

[6] Chemistry and Material Science Faculty, Shandong Agricultural University, 271018, Tai'an, Shandong, P.R. China

[7] Department of Biochemistry and Cellular and Molecular Biology, University of Tennessee, Knoxville, TN, 37996, USA

Table of Contents

1	Experimental procedures	3
2	Synthetic protocols	4
3	Sequences of histone peptides	12
4	ESI-MS analysis of histone peptides	13
5	Analytical HPLC chromatograms	14
6	MALDI-TOF supporting figures	25
7	NMR supporting figures	43
8	QM/MM studies	47
9	References	51

1. Experimental Procedures

Methods

^{1}H NMR and ^{13}C NMR spectra of the cyclopropylamine intermediates synthesis were acquired using Bruker AV 500 equipped with a Prodigy BB probe. Abbreviations for the peaks: s, singlet; d, doublet; t, triplet; q, quartet; dt, doublet of triplets; dq, doublet of quartets; tt, triplet of triplets and m, multiplet. Coupling constants are reported as *J*-values in Hz. All solution-phase reactions were carried out in dried round-bottomed flasks and stirred using magnetic stirring bars. LS-MS analysis for all the peptides was performed on a Thermo Finnigan LCQ-Fleet ESI-ion trap (Thermofischer, Breda, the Netherlands) equipped with a Phenomenex Gemini-NX C18 column, 50 x 2.0 mm, particle size 3 μM (Phenomenex, Utrecht, The Netherlands). An acetonitrile/water gradient containing 0.1 % formic acid was used for elution (5-100 %, 1-50 min, flow 0.2 mL min^{-1}). An electrospray ionization (ESI) was employed and set in positive mode. The scanning range was *m/z* 50-2000. High resolution masses were recorded with a JEOL AccuTOF CS JMS-T100CS mass spectrometer. The molecular mass of the peptides were also measured by MALDI (Matrix Assisted Laser Desorption/Ionization Time of Flight) mass spectrometry on the Bruker Microflex LRF system (Germany). The scanning range was *m/z* 500-4000. Lyophilization was achieved using an ilShin Freeze Dryer (ilShin, Ede, The Netherlands). The solvents were evaporated in a Büchi Rotavapor R-200 and R-3 under vacuum. The room temperature in the reactions is in the range 20-25 °C. After evaporating the solvents by Rotavapor, the intermediates of cyclopropylamine building block were dried under high vacuum dry pump obtained from EDWARDS (model RV8, Heinenoord, The Netherlands). The high purity water for HPLC, LC-MS, and MALDI-Tof MS was purified by a Milli-Q water Q-POD Element system equipped with a 0.22 μM filter from Merck Millipore. For proteins expression and purification, Ultrapure water system MilliQ Millipore was used.

Materials

All reagents were obtained from commercial suppliers and were used without further purification. Preloaded Wang resin (100-200 mesh) as the solid support and Fmoc-Lys(Boc)-OH were purchased from Novabiochem (Darmstad, Germany). Piperidine and N,N'-Disopropylcarbodiimide (DIPCDI) were obtained from Biosolve chemicals (Valkenswaard, The

Netherlands). Tris-d_{11} solution for NMR analysis, 1-Hydroxybenzotriazole (HOBt), trifluoroacetic acid (TFA), Triisopropylsilane (TIS), SAM, SAH, *N,N'*-diisopropylethylamine (DIPEA), and α-cyano-4-hydroxycinnamic acid were purchased from Sigma Aldrich. Breipohl Resin [Fmoc-4-methoxy-4'-(-carboxypropyloxy)-benzhydrylamine linked to Alanyl-aminomethyl] (200-400 mesh) were purchased from Bachem (Bubendorf). Fmoc-Arg(Pbf)-OH, Fmoc-OSu, and 1-[Bis(dimethylamino)methylene]-1H-1,2,3-triazolo[4,5-b]pyridinium 3-oxide hexafluorophosphate (HATU) were obtained from Fluorochem Ltd. (Derbyshire, UK). Fmoc-Ala.OH.H_2O and Fmoc-Asn(Trt)-OH were purchased from Iris Biotech (Marktredwitz, Germany). Fmoc-Ser(^{t}Bu)-OH, Fmoc-Gly-OH, Fmoc-His(Trt)-OH, Fmoc-4-(Boc-amino)-L-phenylalanine, Fmoc-3-(Boc-amino)-L-phenylalanine and Fmoc-3-(4'-pyridyl)-L-alanine were purchased from Chem-Impex Int'l Inc (Illinois, USA). Fmoc-Thr(^{t}Bu)-OH, Fmoc-Gln(Trt)-OH, were obtained from Carbosynth (Berkshire, UK). Dimethylformamide (DMF) (peptide grade) and acetonitrile (HPLC grade were purchased from Actu-All Chemicals b.v (Oss, The Netherlands).

Synthetic protocols

Dibenzyl-*N*,*N*-dibenzyl-L-glutamate

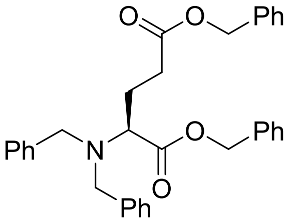

L-glutamic acid **2** (30.0 g, 204.1 mmol) was dissolved in water (400 mL) and the sodium carbonate (86.4 g, 815.1 mmol) and sodium hydroxide (16.34 g, 408.5 mmol) were added. The mixture was refluxed and then benzyl bromide (98 mL, 824.1 mmol, BnBr) was slowly added over 45 min. The reaction mixture turned to a white solution while stirring for 1.5 hours under reflux. Then the reaction mixture was allowed to cool to room temperature and the obtained oil was separated. The water layer was extracted with EtOAc (6 x 200 mL) and the combined organic layers were washed with brine (4 x 100 mL), dried over Na_2SO_4 and evaporated to give the crude product. Column chromatography was used to purify the compound, yielding the pure product as clear viscous oil in 50% (52.2 g, 102.8 mmol). R_f = 0.42 (Heptane: EtOAc 4:1). ^{1}H NMR (400 MHz, $CDCl_3$) δ 7.44-7.14 (m, 20H, Bn-*H*), 5.19 (dd, *J* = 30.8, 12.3 Hz, 2H, Bn-CH_2), 4.96 (q, *J* = 12.4 Hz, 2H, Bn-CH_2), 3.86 (d, *J* = 13.7 Hz, 2H, Bn-CH_2), 3.48 (d, *J* = 13.7 Hz, 2H, Bn-CH_2), 3.39 (t, *J* = 7.7 Hz, 1H, Glu α-*H*), 2.56-2.01 (m,

2H, Glu β-C*H₂*), 2.10-2.02 (m, 2H, Glu γ-C*H₂*). ^{13}C NMR (101 MHz, $CDCl_3$) δ 172.8, 172.2, 139.2, 136.0, 135.9, 128.9, 128.7, 128.6, 128.5, 128.4, 128.3, 128.2, 127.1, 66.2, 66.1, 59.8, 54.5, 30.7, 24.2. IR V_{max}/cm^{-1} 694, 732, 1152, 1453, 1728, 2809. HRMS $[M+H]^+$ calcd. for $C_{33}H_{33}NO_4$, 508.2488; found 508.2501. All data was consistent with that previously reported.[1]

Benzyl-(*S*)-2-(dibenzylamino)-5-hydroxypentanoate 3

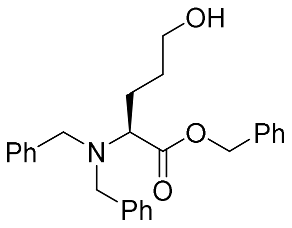

To a solution of dibenzyl-N,N-dibenzyl-L-glutamate (14.30 g, 28.15 mmol) in anhydrous THF (100 mL) under Argon atmosphere at -10 °C was added dropwise 93.5 mL of DIBAL solution (1M in CH_2Cl_2). When addition was finished, the mixture was allowed to warm up to 0 °C and the reaction mixture was stirred for 2 hours. Water (8 mL) was added at 0 °C and the mixture was stirred another 30 min at 0 °C. Additional THF (100 mL) was added and anhydrous Na_2SO_4 and the mixture was stirred another 30 min. The mixture was then filtered over Celite. The solvent was evaporated to give the crude product (16.15 g). Column chromatography was used to purify the product (gradient of 5 to 20% EtOAc in n-pentane), to give the pure product as a clear viscous oil (9.93 g, 87%). R_f = 0.15 (Heptane:EtOAc 4:1). ^{1}H NMR (400 MHz, $CDCl_3$) δ 7.50-7.07 (m, 15H, Bn-*H*), 5.21 (dd, *J* = 30.9, 12.4 Hz, 2H, Bn-C*H₂*), 3.91 (d, *J* = 13.8 Hz, 2H, Bn-C*H₂*), 3.51 (d, *J* = 13.9 Hz, 2H, Bn-C*H₂*), 3.47 (d, *J* = 6.3 Hz, 2H, δ-C*H₂*), 3.37 (t, *J* = 7.5 Hz, 1H, α-*H*), 1.82 (q, *J* = 7.8 Hz, 2H, β-C*H₂*), 1.75-1.42 (m, 2H, γ-C*H₂*). ^{13}C NMR (101 MHz, $CDCl_3$) δ 172.7, 139.4, 136.0, 128.9, 128.6, 128.5, 128.4, 128.2, 127.0, 66.1, 62.4, 60.5, 54.5, 29.3, 25.8. IR V_{max}/cm^{-1} 697, 745, 1132, 1454, 1495, 1727, 2947, 3030, 3392. HRMS $[M+H]^+$ calcd. for $C_{26}H_{29}NO_3$, 404.2226; found 404.2229. All data was consistent with that previously reported.[1]

Benzyl-(*S*)-2-(dibenzylamino)-5-oxopentanoate

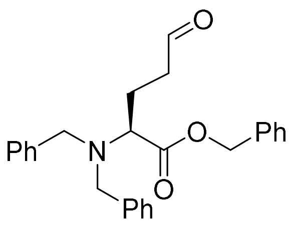

Anhydrous DMSO (2.50 mL) in CH_2Cl_2 (anhydrous, 30 mL) was slowly added to 1.60 mL of oxalylchloride (2.32 g, 18.27 mmol) in CH_2Cl_2 (anhydrous, 60 mL) under Argon atmosphere and cooled to -78 °C. The mixture was stirred for 30 min. at -78 °C. Then a solution of compound **3** (5.80 g, 14.36 mmol) in CH_2Cl_2 (anhydrous, 20 mL) was slowly added. After 15 min 4.7 mL anhydrous Et_3N (33.8 mmol) was added, and the mixture was stirred for 15 min. Additional 4.4 mL anhydrous Et_3N (31.6 mmol) was added, and the mixture allowed to warm up to 0°C and stirred for 15 min. Water (45 mL) was added and the mixture was allowed to warm up to rt. The organic phase was separated, and the water layer was extracted with CH_2Cl_2 (2 x 50 mL). The combined organic layers were washed with 1M HCl (50 mL), sat. $NaHCO_3$ solution (50 mL) and brine (50 mL). The extract was dried over Na_2SO_4 and evaporated to give the product as clear viscous oil (5.45 g, 13.56 mmol, 94%) and this was used without any further purification. R_f = 0.50 (Heptane/EtOAc 4:1). ^{1}H NMR (400 MHz, CDCl3) δ 9.58 (t, *J* = 1.2 Hz, 1H, δ C(O)-*H*), 7.51-7.10 (m, 15H, Bn-*H*), 5.22 (dd, *J* = 27.8, 12.2 Hz, 2H, Bn-C*H2*), 3.87 (d, *J* = 13.7 Hz, 2H, Bn-C*H2*), 3.50 (d, *J* = 13.7 Hz, 2H, Bn-C*H2*), 3.87 (d, *J* = 13.7 Hz, 4H), 3.50 (d, *J* = 13.7 Hz, 4H), 3.35 (dt, *J* = 8.8, 6.6 Hz, 1H, α-*H*), 2.61-2.34 (m, 2H, γ-C*H2*), 2.11-1.94 (m, 2H, β-C*H2*). 13C NMR (101 MHz, CDCl3) δ 201.5, 172.1, 139.1, 135.9, 129.0, 128.9, 128.7, 128.6, 128.5, 128.4, 128.3, 127.2, 66.3, 59.8, 54.5, 40.5, 21.6. IR Vmax/cm^{-1} 695, 732, 1127, 1453, 1724, 2722, 2839, 2940, 3029. HRMS $[M+H]^+$ calcd. for $C_{31}H_{37}N_3O_4$, 402.2069; found 402.2067. All data was consistent with that previously reported.[1]

7-Benzyl 1-(*tert*-butyl) (*S*,*E*)-6-(dibenzylamino)hept-2-enedioate 4

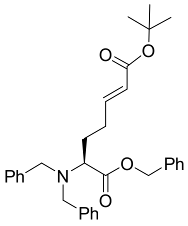

t-Butyl 2-(diethoxyphosphoryl)acetate (4.39 mL, 4.71 g, 18.68 mmol) was added to a suspension of NaH (0.747 g, 18.68 mmol) in THF (anhydrous, 80 mL) under an Argon atmosphere cooled to -5°C. The mixture was allowed to warm up to r.t. and stirred for 30 min, before cooling down to -15°C (dry ice/acetone). A solution of the compound 5 (5.00 g, 12.45 mmol) in THF (anhydrous, 50 mL) was added dropwise and the mixture was stirred for 30 min. The solvent was removed in vacuo and the oil was separated in 50 mL water and diethyl ether (Et_2O, 100 mL).

The water layer was extracted with Et_2O (2 x 50 mL). The combined organic layers were washed with sat. $NaHCO_3$ solution (50 mL) and brine (50 mL). The extract was dried over Na_2SO_4 and evaporated to give the product as a golden oil (3.88 g, 7.77 mmol, 62%). Rf = 0.55 (Heptane/EtOAc 4:1). ^{1}H NMR (400 MHz, $CDCl_3$) δ 7.46-7.33 (m, 5H, Ar-*H*), 7.33-7.17 (m, 10H, Ar-*H*), 6.70 (dt, *J* = 15.6, 6.8 Hz, 1H, C=C-*H*), 5.58 (dt, *J* = 15.6, 6.8 Hz, 1H, C=C-*H*), 5.30-5.10 (m, 2H, Bn-CH_2), 3.69 (dd, *J* = 149.2, 13.8 Hz, 4H, Bn-CH_2), 3.34 (dt, *J* = 8.8, 6.6 Hz, 1H, α-*H*), 2.38-2.00 (m, 2H, γ-CH_2), 1.86 (m, 2H, β-CH_2), 1.46 (s, 9H, 3 x Boc CH_3). ^{13}C NMR (101 MHz, $CDCl_3$) δ 172.4, 165.8, 146.6, 139.3, 136.0, 128.9, 128.6, 128.5, 128.4, 128.3, 127.1, 80.0, 66.1, 60.2, 54.5, 28.6, 28.2, 27.9. IR (Film, v_{max}/cm^{-1}) 3063, 3027, 2938, 2848, 1707, 1654, 1452, 1363, 1148, 974, 747, 691. HRMS $[M+H]^+$ calcd. for $C_{32}H_{37}NO_4$, 500.2801; Observed 500.2805. All data was consistent with that previously reported.[1]

***tert*-Butyl 2-((*S*)-4-(benzyloxy)-3-(dibenzylamino)-4-oxobutyl)cyclopropane-1-carboxylate**

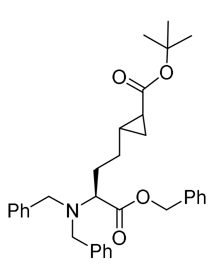

Diazald (3.2 g) was dissolved in 45 mL Et_2O and cooled to 0 °C, then a cooled solution of 0.60 g KOH in 15 mL ethanol (96%) was added and after 5 min the diazomethane in Et_2O was distilled directly on to compound 6 (3.88 g, 7.77 mmol) and palladium (II) acetate (13 mg, 0.058 mmol) that were dissolved in 2:1 anhydrous CH_2Cl_2 (30 mL) and anhydrous Et_2O (15 mL) and cooled down to 0 °C under an Argon atmosphere. The mixture was allowed to warm up and stirred at rt overnight. The reaction mixture was filtered over Celite and concentrated. This was repeated with 1.33 times the amount of diazomethane several times until only 1% of the starting material remained (according to ^{1}H NMR. This yielded the product as a golden oil and this was used without further purification (3.6 g, 7.01 mmol, 90%). ^{1}H NMR (400 MHz, $CDCl_3$) δ 7.44-7.12 (m, 15H, Ar-*H*), 5.20 (d, *J* = 29.3, 2H, Bn-*H*), 3.89 (d, *J* = 13.9 Hz, 2H, Bn-CH_2), 3.50 (d, *J* = 13.9 Hz, 2H, Bn-CH_2) 3.35 (m, 1H, α-*H*), 1.82-1.87 (m, 2H, β-CH_2), 1.42 (d, *J* = 6.0 Hz, 9H, 3 x Boc CH_3), 1.33-1.24 (m, 2H, γ-CH_2), 1.20-1.12 (m, 1H, δ-C*H*), 0.91-0.42 (m, 2H, CH_2). ^{13}C NMR (101 MHz, $CDCl_3$) δ 173.5, 173.4, 172.7, 172.4, 139.5, 136.1, 128.8, 128.7, 128.6, 128.5, 128.4, 128.3, 127.0, 80.0, 66.0, 60.7, 60.6, 54.5, 29.7, 29.7, 29.1, 29.0, 28.2, 28.1, 21.9, 21.9, 21.2, 21.1, 15.3, 15.0. IR (Film, v_{max}/cm^{-1}) 3031, 2931,

2855, 1717, 1455, 1366, 1147, 965, 731, 696. HRMS $[M+H]^+$ calcd. for $C_{33}H_{39}NO_4$, 515.2957; observed 515.2954. Data are in accordance to that previously reported.[1]

2-((*S*)-4-(benzyloxy)-3-(dibenzylamino)-4-oxobutyl)cyclopropane-1-carboxylic acid 5

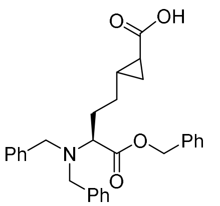

The protected intermediate (3.50 g, 6.81 mmol) was dissolved in CH_2Cl_2 (75 mL) and cooled to 0 °C, then trifluoroacetic acid (75 mL, TFA) is added and the mixture was stirred for 30 min at 0 °C. The mixture was allowed to warm up and stirred at rt for 1.5 hours. The solvent was removed and the oil was separated in 50 mL water and 50 mL CH_2Cl_2. The aqueous phase was extracted with CH_2Cl_2 (2 x 25 mL). The combined organic phases were washed with brine (50 mL), dried over Na_2SO_4 and evaporated. This whole procedure was repeated a second time. This product was used without further purification and the yield of the TFA salt was 3.12 g (5.45 mmol, 80%). ^{1}H NMR (400 MHz, $CDCl_3$) δ 7.44-7.19 (m, 15H, Ar-*H*), 5.20 (m, 2H, Bn-C*H*$_2$), 3.71 (dd, *J* = 149.1, 13.5, 4H, Bn-C*H*$_2$), 3.40-3.33 (m, 1H, α-*H*), 1.94-1.75 (m, 2H, β-*H*$_2$), 1.51-1.07 (m, 2H, γ-C*H*$_2$), 1.20-1.12 (m, 1H, C*H*), 0.92-0.56 (m, 2H, C*H*$_2$). ^{13}C NMR (101 MHz, $CDCl_3$)) δ 180.1, 172.4, 139.1, 135.9, 128.9, 128.6, 128.5, 128.4, 128.3, 127.2, 77.2, 66.2, 60.5, 60.3, 54.6, 29.6, 28.9, 23.3, 20.0, 16.2. IR (KBr, v_{max}/cm^{-1}) 3030, 2930, 2855, 1727, 11690, 1454, 1075, 956, 745, 697. HRMS $[M+H]^+$ calcd. for $C_{29}H_{31}NO_4$, 458.2331; observed 458.2333. All data was consistent with that previously reported.[1]

(2*S*)-4-(2-((*tert*-butoxycarbonyl)amino)cyclopropyl)-2-(dibenzylamino)butanoic acid 6

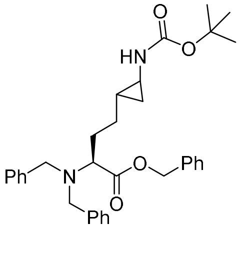

Compound **5** (3.00 g, 5.24 mmol) was dissolved in toluene (anhydrous, 75 mL) and cooled to 0°C under an Argon atmosphere. Then Et_3N (2.8 mL, 2.033 g, 20.09 mmol) and diphenyl phosphorazidate (2.8 mL, 3.58 g, 12.99 mmol) were added and the mixture was allowed to warm up and stirred at rt for 3 hours. The reaction mixture was washed with water (25 mL) and brine (25 mL), dried over Na_2SO_4 and evaporated. The azido product was dissolved in t-butanol (75 mL) and refluxed while stirring under Argon atmosphere overnight. The mixture was cooled down to rt and the solvent was evaporated in vacuo. The oil was separated in 50 mL water and 50 mL CH_2Cl_2. The organic phase was washed with sat. $NaHCO_3$ solution (25

mL) and brine (25 mL), dried over Na_2SO_4 and evaporated. The product was purified with flash chromatography (1.14 g, 2.156 mmol, 41%). Rf = 0.55 (Heptane/EtOAc 4:1). ^{1}H NMR (400 MHz, $CDCl_3$) δ 7.45-7.15 (m, 15H, Bn-*H*), 5.21 (dd, *J* = 27.8, 12.2 Hz, 2H, Bn-C$\underline{H_2}$), 3.89 (d, *J* = 13.8 Hz, 2H, Bn-C$\underline{H_2}$), 3.49 (d, *J* = 13.8 Hz, 2H, Bn-C$\underline{H_2}$), 3.38 (dd, *J* = 6.8, 2.0 Hz, 1H, α-$\underline{H}$), 2.18-2.08 (m, 1H, ε-C$\underline{H}$), 1.93-1.73 (m, 2H, β-C$\underline{H_2}$), 1.43 (d, *J* = 6.2 Hz, 9H, 3 x Boc C$\underline{H_3}$), 1.42-1.09 (m, 2H, γ-C$\underline{H_2}$), 0.58-0.34 (m, 2H, CH_2). ^{13}C NMR (101 MHz, $CDCl_3$) δ 175.1, 156.7, 155.8, 143.9, 143.7, 141.3, 136.1, 127.7, 127.1, 125.2, 5, 120.0, 81.7, 67.3, 53.6, 47.1, 29.7, 28.2, 25.2, 23.7. HRMS $[M+H]^+$ calcd. for $C_{33}H_{40}N_2O_5$, 529.3066, observed 529.3060. All data was consistent with that previously reported.[1]

(2*S*)-2-((((9H-fluoren-9-yl)methoxy)carbonyl)amino)-4-(2-((*tert*-butoxycarbonyl)amino) cyclopropyl) butanoic acid 1

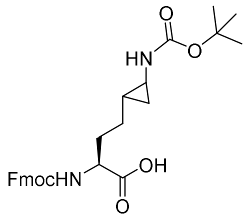

Compound **6** (1.07 g, 2.023 mmol) was dissolved in MeOH (100 mL) and Pd/C was added under a H_2 atmosphere. The mixture was filtrated over Celite and concentrated. The residue was dissolved in 1,4-dioxane (50 mL) and water (50 mL). Then Fmoc-OSu was added and $NaHCO_3$ and the reaction mixture was stirred at rt for 3 hrs. The mixture was concentrated and the extracted with EtOAc (3 x 50 mL) from 5% citric acid solution (50 mL) and was washed with brine (50 mL). The product was purified with flash chromatography (1%-10% MeOH in CH_2Cl_2), to yield an off-white powder (0.65 g, 1.351 mmol, 67%). Rf = 0.25 (10% MeOH in CH_2Cl_2). ^{1}H NMR (400 MHz, $CDCl_3$) δ 7.75 (d, *J* = 7.5 Hz, 2H, Fmoc-$\underline{H}$), 7.63-7.54 (m, 2H, Fmoc-$\underline{H}$), 7.42-7.25 (m, 4H, Fmoc-$\underline{H}$), 4.46-4.29 (m, 2H, Fmoc-C$\underline{H_2}$), 4.28-4.17 (m, 1H, α-$\underline{H}$), 2.45 (s, 1H), 2.28 – 2.14 (m, 1H), 2.14 – 1.99 (m, 1H), 1.78 (t, *J* = 42.1 Hz, 2H), 1.42 (d, *J* = 4.4 Hz, 9H, 3 x Boc C$\underline{H_3}$), 0.66 – 0.42 (m, 2H, C$\underline{H_2}$). ^{13}C NMR (101 MHz, $CDCl_3$) δ 175.1, 157.3, 156.4, 143.9, 143.7, 141.3, 127.7, 127.1, 125.1, 120.0, 80.8, 67.1, 53.4, 47.1, 28.4, 28.1, 20.0, 14.0. IR (Film, v_{max}/cm^{-1}) 3326, 3068, 2979, 2930, 1704, 1511, 1249, 1164, 1078, 909, 759, 732. HRMS $[M+Na]^+$ calcd. for $C_{27}H_{32}N_2O_5$, 503.2158; observed 503.2148. $[\alpha]_D^{25}$ = +3.85 (c = 14.41 g L^{-1}, $CHCl_3$). All data was consistent with that previously reported.[1]

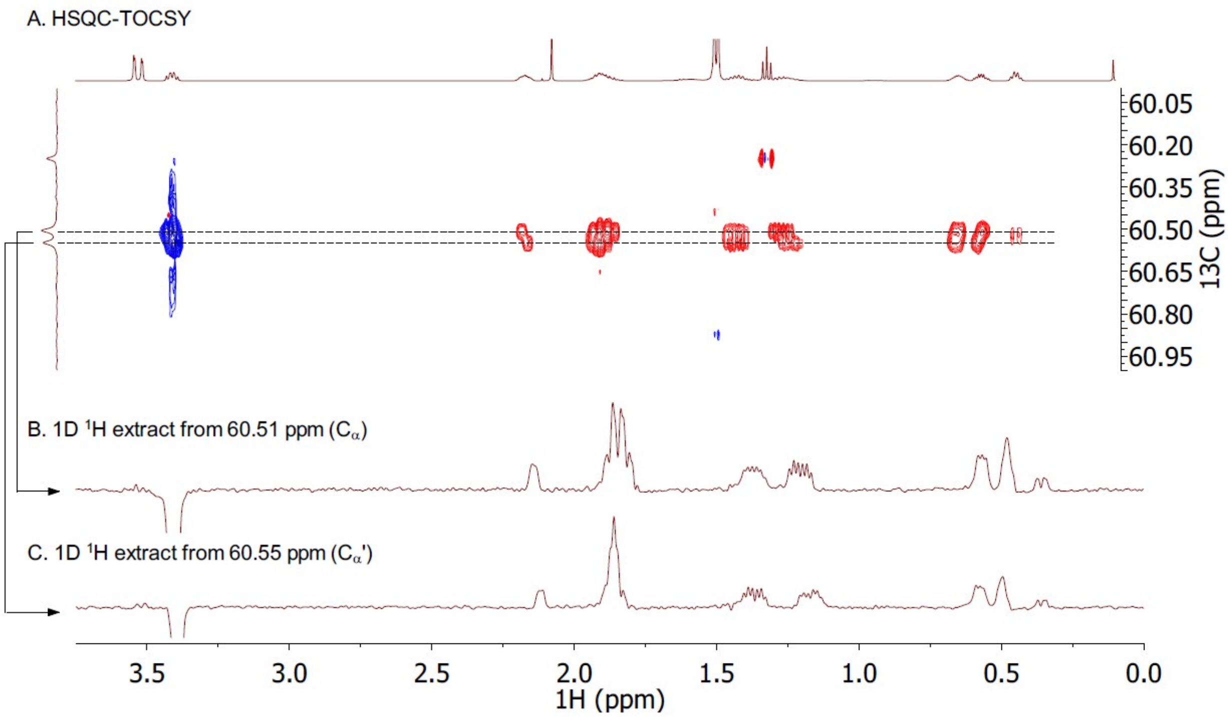

Figure S1. High-resolution HSQC-TOCSY spectra of the amino acid cyclopropyllysine building block. **A)** The original 1-bond correlation vs TOCSY correlations are distinguished by the blue and red coloring, respectively. The spectrum was intentionally and carefully folded in order to observe the minute 5.5 Hz difference between the different C_α ^{13}Cs of each diastereomer without interference from folded cross-peaks. **B)** 1D ^{1}H projection was extracted for C_α at 60.51 ppm to highlight the individual ^{1}H resonances incorporating this diastereomer. **C)** 1D ^{1}H projection was extracted for C_α' at 60.55 ppm to highlight the individual ^{1}H resonances incorporating this diastereomer. ^{1}H resonances incorporating each diastereomer. In many cases, the ^{1}H resonances are similar in shift but some exhibit minor differences between diastereomers.

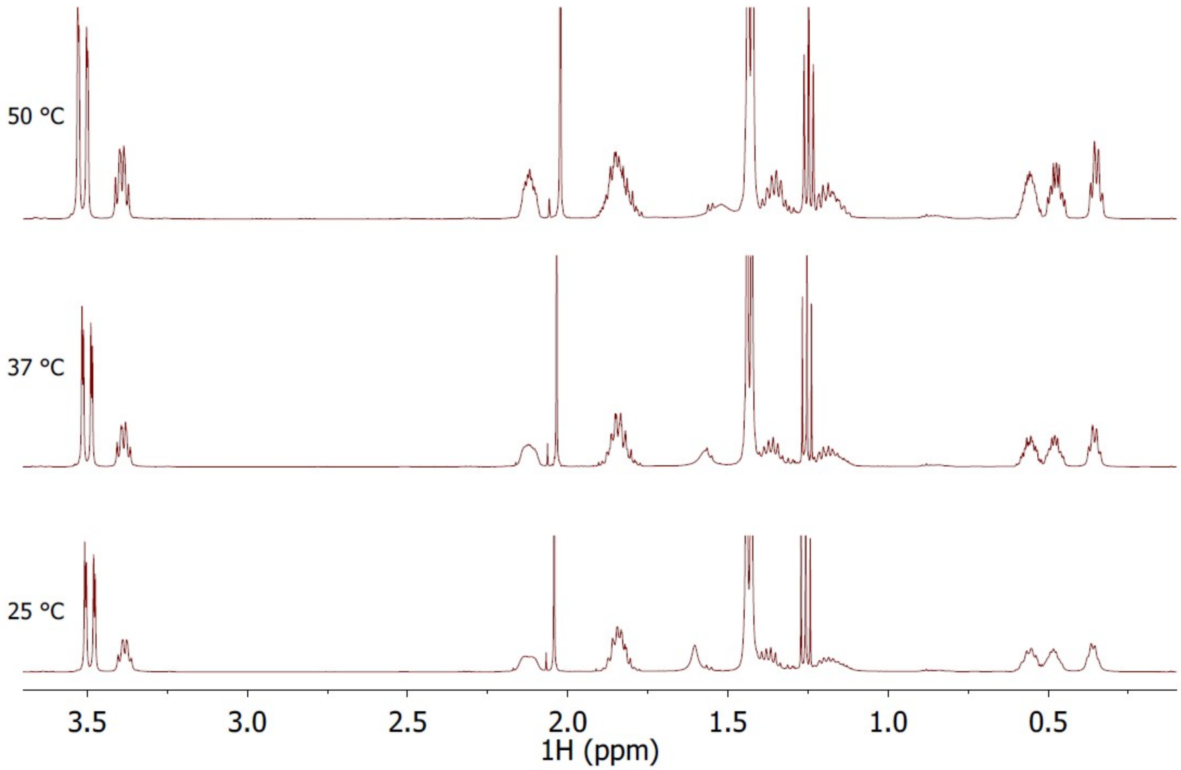

Figure S2. Stacked ^{1}H spectra of variable temperature NMR measurements expanded on the cyclopropyllysine side-chain. Insignificant line broadening or sharpening was observed across the temperature range 25 °C to 50 °C, suggesting that the presence of two sets of peaks observed in the HSQC-TOCSY were not rotamers, but diastereomers. This is additionally supported by the observation of doubling of ^{13}C resonances observed in the carboxylic acid precursor.

3. Sequences of histone peptides. **Table S1.** Sequences of peptides used in this study. Peptides H3X4 were examined as substrates for SETD7. Peptides H4X20 were tested as substrates for SETD8. Peptides H3X9 were examined as substrates for G9a and GLP.

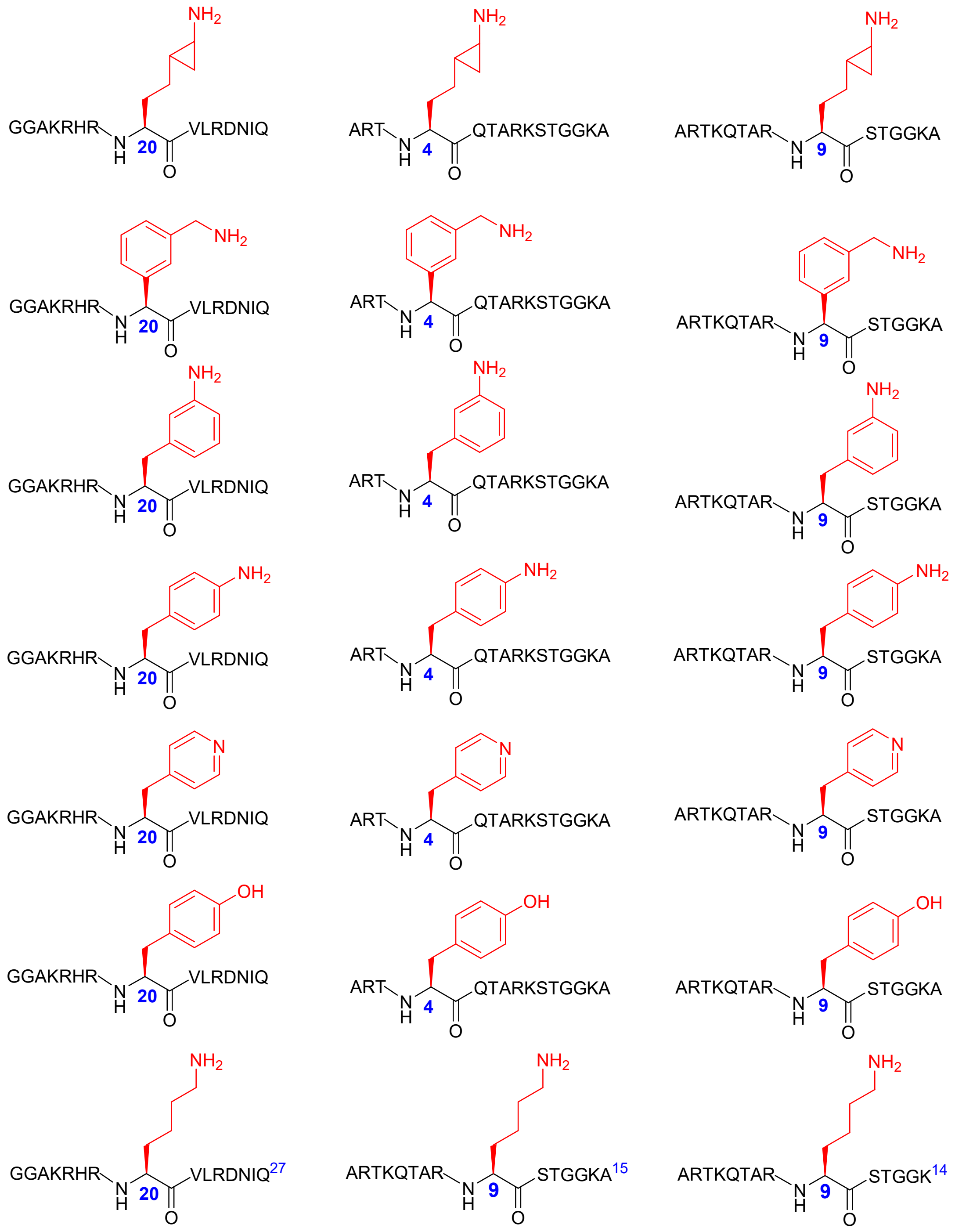

4. ESI-MS analysis of histone peptides. **Table S2.** MS analysis of sterically demanding histone peptides used in this study. All modified side chains are shown between the brackets and bolded.

Entry	Peptide	Sequence	Formula		m/z Calculated	m/z Found
1	H4K20	GGAKRHR**K**$^{\mathbf{20}}$VLRDNIQ	$C_{73}H_{130}N_{30}O_{20}$	$[M+H]^+$	1748.0	1748.2
2	H4K$_{cp}$20	GGAKRHR(**K$_{cp}$20**)VLRDNIQ	$C_{74}H_{130}N_{30}O_{20}$	$[M+H]^+$	1760.0	1760.9
3	H4Kba20	GGAKRHR(**K$_{ba}$20**)VLRDNIQ	$C_{76}H_{128}N_{30}O_{20}$	$[M+H]^+$	1782.0	1782.4
4	H4F$_{3a}$20	GGAKRHR(**F$_{3a}$20**)VLRDNIQ	$C_{76}H_{128}N_{30}O_{20}$	$[M+H]^+$	1782.0	1782.4
5	H4F$_{4a}$20	GGAKRHR(**F$_{4a}$20**)VLRDNIQ	$C_{76}H_{128}N_{30}O_{20}$	$[M+H]^+$	1782.0	1782.3
6	H4A$_{p}$20	GGAKRHR(**A$_{p}$20**)VLRDNIQ	$C_{75}H_{126}N_{30}O_{20}$	$[M+H]^+$	1769.0	1768.7
7	H4Y20	GGAKRHR(**Y20**)VLRDNIQ	$C_{76}H_{127}N_{29}O_{21}$	$[M+H]^+$	1783.0	1783.9
8	H3K4	ART**K**$^{\mathbf{4}}$QTARKSTGGKA	$C_{63}H_{118}N_{26}O_{20}$	$[M+H]^+$	1559.9	1559.8
9	H3K$_{cp}$4	ART(**K$_{cp}$4**)QTARKSTGGKA	$C_{64}H_{118}N_{26}O_{20}$	$[M+H]^+$	1572.9	1573.7
10	H3K$_{ba}$4	ART(**K$_{ba}$4**)QTARKSTGGKA	$C_{66}H_{116}N_{26}O_{20}$	$[M+H]^+$	1593.9	1594.3
11	H3F$_{3a}$4	ART(**F$_{3a}$4**)QTARKSTGGKA	$C_{66}H_{116}N_{26}O_{20}$	$[M+H]^+$	1593.9	1594.3
12	H3F$_{4a}$4	ART(**F$_{4a}$4**)QTARKSTGGKA	$C_{66}H_{116}N_{26}O_{20}$	$[M+H]^+$	1593.9	1594.2
13	H3A$_{p}$4	ART(**A$_{p}$4**)QTARKSTGGKA	$C_{65}H_{114}N_{26}O_{20}$	$[M+H]^+$	1579.9	1580.2
14	H3Y4	ART(**Y4**)QTARKSTGGKA	$C_{66}H_{115}N_{25}O_{21}$	$[M+H]^+$	1594.9	1595.2
15	H3K9	ARTKQTAR**K**$^{\mathbf{9}}$STGGKA	$C_{63}H_{117}N_{25}O_{21}$	$[M+H]^+$	1560.9	1561.2
16	H3K$_{cp}$9	ARTKQTAR(**K$_{cp}$9**)STGGKA	$C_{64}H_{117}N_{25}O_{21}$	$[M+H]^+$	1572.9	1573.7
17	H3K$_{cp}$9	ARTKQTAR(**K$_{ba}$9**)STGGKA	$C_{66}H_{115}N_{25}O_{21}$	$[M+H]^+$	1594.9	1594.8
18	H3F$_{3a}$9	ARTKQTAR(**F$_{3a}$9**)STGGKA	$C_{66}H_{115}N_{25}O_{21}$	$[M+H]^+$	1594.9	1594.8
19	H3F$_{4a}$9	ARTKQTAR(**F$_{4a}$9**)STGGKA	$C_{66}H_{115}N_{25}O_{21}$	$[M+H]^+$	1594.9	1594.8
20	H3A$_{p}$9	ARTKQTAR(**A$_{p}$9**)STGGKA	$C_{65}H_{113}N_{25}O_{21}$	$[M+H]^+$	1580.9	1580.8
21	H3Y9	ARTKQTAR(**Y9**)STGGKA	$C_{66}H_{114}N_{24}O_{22}$	$[M+H]^+$	1595.9	1595.8
22	14-mer H3K9	ARTKQTARKSTGGK	$C_{60}H_{112}N_{24}O_{20}$	$[M+H]^+$	1489.9	1489.0

5. Analytical HPLC chromatograms

A)

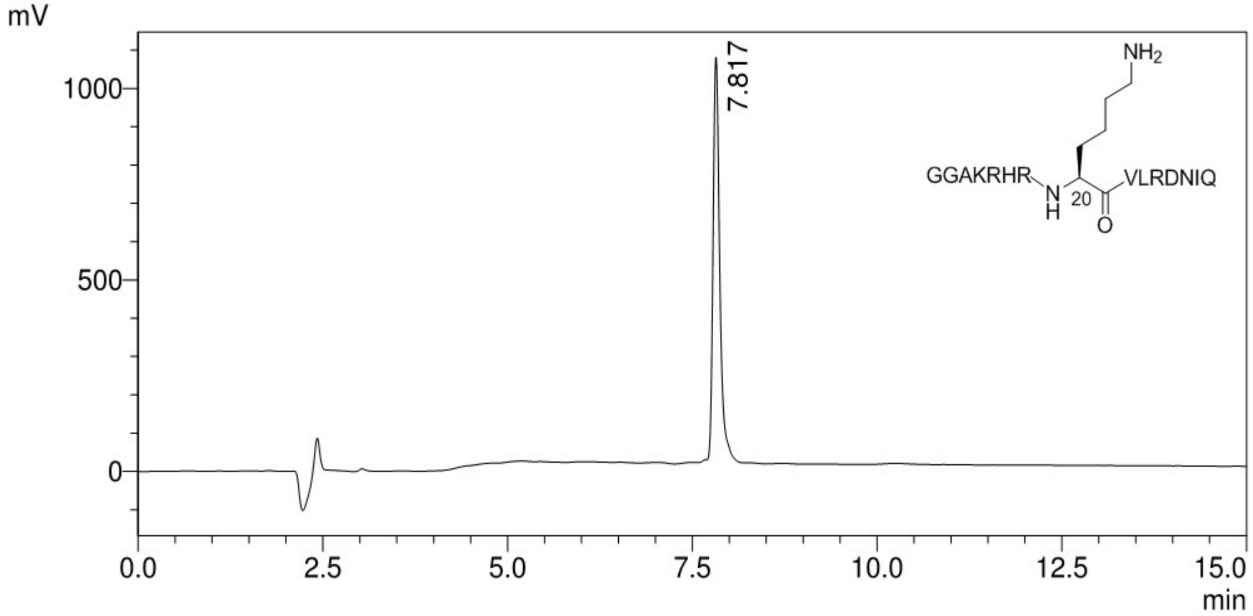

B)

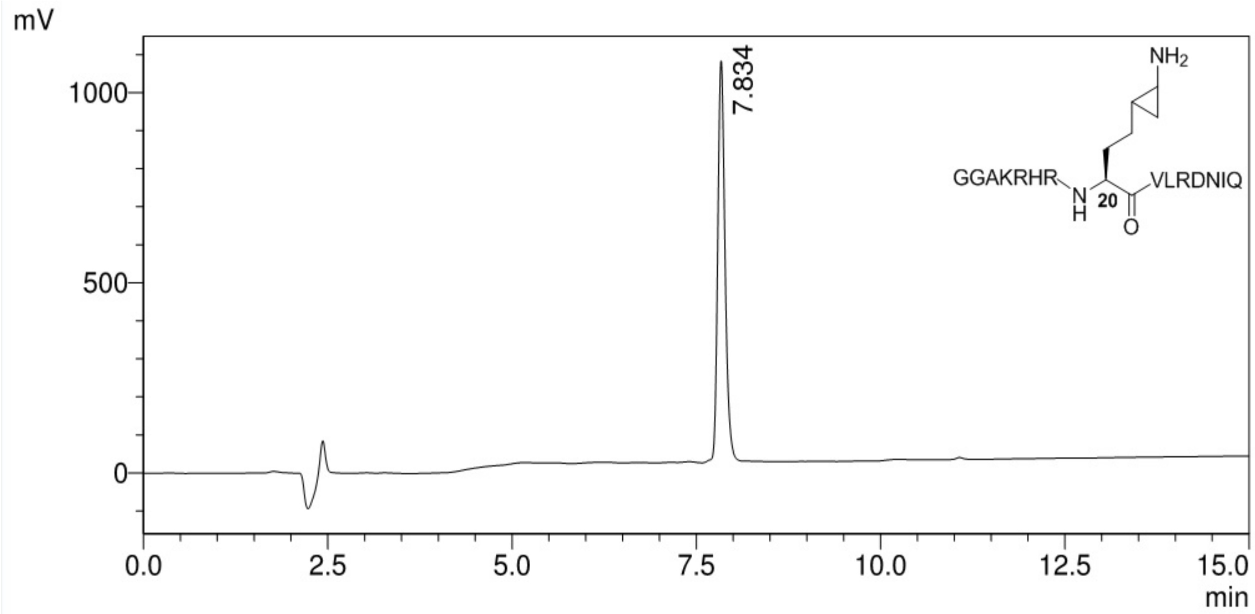

Figure S3. A) Analytical HPLC profile of H4K20 peptide after prep-HPLC purification. **B)** Analytical HPLC profile of H4K$_{CP}$20 peptide after prep-HPLC purification. Peak at 2.5 minute derives from injection (blank).

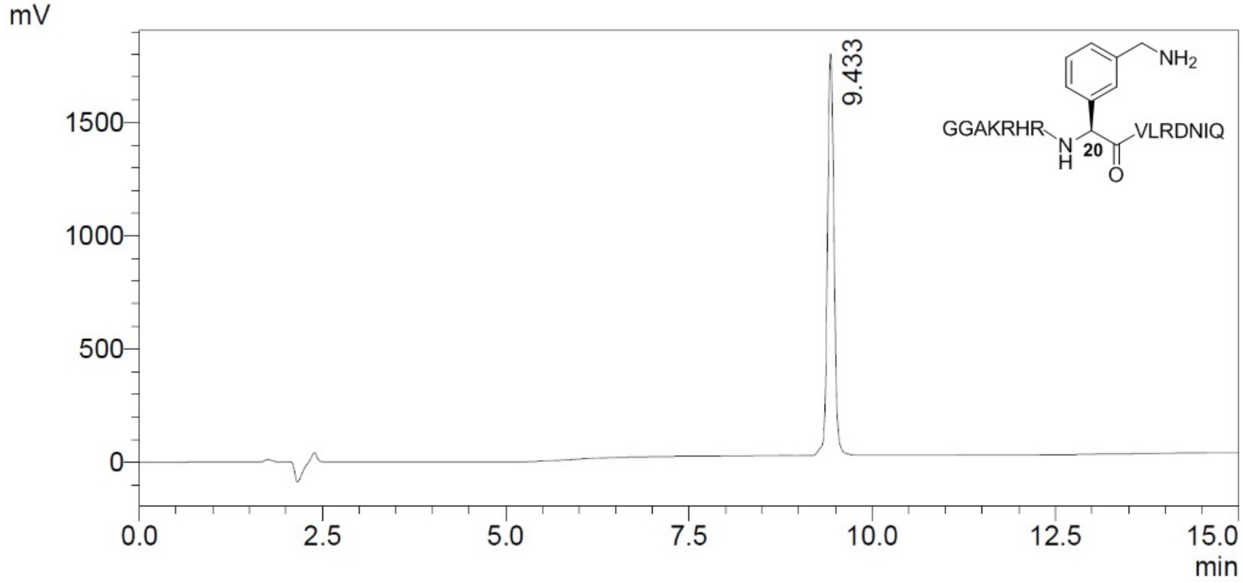

Figure S4. Analytical HPLC profile of $H4K_{ba}20$ peptide after prep-HPLC purification. Peak at 2.5 minute derives from injection (blank).

A)

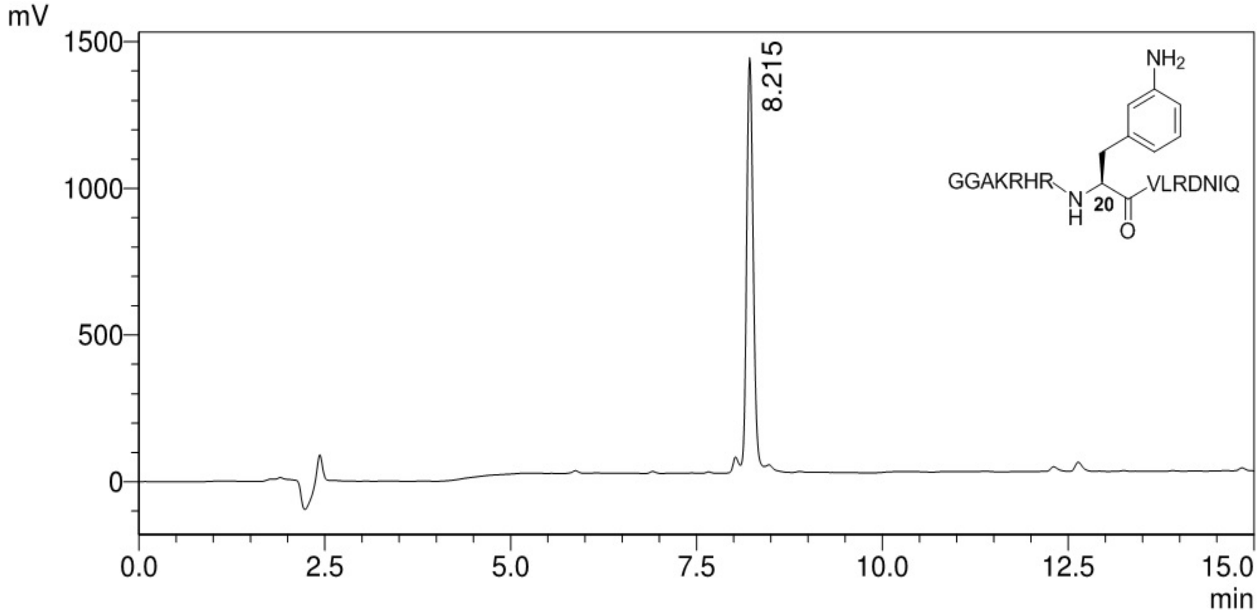

B)

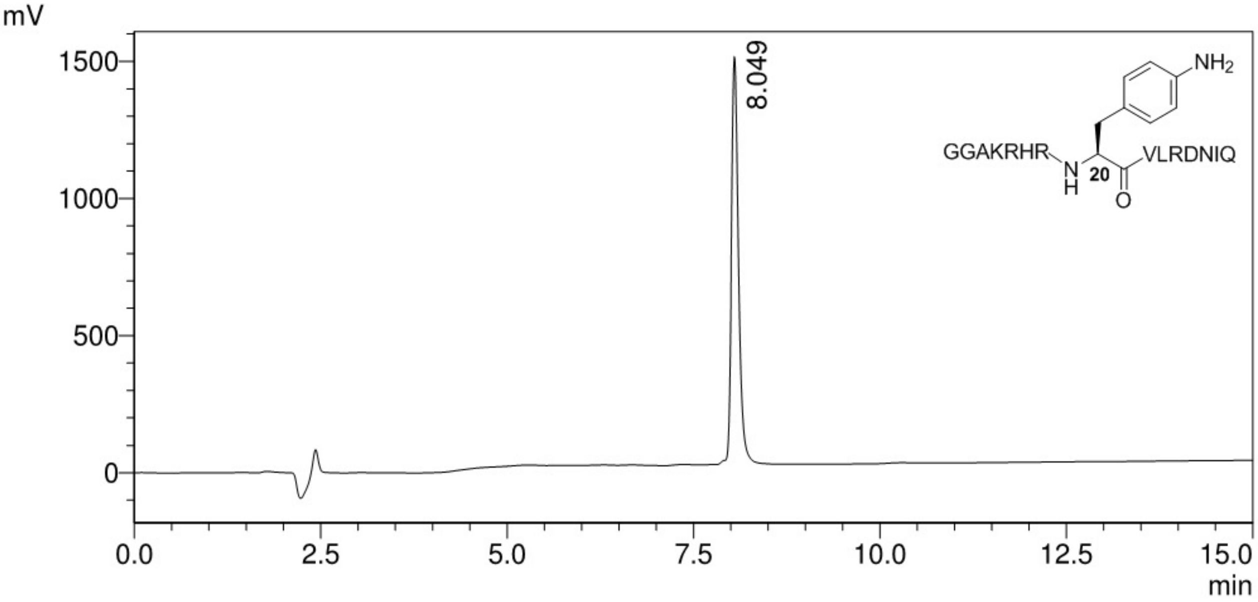

Figure S5. A) Analytical HPLC profile of $H4F_{3a}20$ peptide after prep-HPLC purification. **B)** Analytical HPLC profile of $H4F_{4a}20$ peptide after prep-HPLC purification. Peak at 2.5 minute derives from injection (blank).

A)

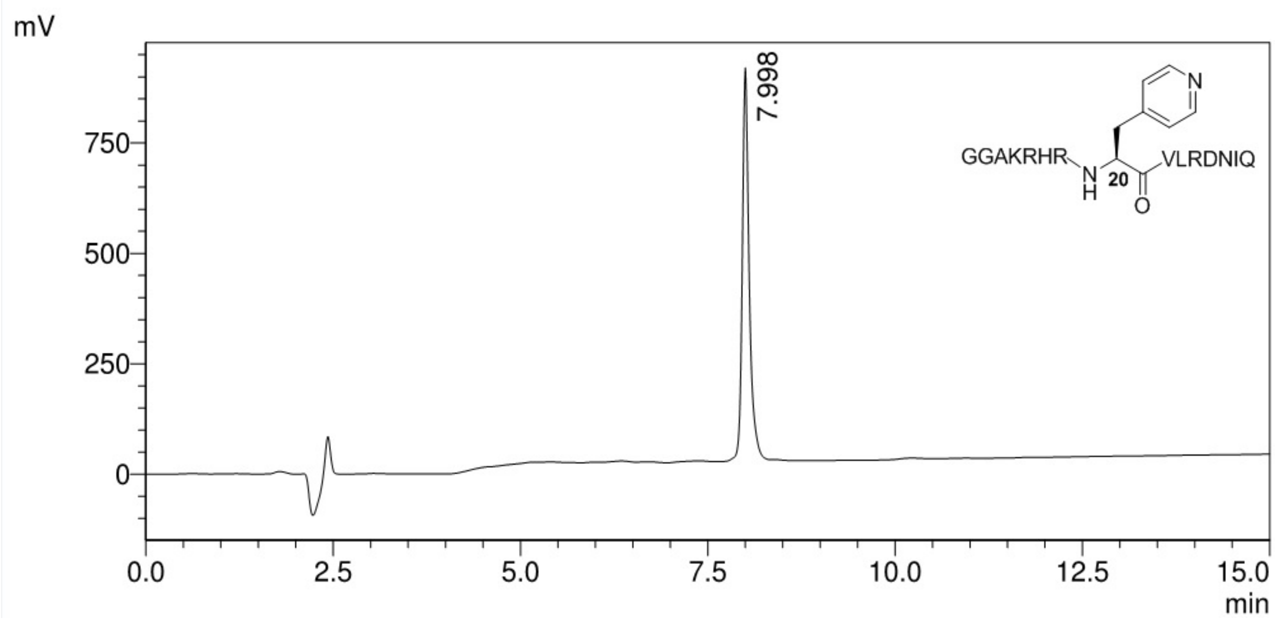

B)

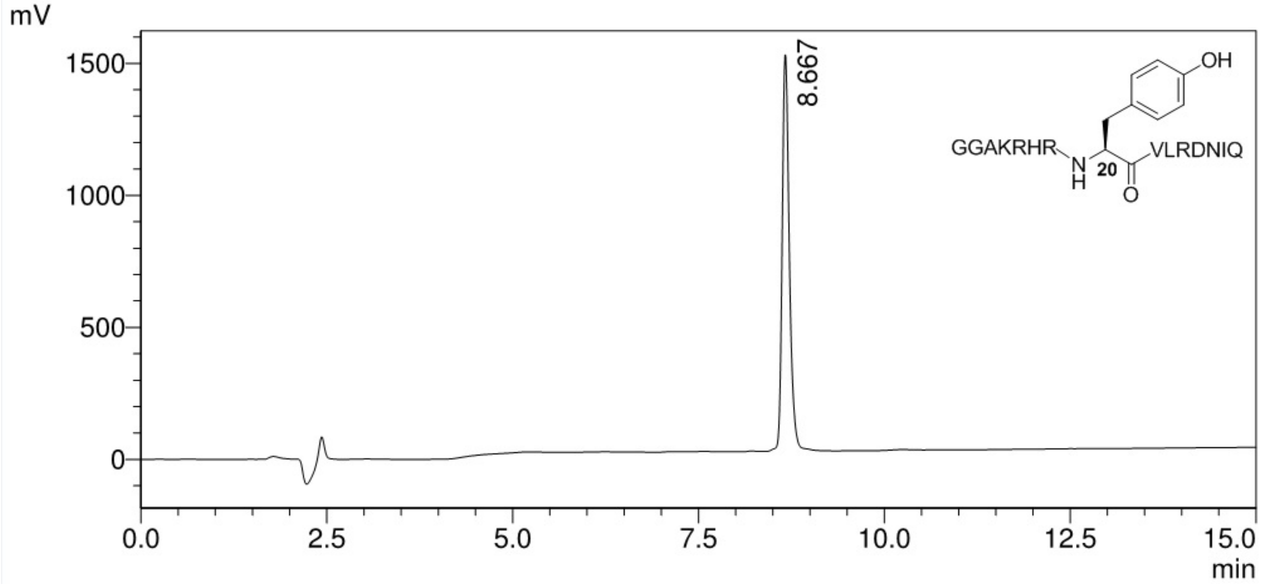

Figure S6. A) Analytical HPLC profile of H4A$_p$20 peptide after prep-HPLC purification. **B)** Analytical HPLC profile of H4Y20 peptide after prep-HPLC purification. Peak at 2.5 minute derives from injection (blank).

A)

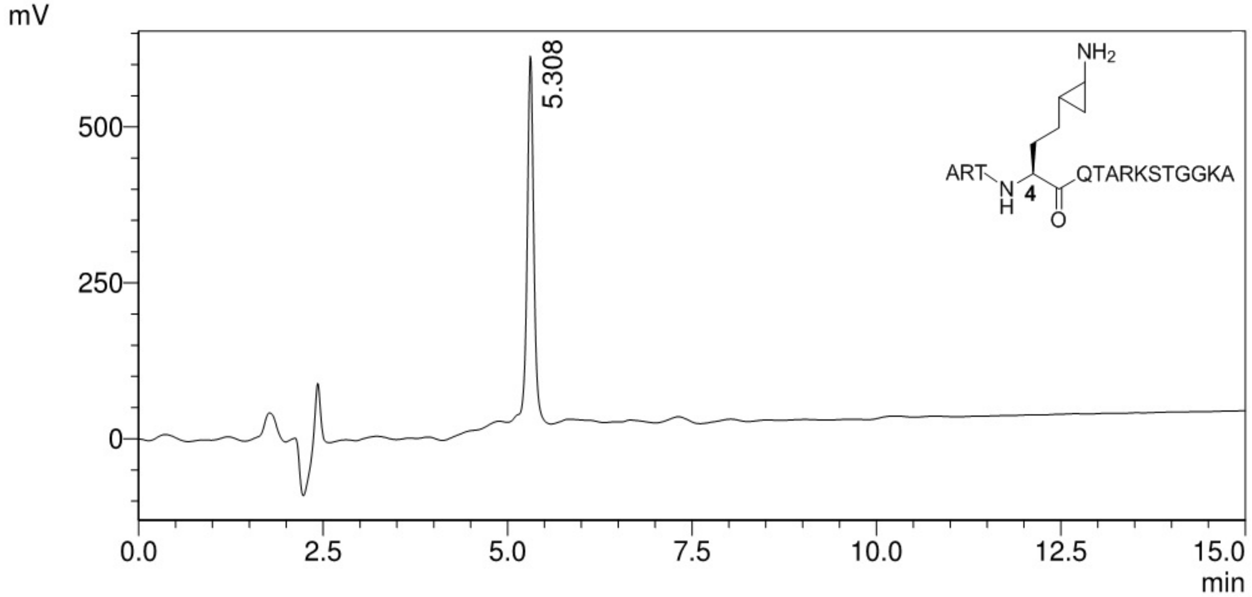

B)

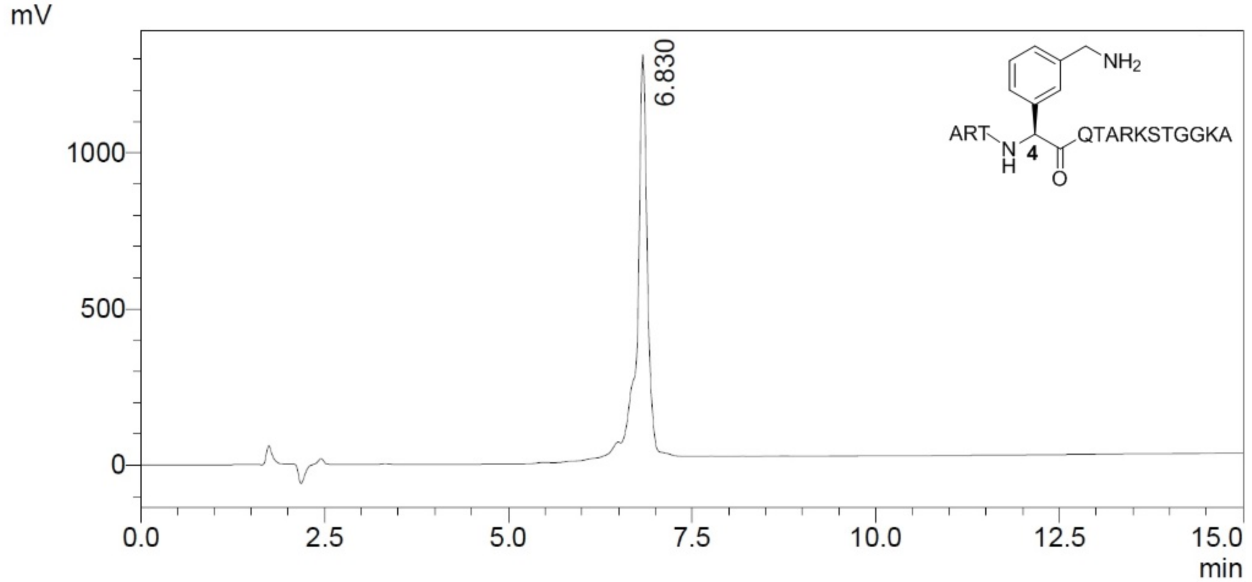

Figure S7. A) Analytical HPLC profile of $H3K_{CP}4$ peptide after prep-HPLC purification. **B)** Analytical HPLC profile of $H3K_{ba}4$ peptide after prep-HPLC purification. Peak at 2.5 minute derives from injection (blank).

A)

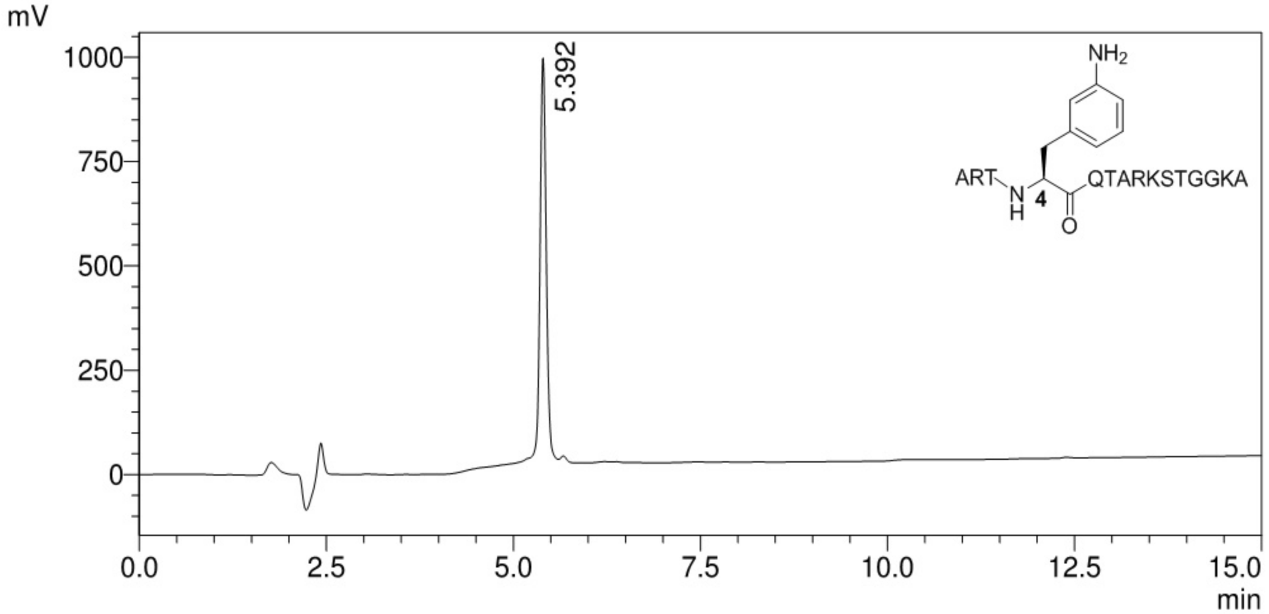

B)

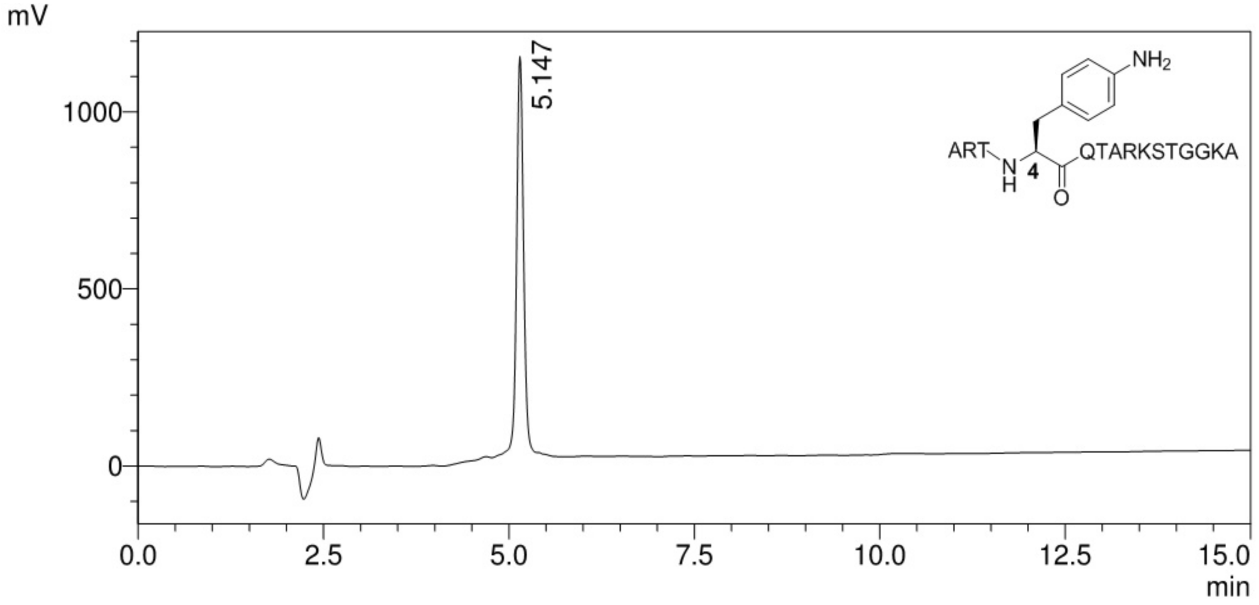

Figure S8. A) Analytical HPLC profile of H3F_{3a}4 peptide after prep-HPLC purification. **B)** Analytical HPLC profile of H3F_{4a}4 peptide after prep-HPLC purification. Peak at 2.5 minute derives from injection (blank).

A)

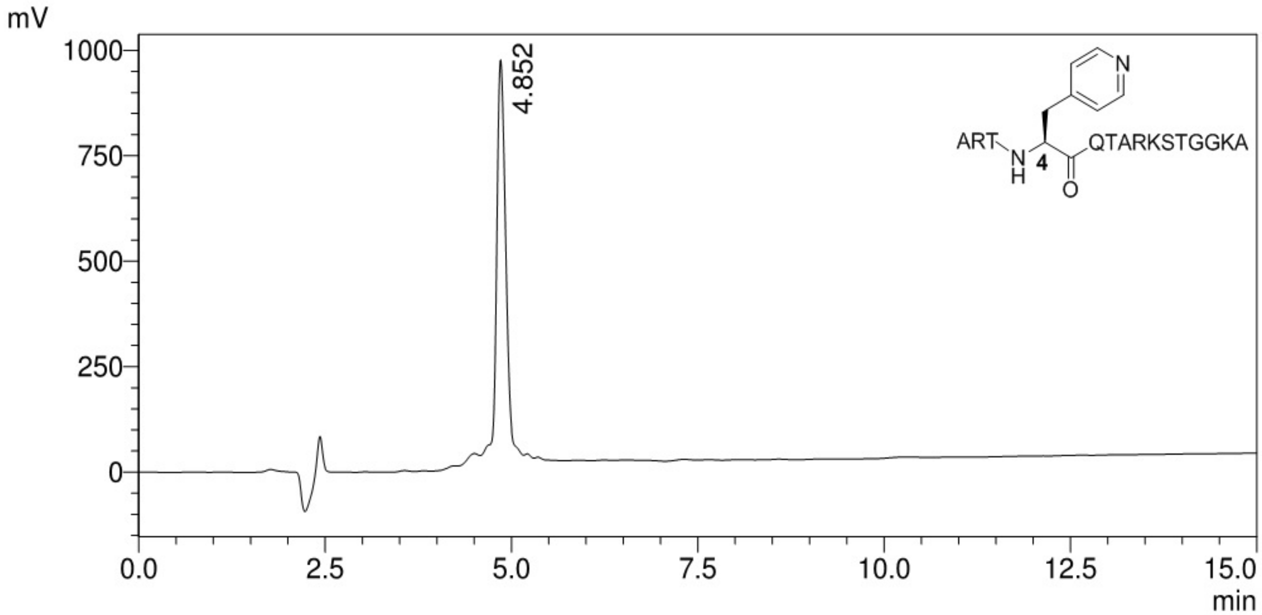

B)

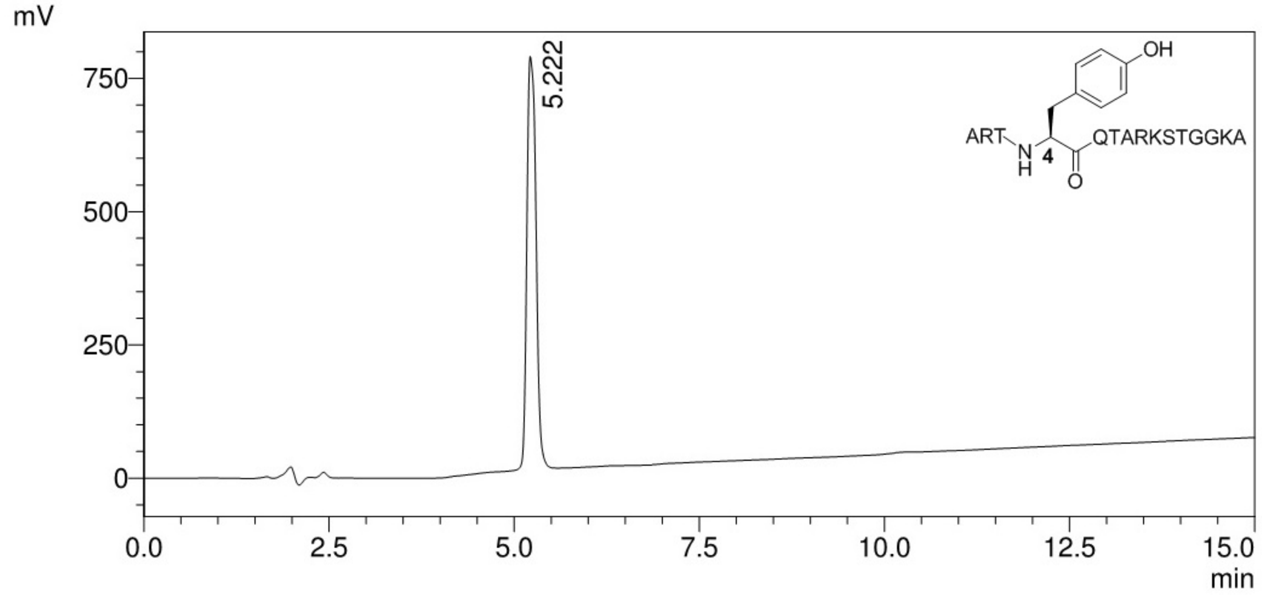

Figure S9. A) Analytical HPLC profile of H3A$_p$4 peptide after prep-HPLC purification. **B)** Analytical HPLC profile of H3Y4 peptide after prep-HPLC purification. Peak at 2.5 minute derives from injection (blank).

A)

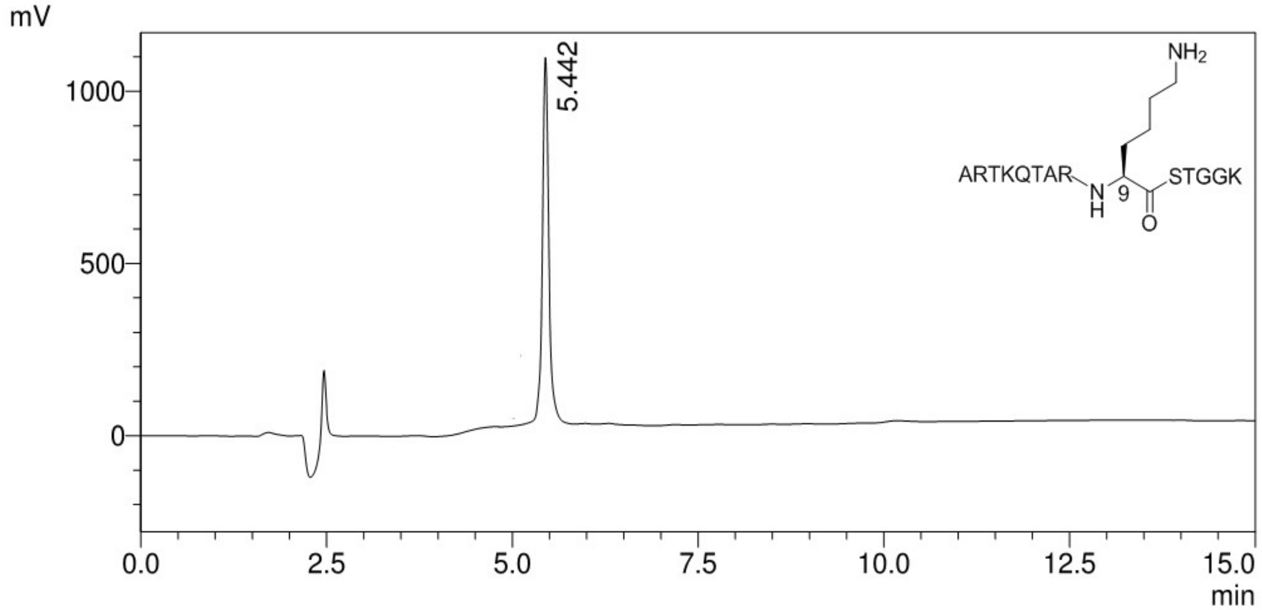

B)

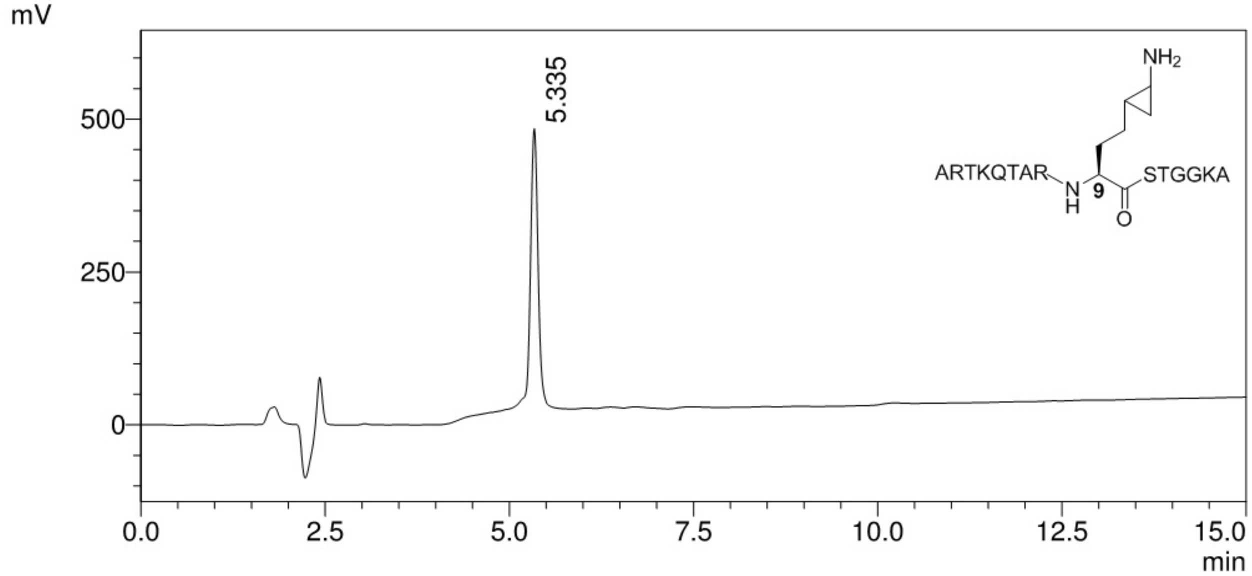

Figure S10. A) Analytical HPLC profile of H3K9 peptide after prep-HPLC purification. **B)** Analytical HPLC profile of $H3K_{CP}9$ peptide after prep-HPLC purification. Peak at 2.5 minute derives from injection (blank).

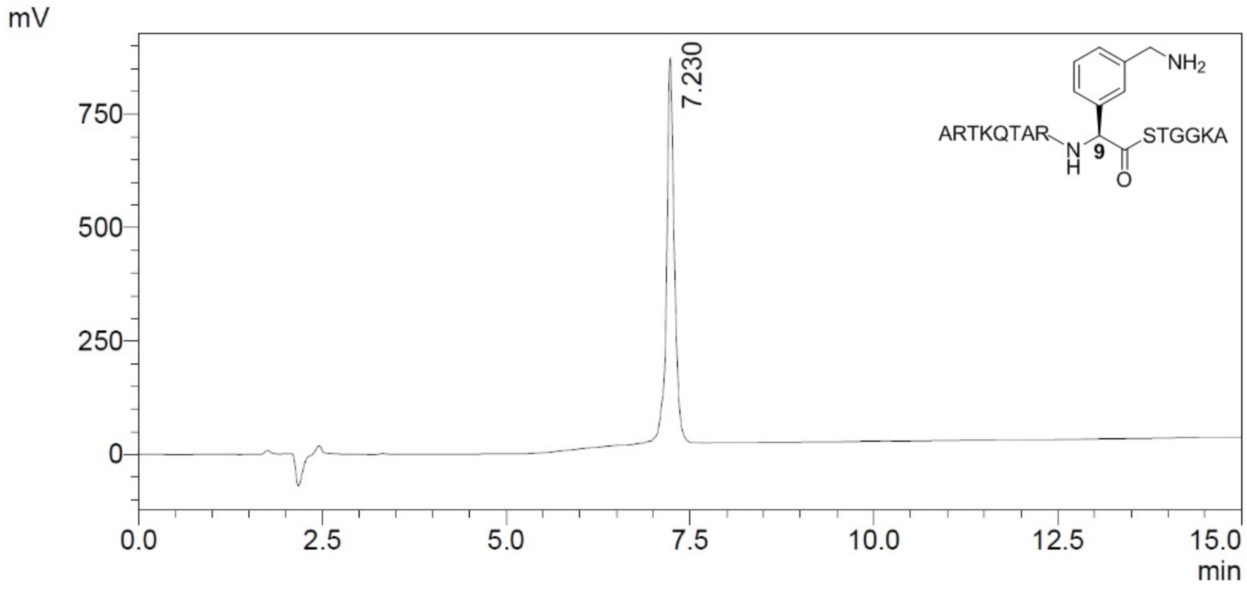

Figure S11. Analytical HPLC profile of H3K$_{ba}$9 peptide after prep-HPLC purification. Peak at 2.5 minute derives from injection (blank).

A)

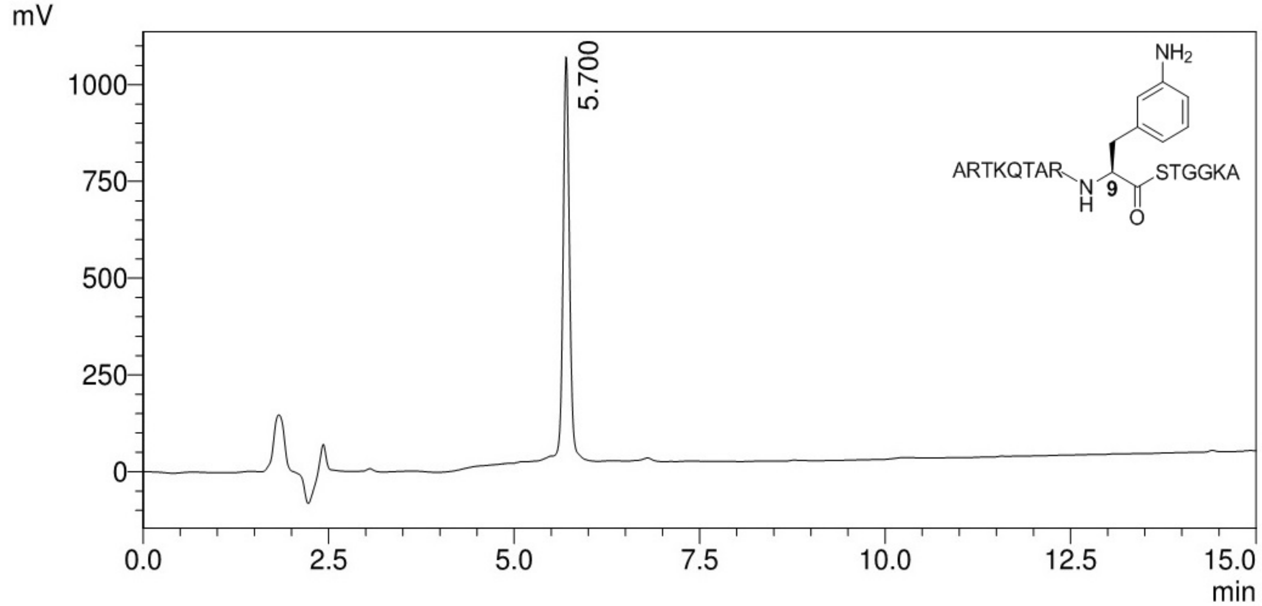

B)

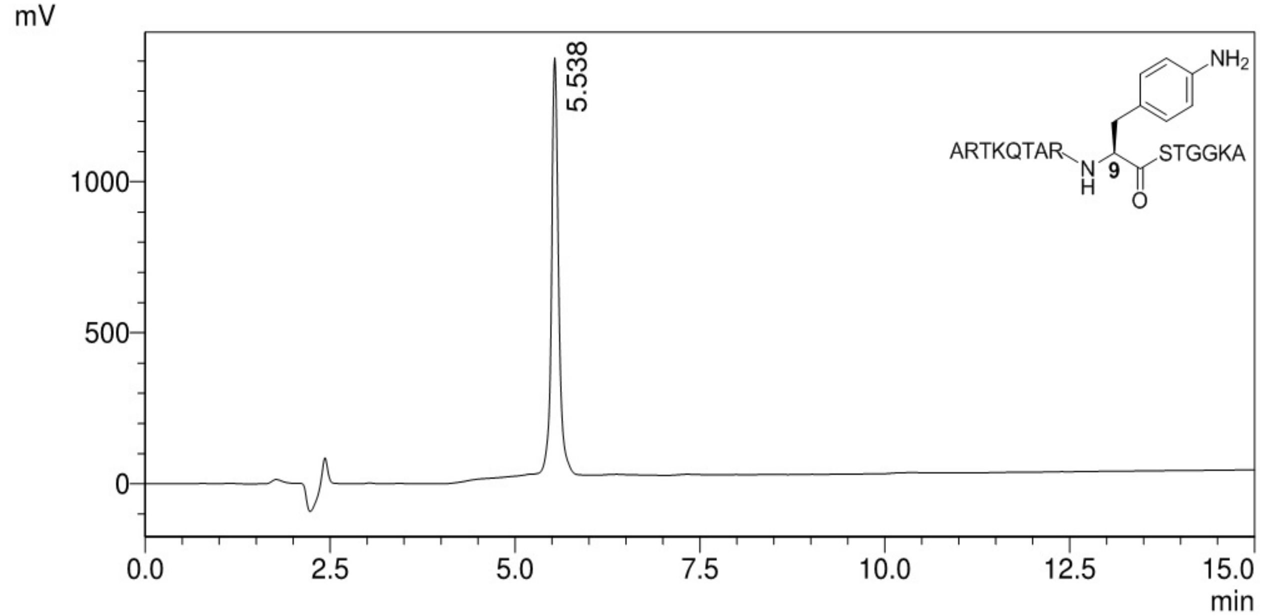

Figure S12. A) Analytical HPLC profile of $H3F_{3a}9$ peptide after prep-HPLC purification. **B)** Analytical HPLC profile of $H3F_{4a}9$ peptide after prep-HPLC purification. Peak at 2.5 minute derives from injection (blank).

A)

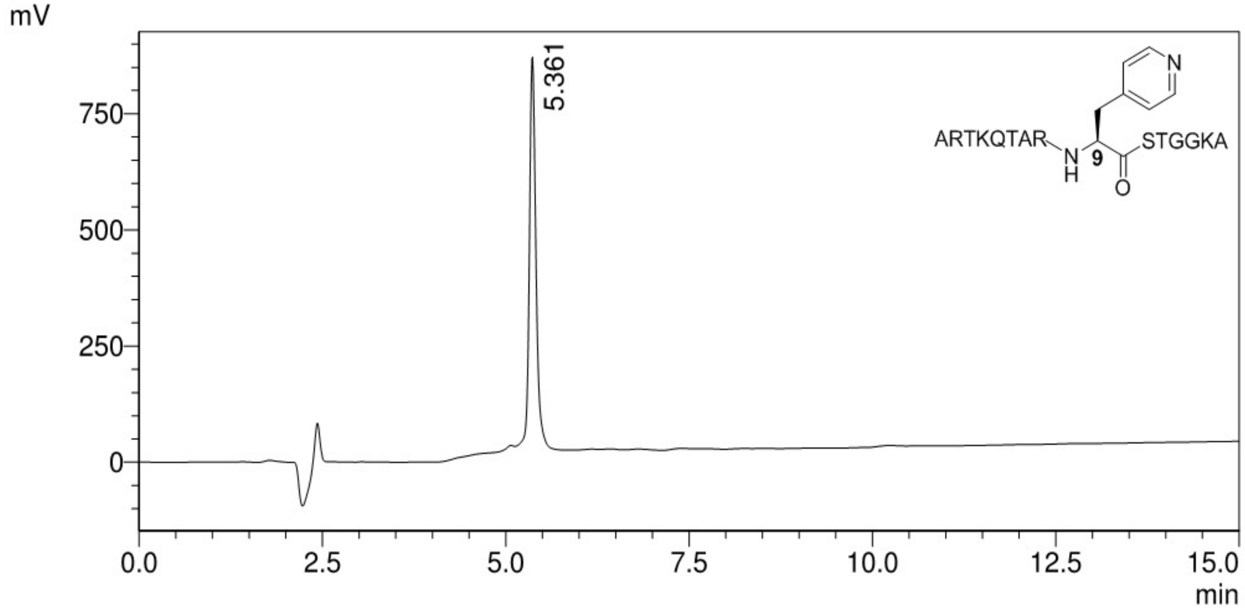

B)

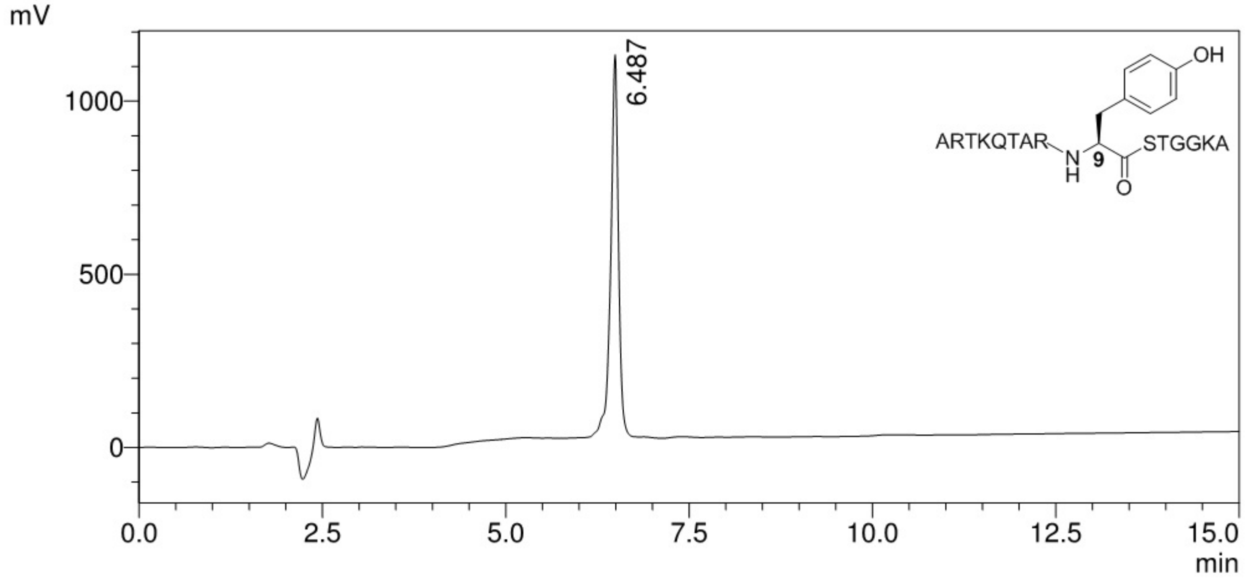

Figure S13. A) Analytical HPLC profile of H3A$_p$9 peptide after prep-HPLC purification. **B)** Analytical HPLC profile of H3Y9 peptide after prep-HPLC purification. Peak at 2.5 minute derives from injection (blank).

6. MALDI-TOF supporting figures

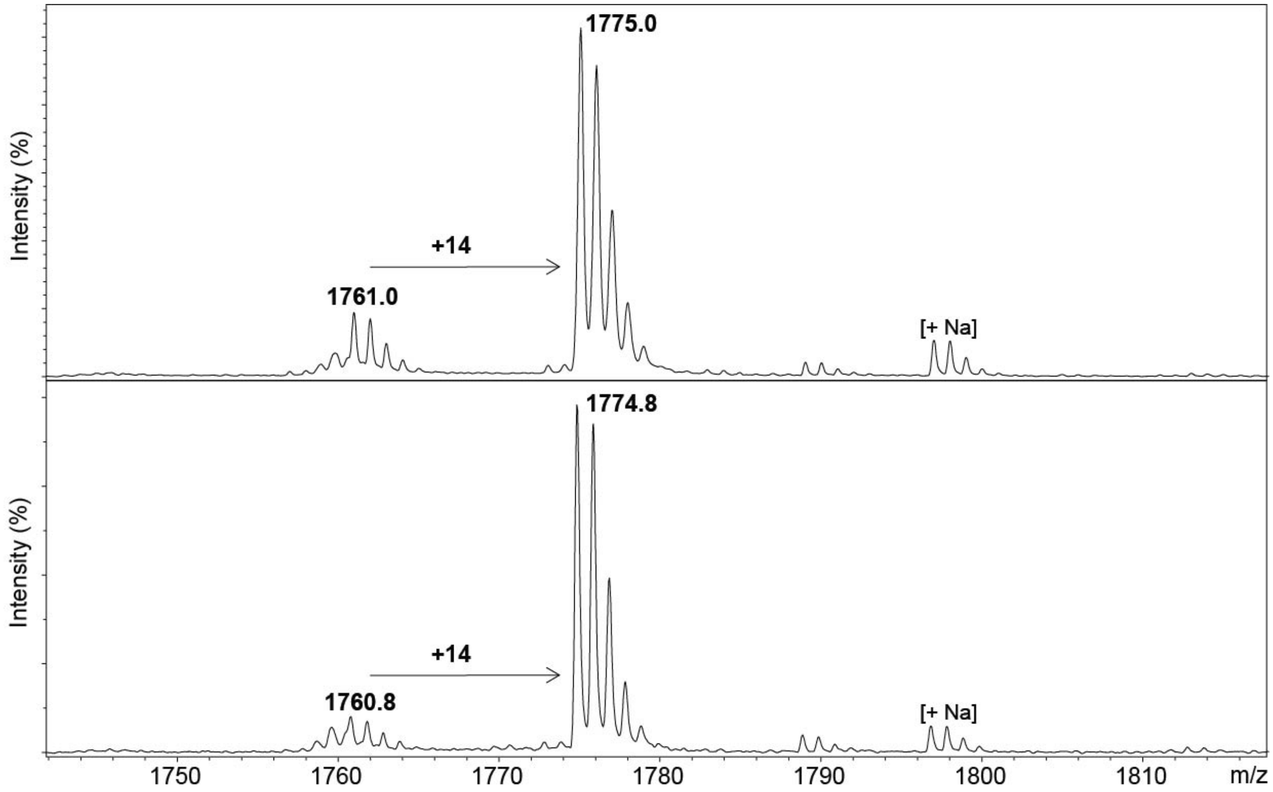

Figure S14. MALDI-MS spectra of methylation assay showing SETD8 (10 μM) catalyzed predominant monomethylation of H4K$_{CP}$20 peptide (100 μM) in the presence of SAM (1 mM), after 1 hour (top panel) and 5 hours (bottom panel) at 37 °C.

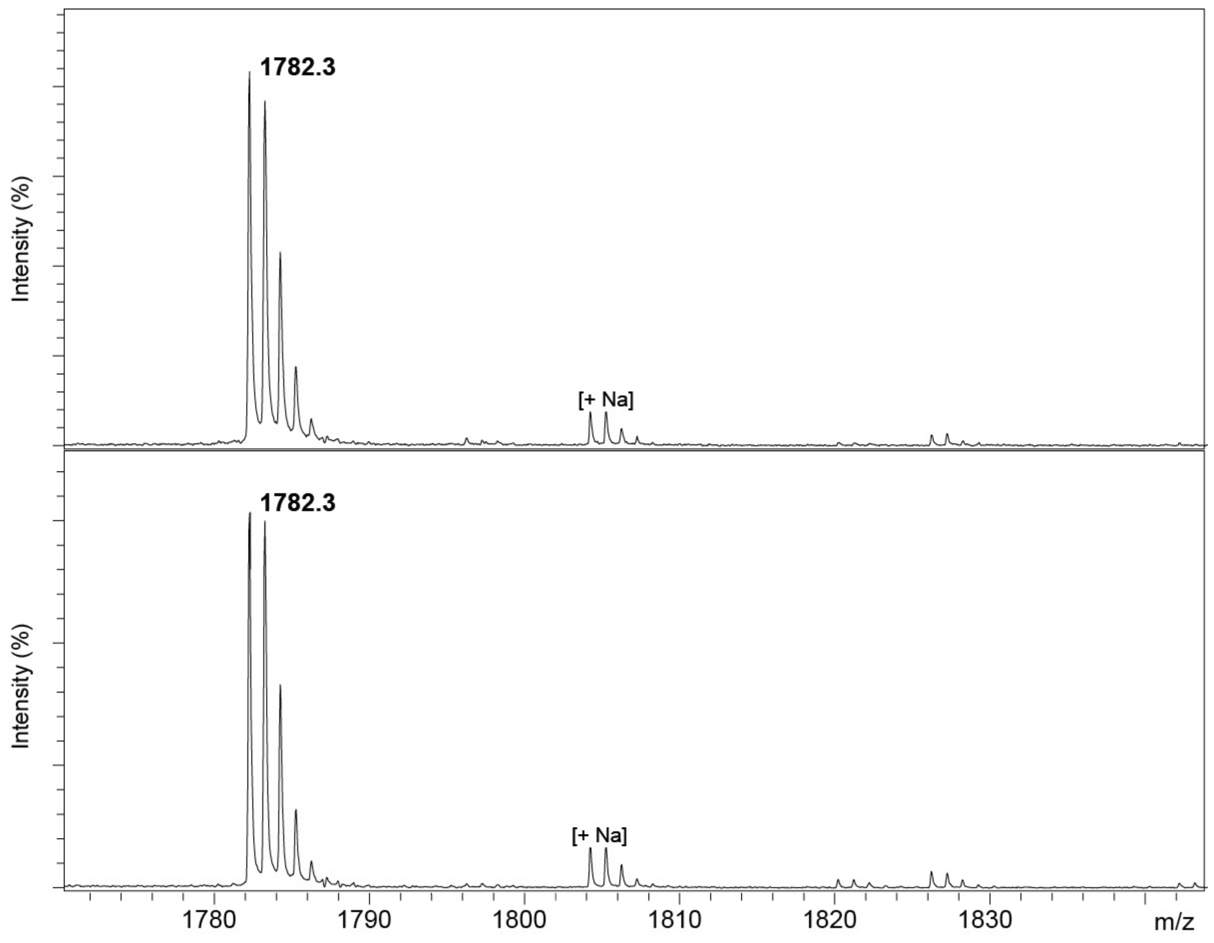

Figure S15. MALDI-MS spectra of methylation assay showing SETD8 (10 μM) catalyzed predominant monomethylation of $H4K_{ba}20$ peptide (100 μM) in the presence of SAM (1 mM), after 1 hour (top panel) and 5 hours (bottom panel) at 37 °C.

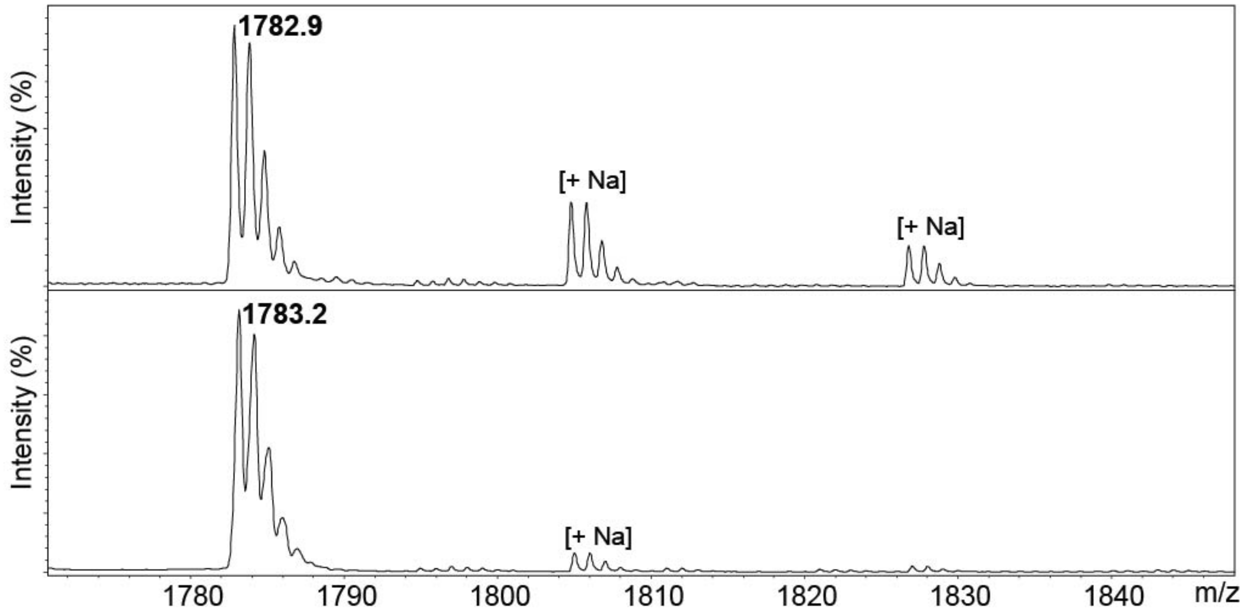

Figure S16. MALDI-MS analysis showing a lack of methylation for H4F$_{3a}$20 peptide (100 μM) in the presence of SETD8 (10 μM) and SAM (1 mM) after incubation for 1 hour (top panel) and 5 hours (bottom panel) at 37 °C.

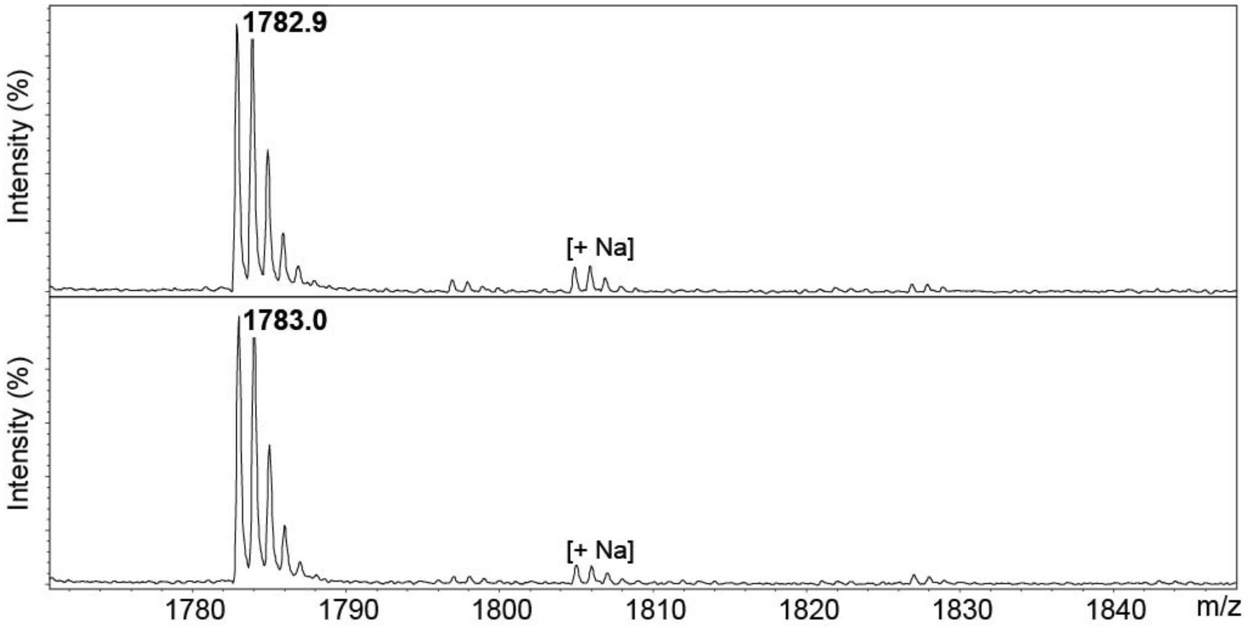

Figure S17. MALDI-MS analysis showing a lack of methylation for H4F$_{4a}$20 peptide (100 μM) in the presence of SETD8 (10 μM) and SAM (1 mM) after incubation for 1 hour (top panel) and 5 hours (bottom panel) at 37 °C.

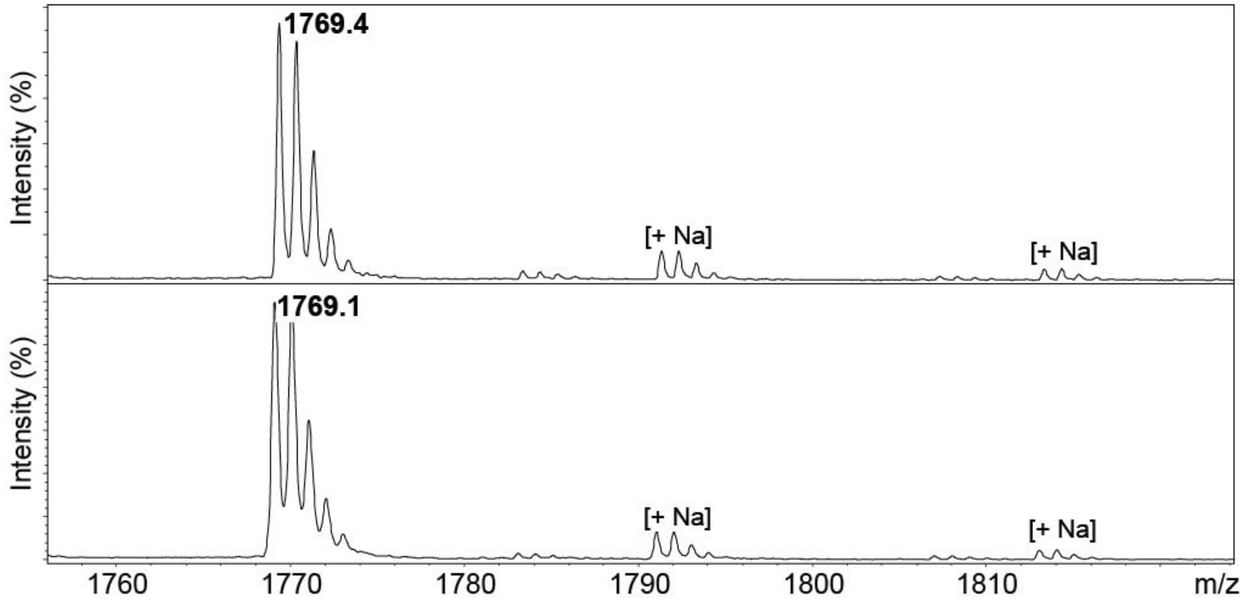

Figure S18. MALDI-MS analysis showing a lack of methylation for H4A_p20 peptide (100 µM) in the presence of SETD8 (10 µM) and SAM (1 mM) after incubation for 1 hour (top panel) and 5 hours (bottom panel) at 37 °C.

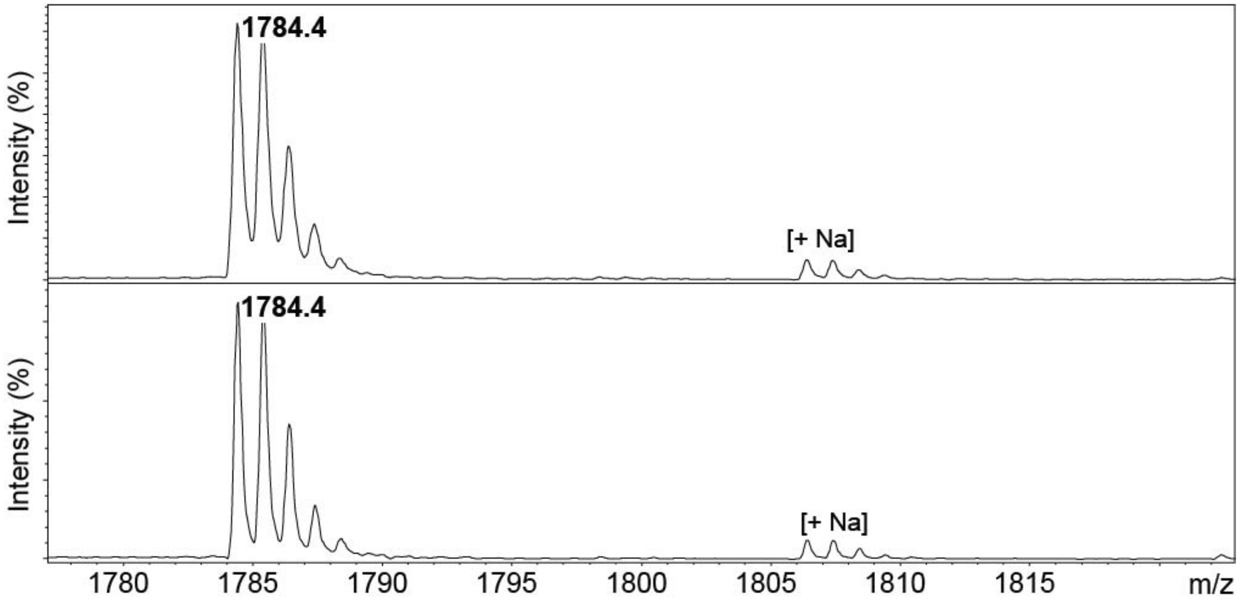

Figure S19. MALDI-MS analysis showing a lack of methylation for H4Y20 peptide (100 µM) in the presence of SETD8 (10 µM) and SAM (1 mM) after incubation for 1 hour (top panel) and 5 hours (bottom panel) at 37 °C.

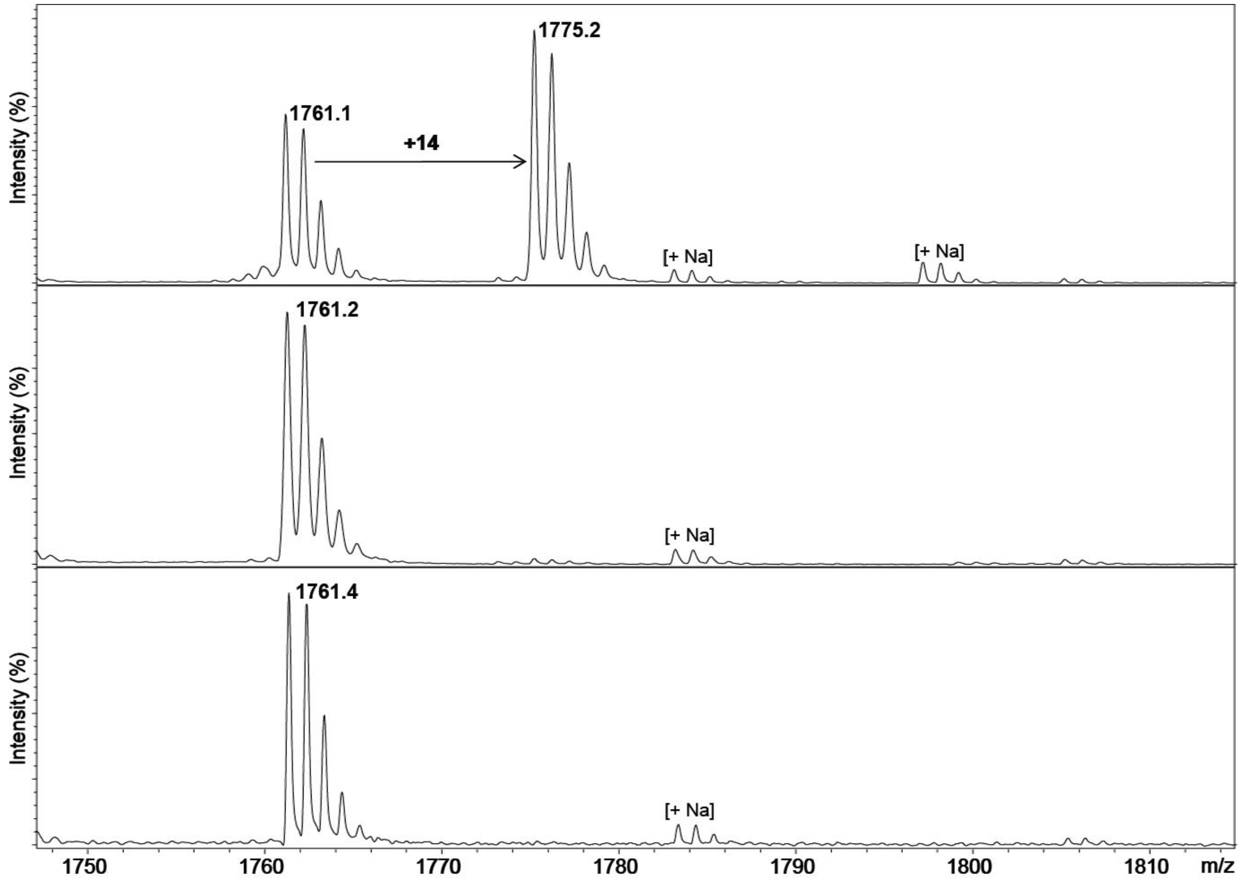

Figure S20. MALDI-MS spectra of methylation assay showing SETD8 (2 μM) catalyzed methylation of H4K_{CP}20 peptide (100 μM) in the presence of SAM (200 μM) after 1 hour at 37 °C (top panel). Control reaction in the absence of SETD8 (middle panel). Control reaction in the absence of SAM (bottom panel).

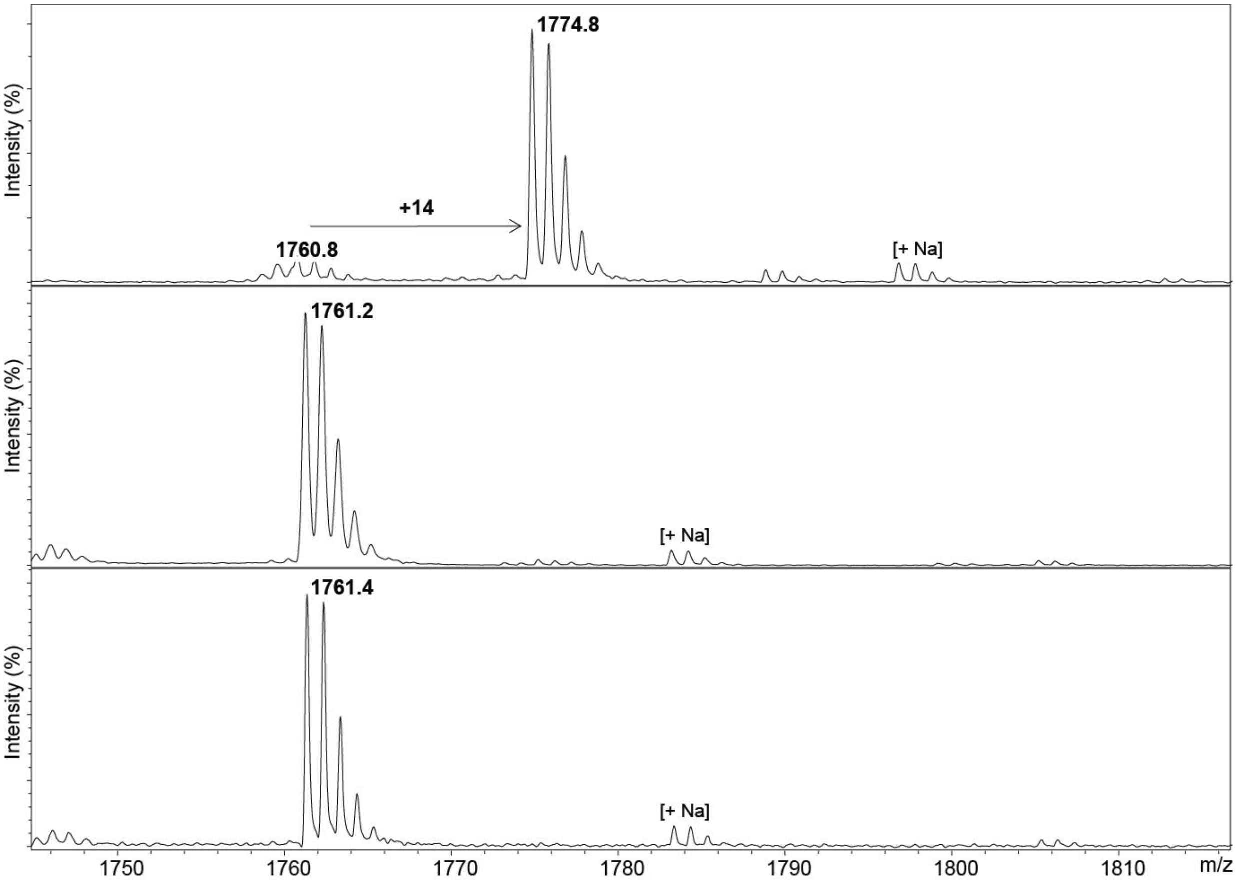

Figure S21. MALDI-MS spectra of methylation assay showing SETD8 (10 μM) catalyzed predominant monomethylation of H4K_{CP}20 peptide (100 μM) in the presence of SAM (1 mM), after 1 hour at 37 °C (top panel). Control reaction in the absence of SETD8 (middle panel). Control reaction in the absence of SAM (bottom panel).

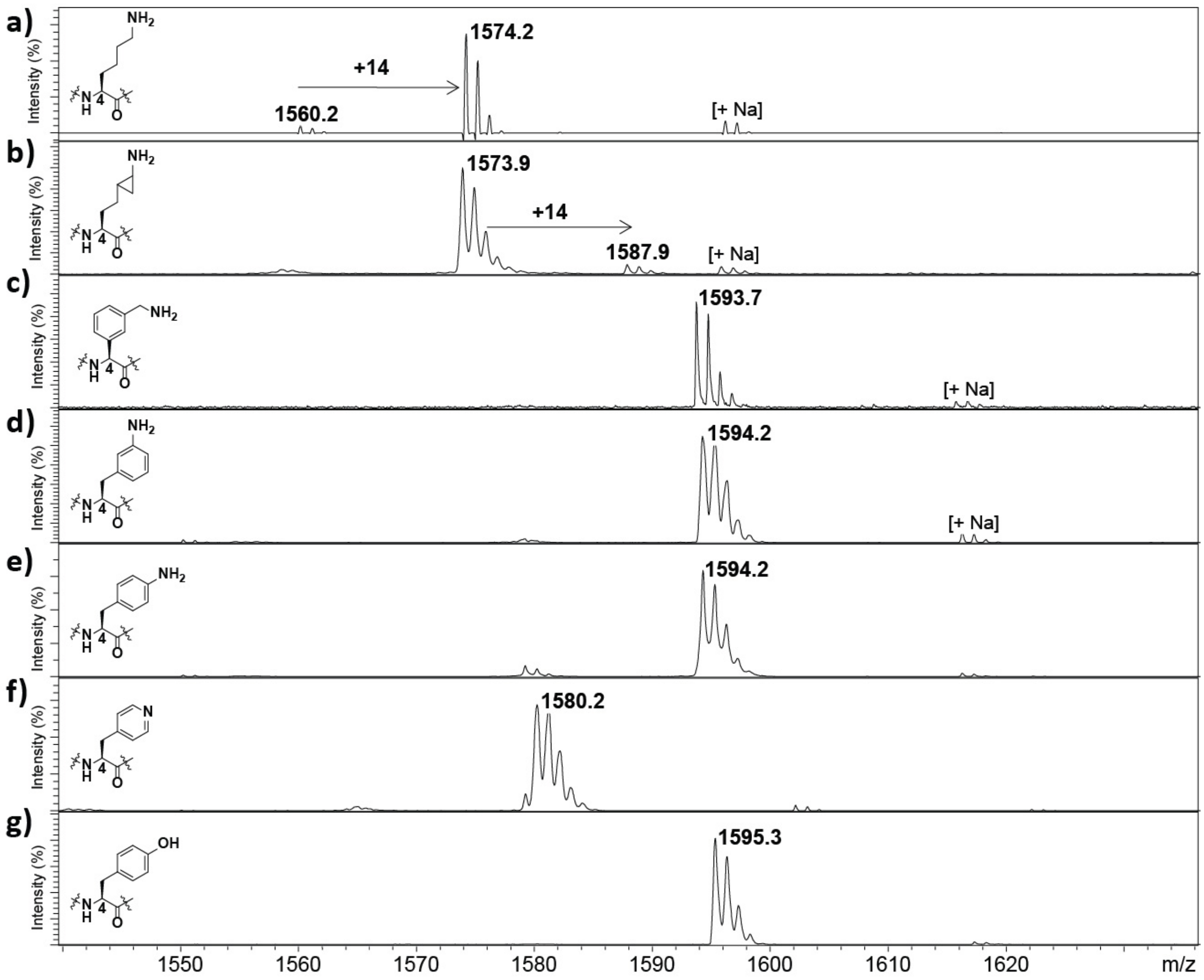

Figure S22. MALDI-MS spectra of methylation assay showing (**a**) monomethylation of H3K4; (**b**) a traces of monomethylation of H3K$_{CP}$4; (**c**) a lack of methylation for H3Kba4; (**d**) H3F$_{3a}$4; (**e**) H3F$_{4a}$4; (**f**) H3A$_{p}$4; and (**g**) H3Y4 peptide (100 µM) in the presence of SETD7 (2 µM) and SAM (200 µM) after incubation for 1 hour at 37 °C.

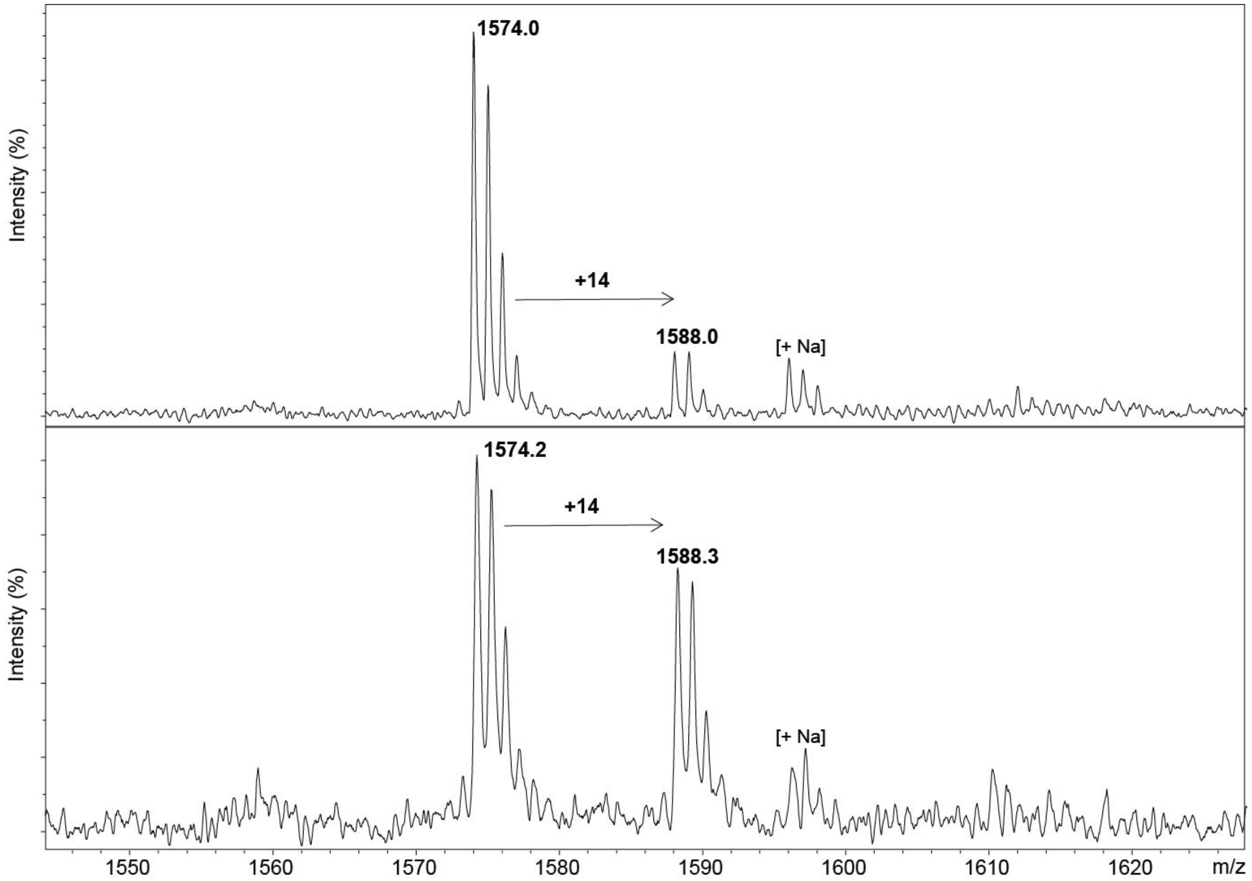

Figure S23. MALDI-MS spectra of methylation assay showing SETD7 (10 μM) catalyzed monomethylation of H3K$_{CP}$4 peptide (100 μM) in the presence of SAM (1 mM) after 1 hour (top panel) and 3 hours (bottom panel) at 37 °C.

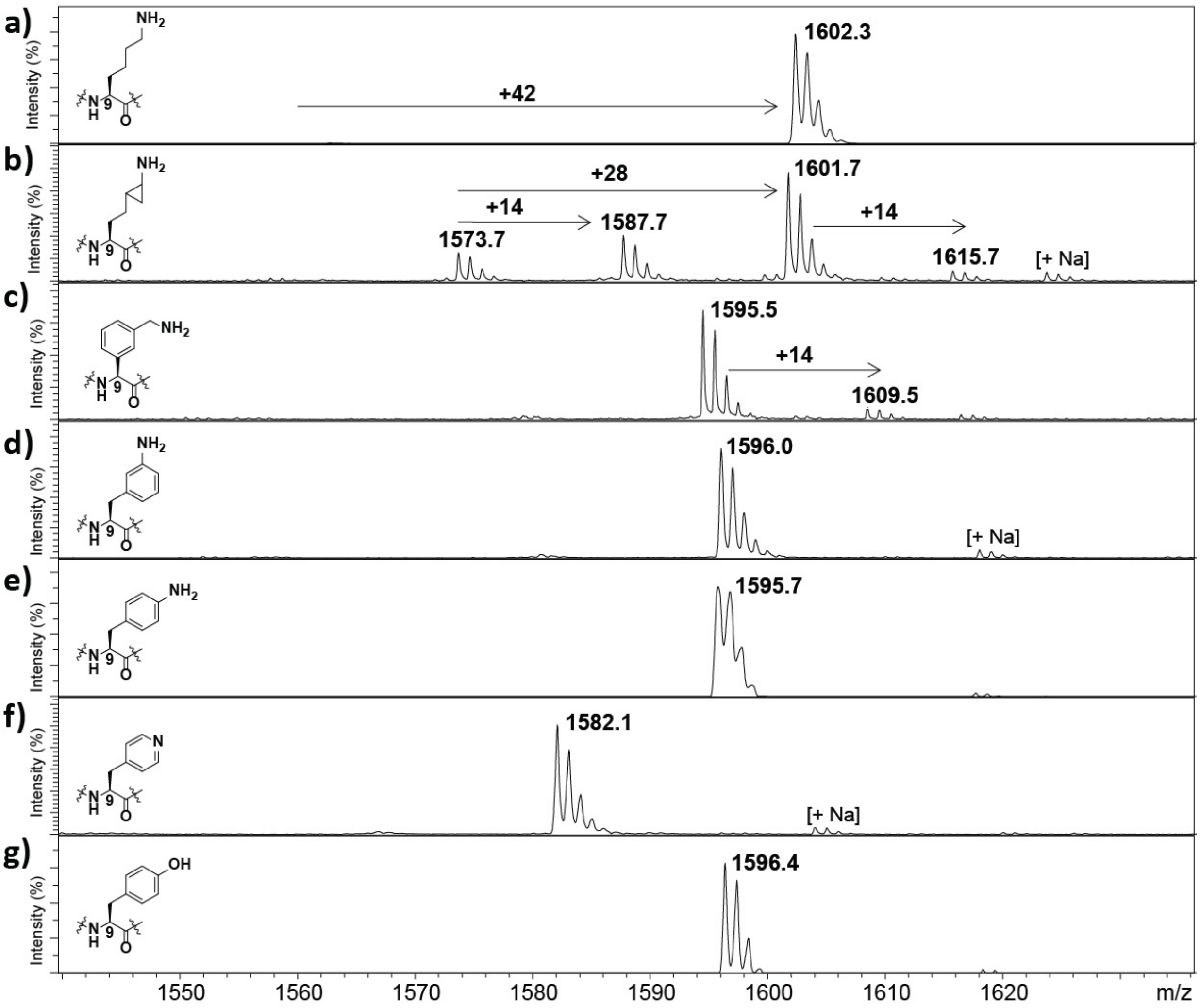

Figure S24. MALDI-MS spectra of methylation assay showing (**a**) trimethylation of H3K9; (**b**) mono-, di-, and trimethylation of H3K$_{CP}$9; (**c**) a trace of monomethylation of H3K$_{ba}$9; (**d**) a lack of methylation for H3F$_{3a}$9; (**e**) H3F$_{4a}$9; (**f**) H3A$_{p}$9; and (**g**) H3Y9 peptide (100 μM) in the presence of G9a (2 μM) and SAM (500 μM) after incubation for 1 hour at 37 °C.

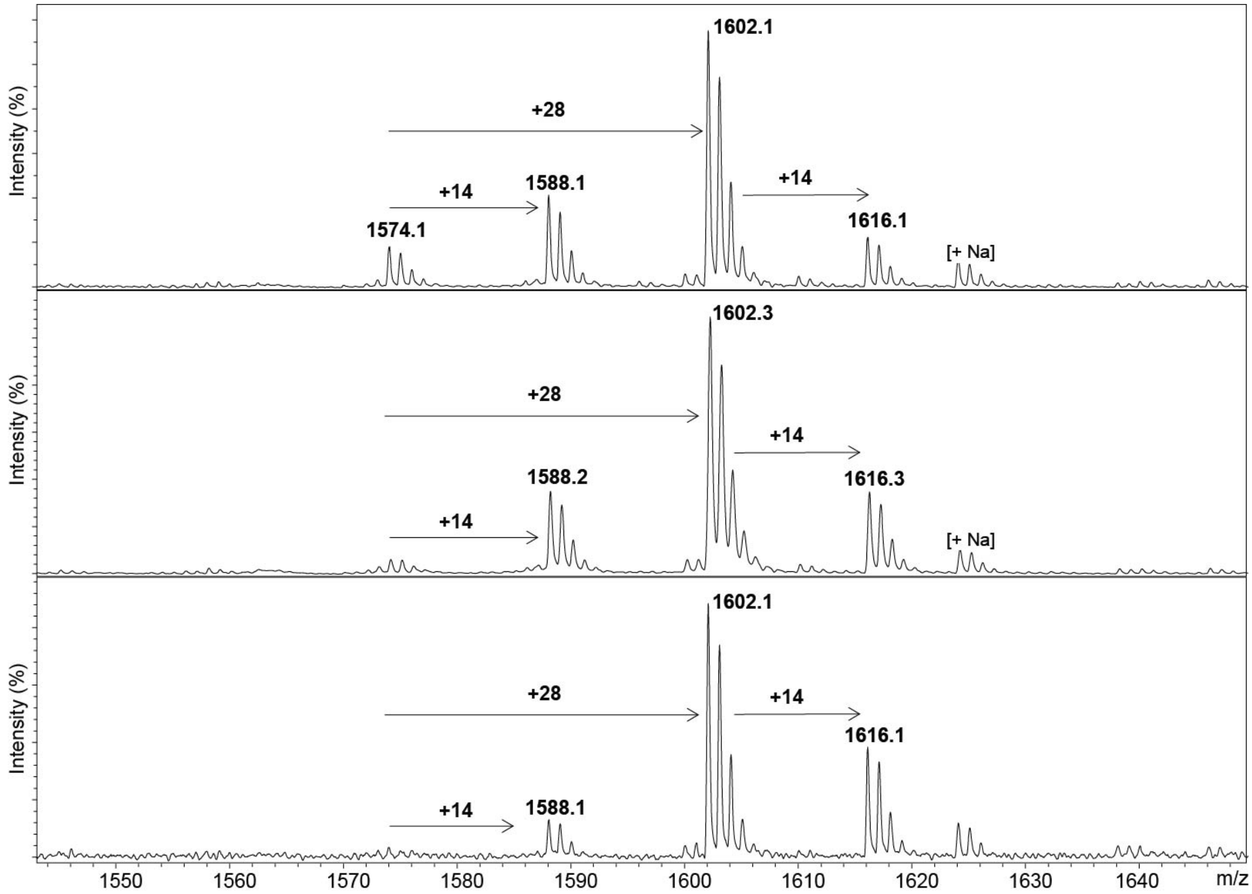

Figure S25. MALDI-MS spectra of methylation assay showing GLP (2 μM) catalyzed mono-, di-, and trimethylation of H3K$_{CP}$9 peptide (100 μM) in the presence of SAM (500 μM) after 1 hour at 37 °C (top panel). The formation of trimethylation of H3K$_{cp}$9 peptide increased after 2 hours (middle panel) and after 3 hours (bottom panel).

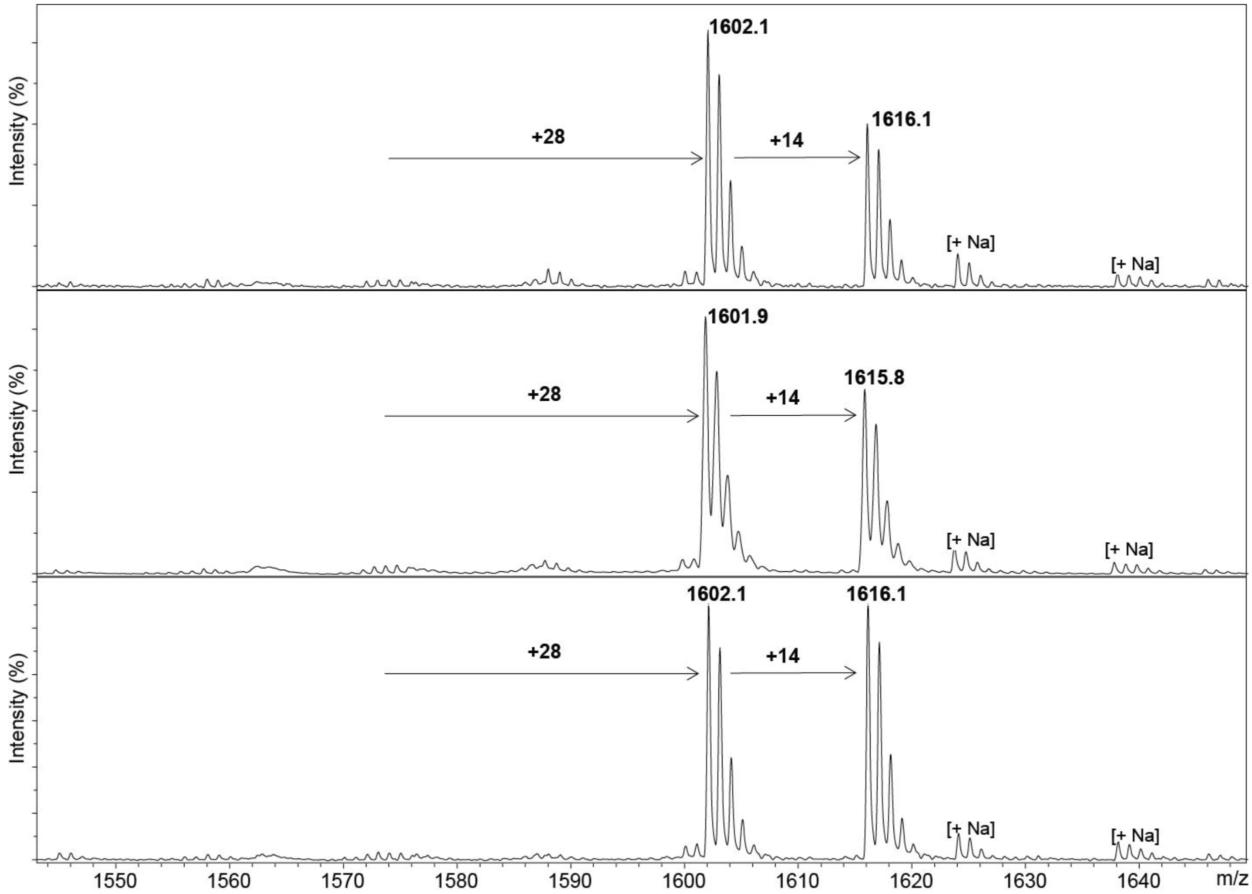

Figure S26. MALDI-MS spectra of methylation assay showing G9a (2 μM) catalyzed mono-, di, and trimethylation of H3K$_{CP}$9 peptide (100 μM) in the presence of SAM (500 μM) after 1 hour at 37 °C (top panel). A mixture of major dimethylation and trimethylation were observed after 2 hours (middle panel). A mixture of major equal dimethylation and trimethylation were observed after 3 hours (bottom panel).

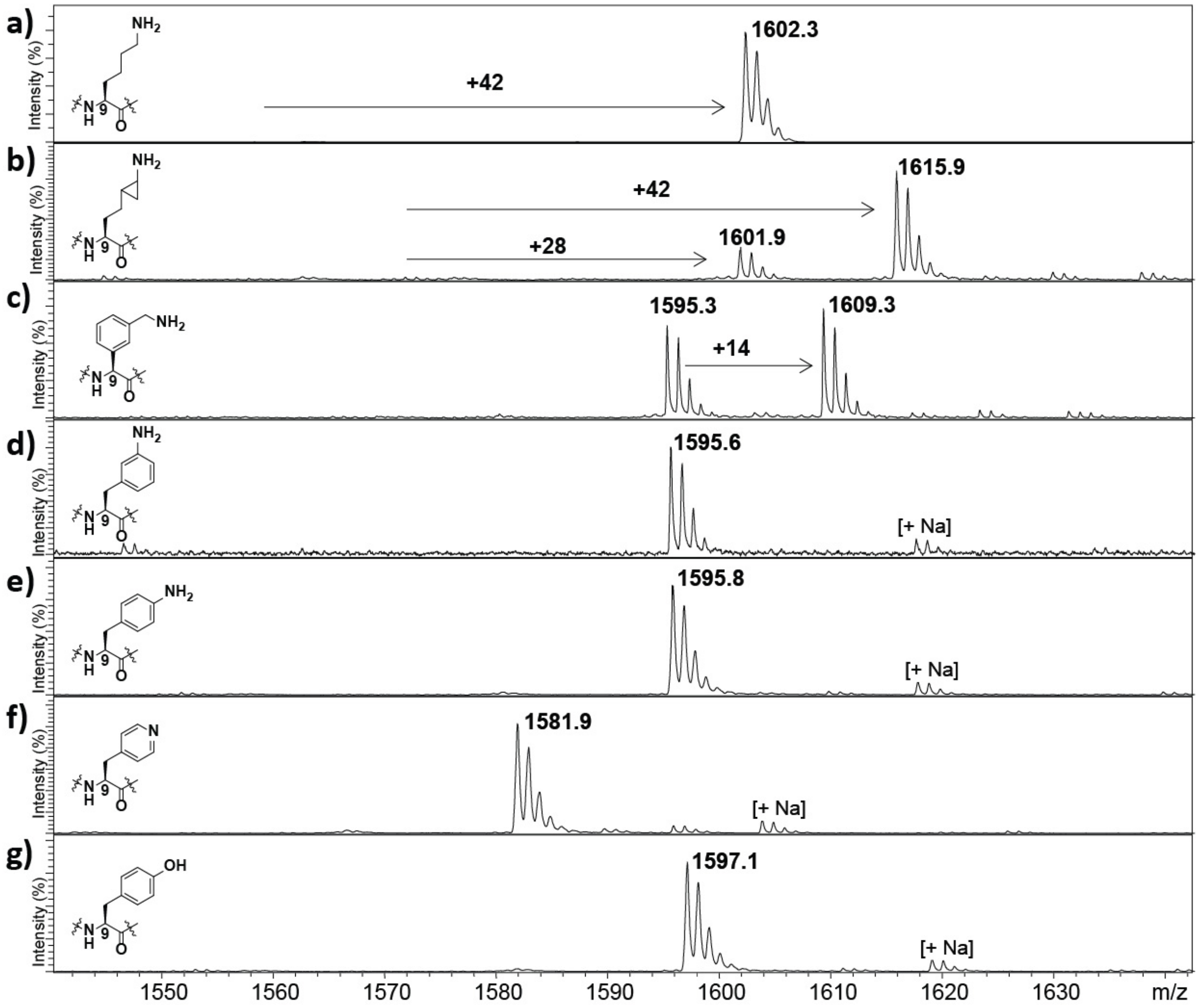

Figure S27. MALDI-MS spectra of methylation assay showing (**a**) major trimethylation of H3K9; (**b**) major trimethylation of H3K$_{CP}$9 and degree of dimethylation; (**c**) monomethylation of H3K$_{ba}$9; (**d**) a lack of methylation for H3F$_{3a}$9; (**e**) H3F$_{4a}$9; (**f**) H3A$_{p}$9; and (**g**) H3Y9 peptide (100 μM) in the presence of GLP (10 μM) and SAM (1 mM) after incubation for 5 hours at 37 °C.

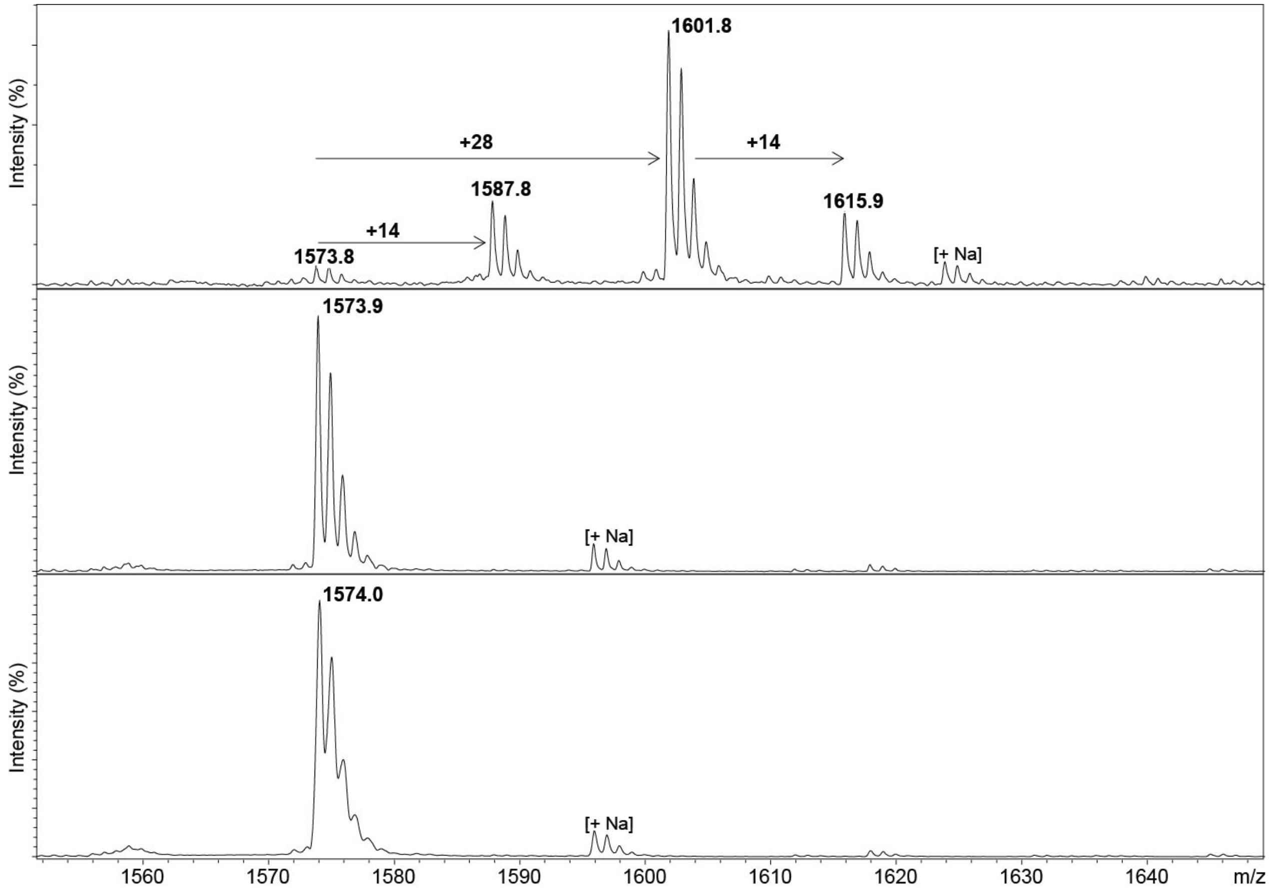

Figure S28. MALDI-MS spectra of methylation assay showing GLP (2 μM) catalyzed mono-, di-, and trimethylation of H3K_{CP}9 peptide (100 μM) in the presence of SAM (500 μM) after 1 hour at 37 °C (top panel). Control reaction in the absence of GLP (middle panel). Control reaction in the absence of SAM (bottom panel).

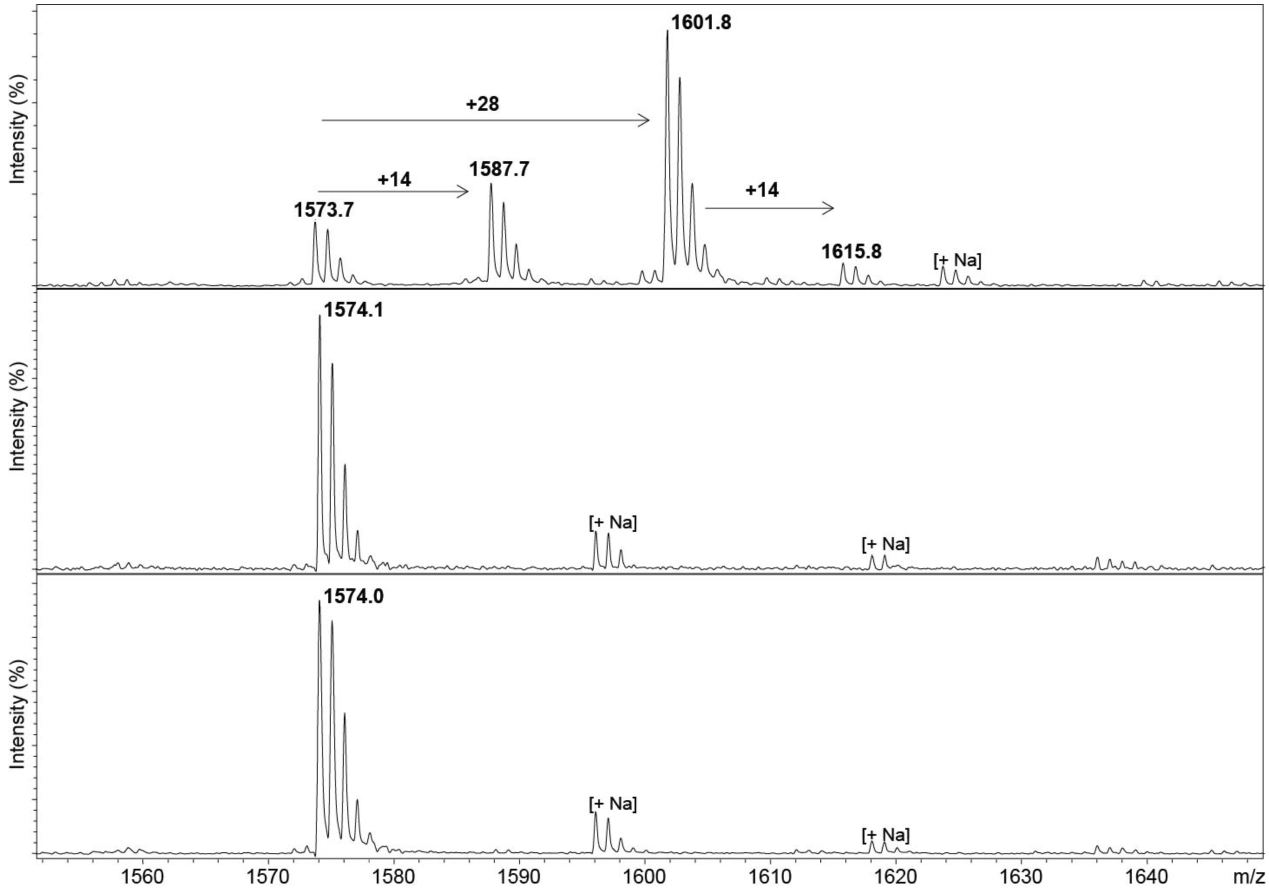

Figure S29. MALDI-MS spectra of methylation assay showing G9a (2 μM) catalyzed mono-, di-, and trimethylation of H3K$_{CP}$9 peptide (100 μM) in the presence of SAM (500 μM) after 1 hour at 37 °C (top panel). Control reaction in the absence of G9a (middle panel). Control reaction in the absence of SAM (bottom panel).

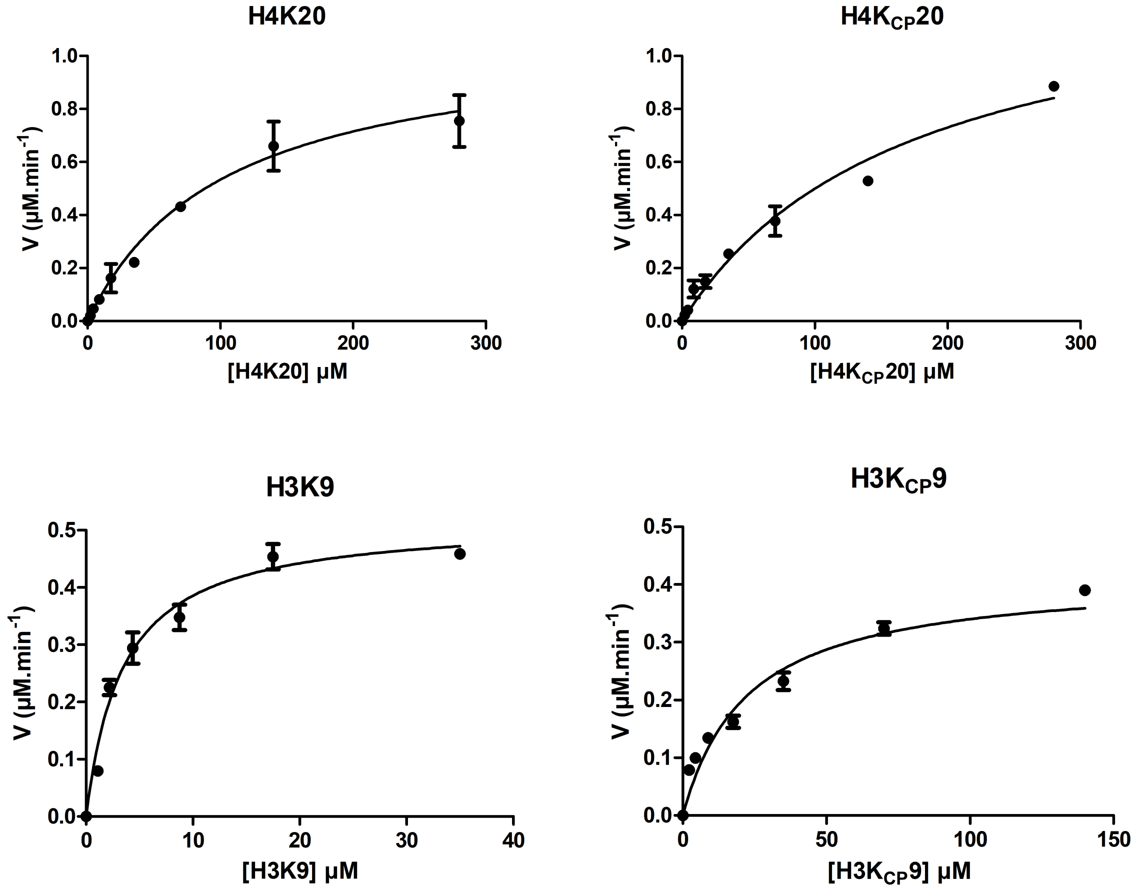

Figure S30. Kinetics plots for SETD8-catalyzed methylation of H4K20 and H4K$_{CP}$20 (top panel), and G9a-catalyzed methylation of H3K9 and H3K$_{CP}$9 (bottom panel). Data were obtained in duplicates.

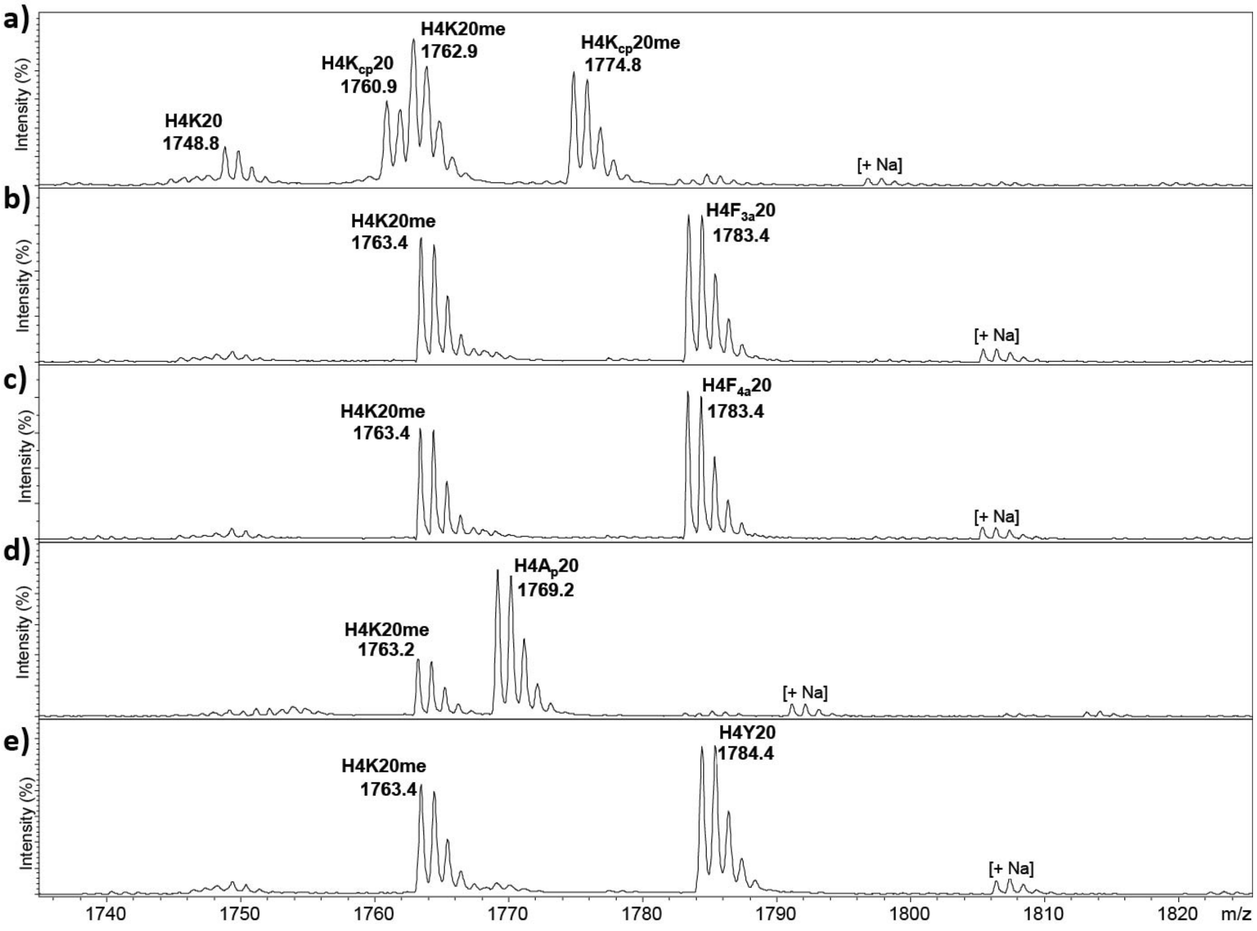

Figure S31. MALDI-MS based assay showing (**a**) SETD8-catalyzed monomethylation of H4K20 (100 μM) and H4K$_{CP}$20 (100 μM) in the presence of SAM (200 μM) after 1 h at 37 °C; (**b**) SETD8-catalyzed methylation of H4K20 (100 μM) in the presence of H4F$_{3a}$20 (100 μM) and SAM (200 μM) after 1 h at 37 °C; (**c**) SETD8-catalyzed methylation of H4K20 (100 μM) in the presence of H4F$_{4a}$20 (100 μM) and SAM (200 μM) after 1 h at 37 °C; (**d**) SETD8-catalyzed methylation of H4K20 (100 μM) in the presence of H4A$_p$20 (100 μM) and SAM (200 μM) after 1 h at 37 °C; (**e**) SETD8-catalyzed methylation of H4K20 (100 μM) in the presence of H4Y20 (100 μM) and SAM (200 μM) after 1 h at 37 °C.

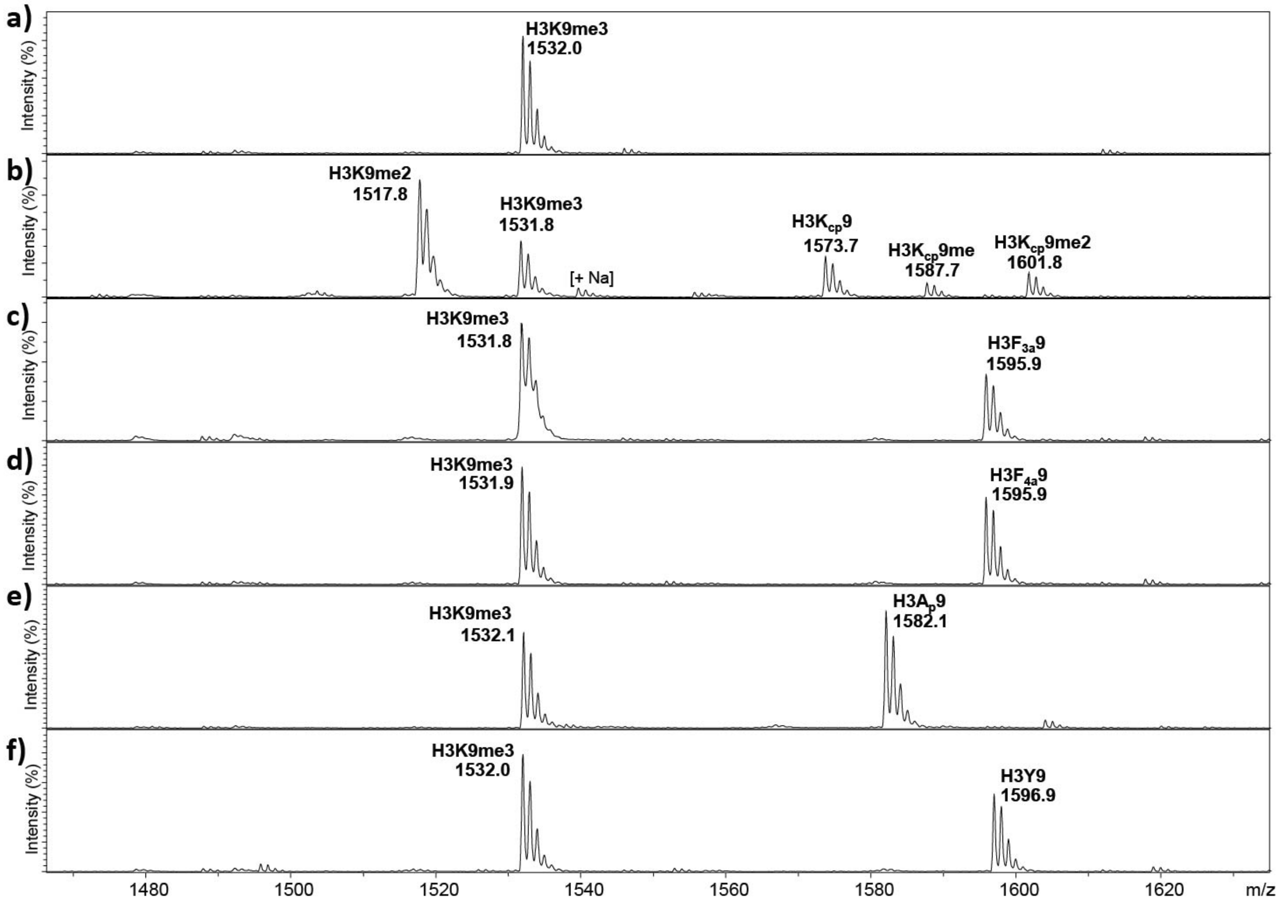

Figure S32. MALDI-MS based assay with (**a**) G9a-catalyzed trimethylation of $H3_{1\text{-}14}K9$ (100 μM) in the presence of SAM (500 μM) after 1 h at 37 °C; (**b**) G9a-catalyzed methylation of H3K9 (100 μM) and $H3K_{CP}9$ (100 μM) in the presence of SAM (200 μM) after 1 h at 37 °C; (**c**) G9a-catalyzed methylation of H3K9 (100 μM) in the presence of $H3F_{3a}9$ (100 μM) and SAM (200 μM) after 1 h at 37 °C; (**d**) G9a-catalyzed methylation of H3K9 (100 μM) in the presence of $H3F_{4a}9$ (100 μM) and SAM (200 μM) after 1 h at 37 °C; (**e**) G9a-catalyzed methylation of H3K9 (100 μM) in the presence of $H3A_p9$ (100 μM) and SAM (200 μM) after 1 h at 37 °C; (**f**) G9a-catalyzed methylation of H3K9 (100 μM) in the presence of H3Y9 (100 μM) and SAM (200 μM) after 1 h at 37 °C.

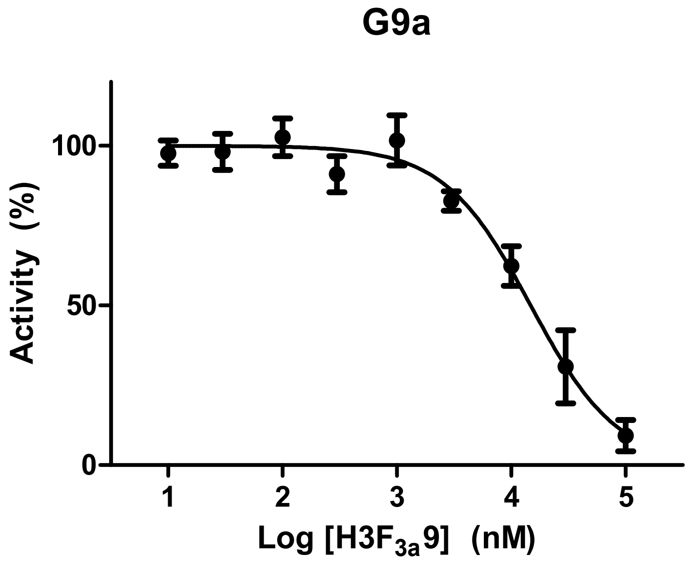

Figure S33. Inhibition curve (IC_{50} = 14.8 μM) for G9a (100 nM) in the presence of various concentrations (10 - 100000 nM) of $H3F_{3a}9$ peptide.

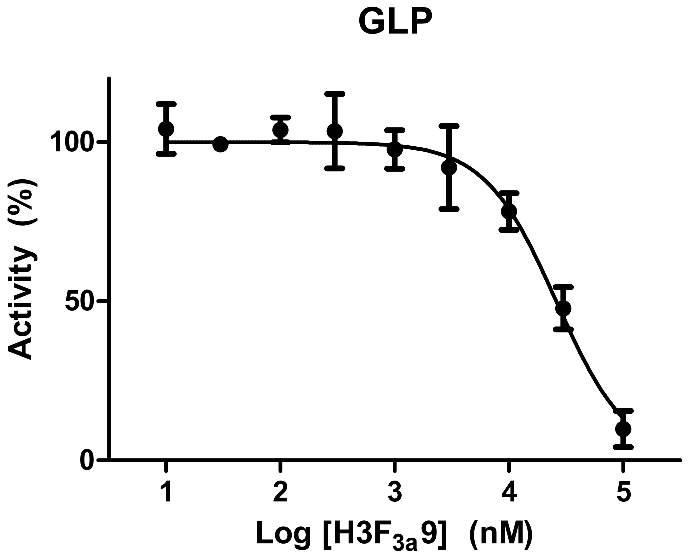

Figure S34. Inhibition curve (IC_{50} = 26.0 μM) for GLP (100 nM) in the presence of various concentrations (10 - 100000 nM) of $H3F_{3a}9$ peptide.

7. NMR supporting figures

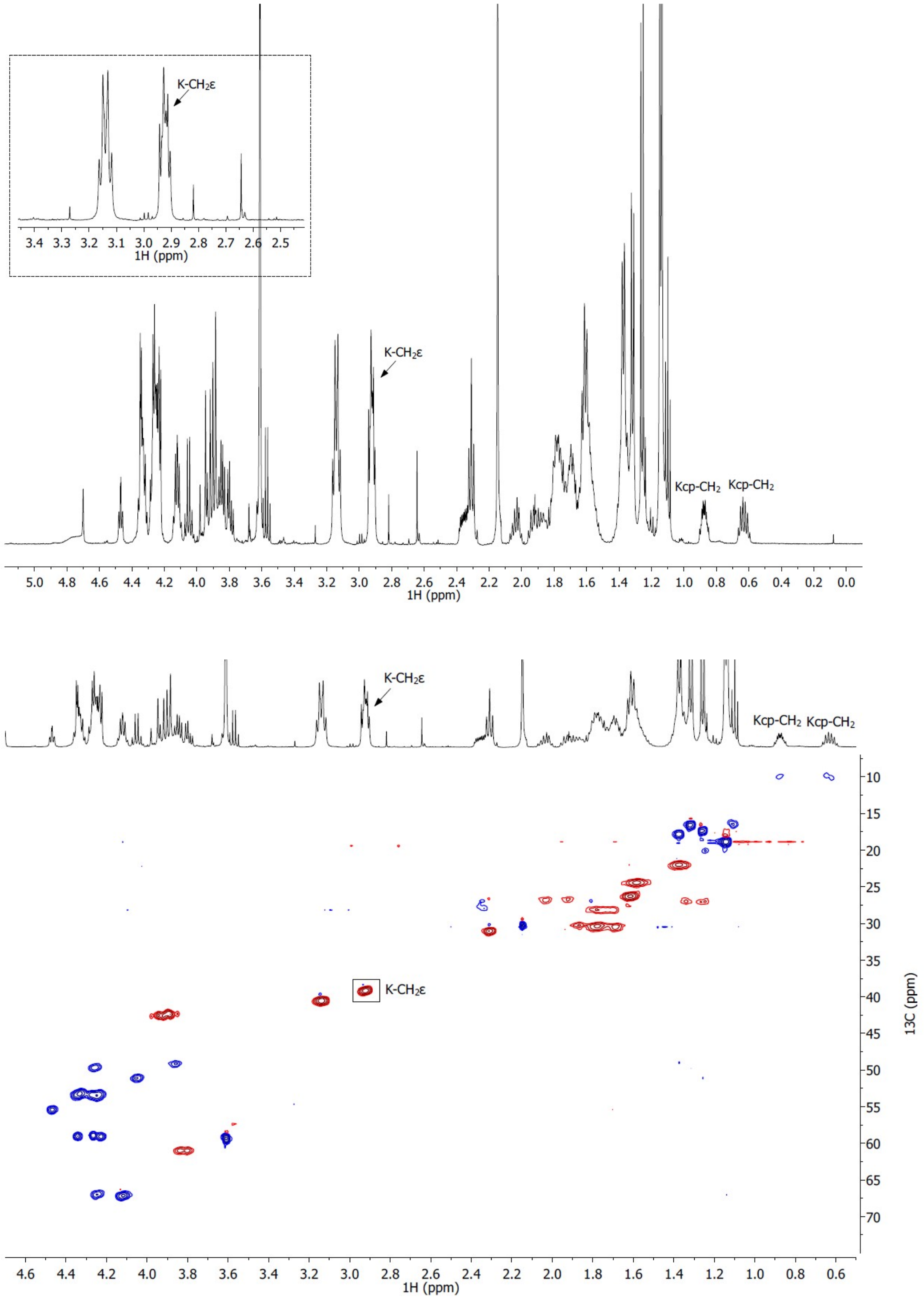

Figure S35. ^{1}H NMR spectrum of the H3K$_{CP}$9 peptide (top). Multiplicity-edited HSQC data of the H3K$_{cp}$9 peptide (bottom; blue = positive, CH/CH_3; red = negative, CH_2).

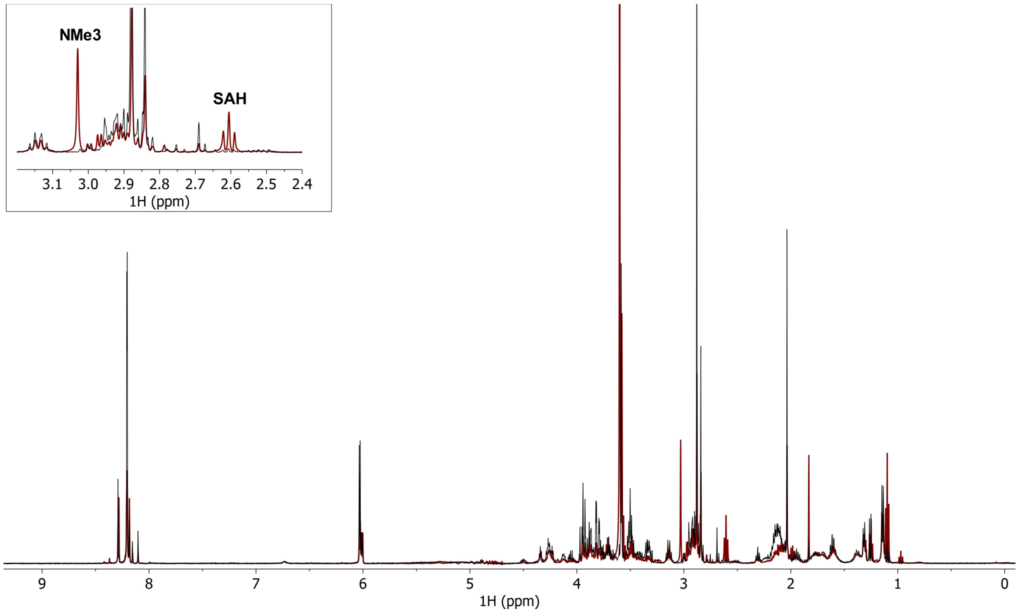

Figure S36. Stacked ^{1}H NMR spectra of GLP-catalyzed and nonenzymatic methylation of H3K9 peptide. Enzymatic reaction is in red and nonenzymatic reaction is in black.

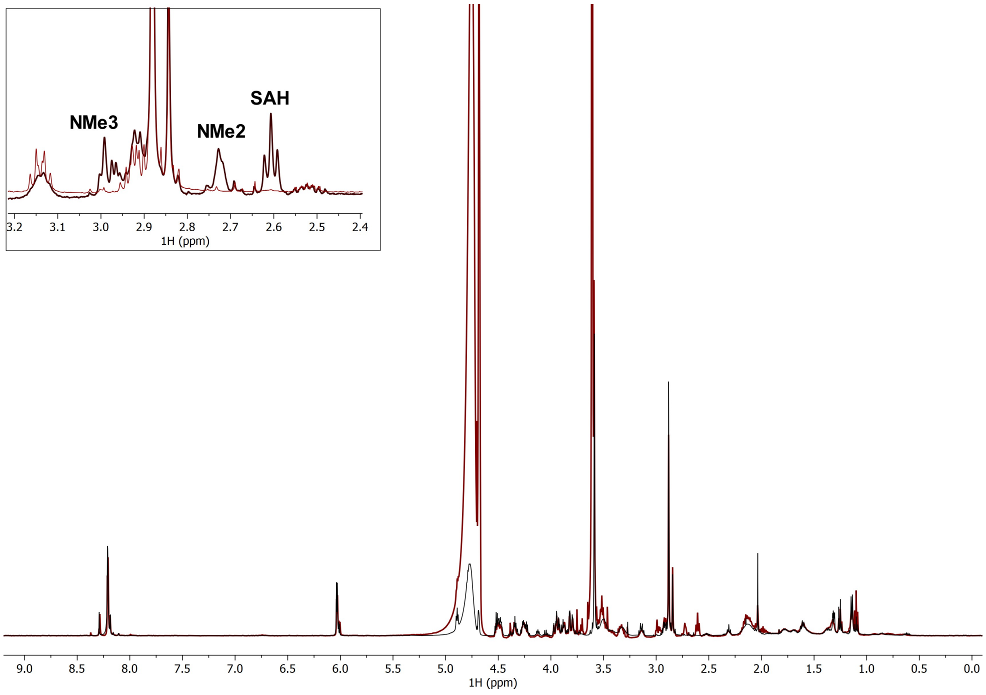

Figure S37. Stacked ^{1}H NMR spectra of GLP-catalyzed and nonenzymatic methylation of H3K$_{CP}$9 peptide. Enzymatic reaction is in black and nonenzymatic reaction is in red.

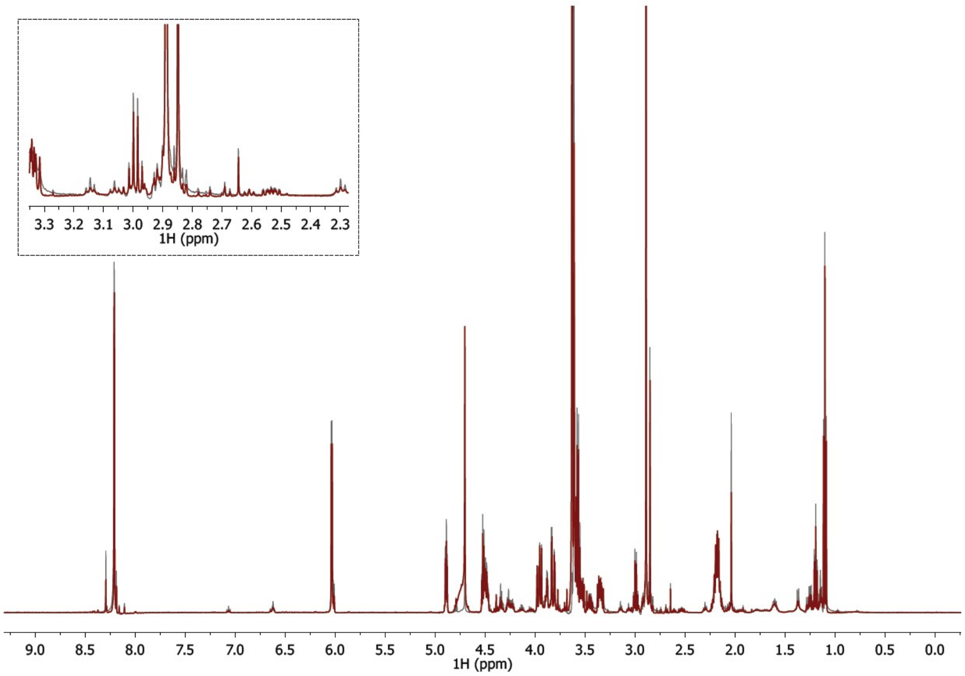

Figure S38. Stacked ^{1}H NMR spectra of GLP-catalyzed and nonenzymatic methylation of H3F$_{3a}$9 peptide. Enzymatic reaction is in red and nonenzymatic reaction is in black.

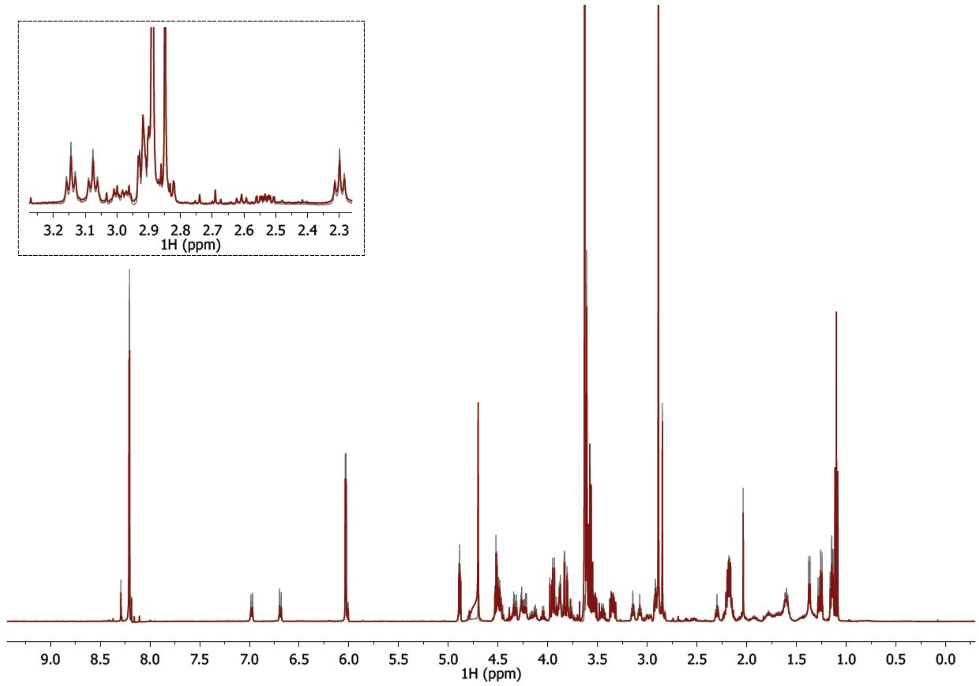

Figure S39. Stacked ^{1}H NMR spectra of GLP-catalyzed and nonenzymatic methylation of H3F$_{4a}$9 peptide. Enzymatic reaction is in red and nonenzymatic reaction is in black.

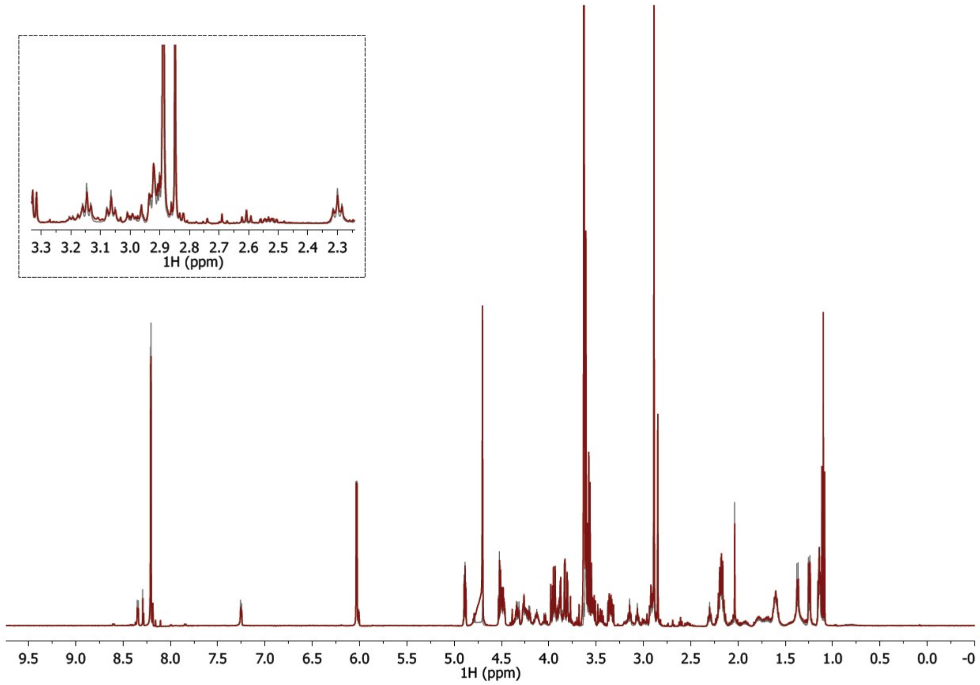

Figure S40. Stacked ^{1}H NMR spectra of GLP-catalyzed and nonenzymatic methylation of H3A$_p$9 peptide. Enzymatic reaction is in red and nonenzymatic reaction is in black.

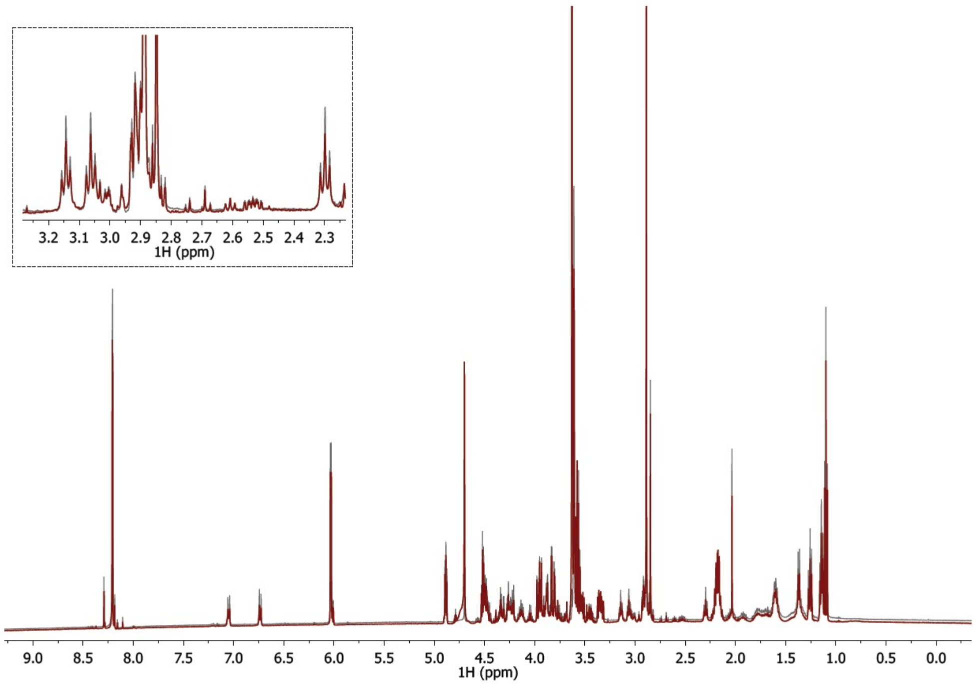

Figure S41. Stacked ^{1}H NMR spectra of GLP-catalyzed and nonenzymatic methylation of H3Y9 peptide. Enzymatic reaction is in red and nonenzymatic reaction is in black.

8. QM/MM studies

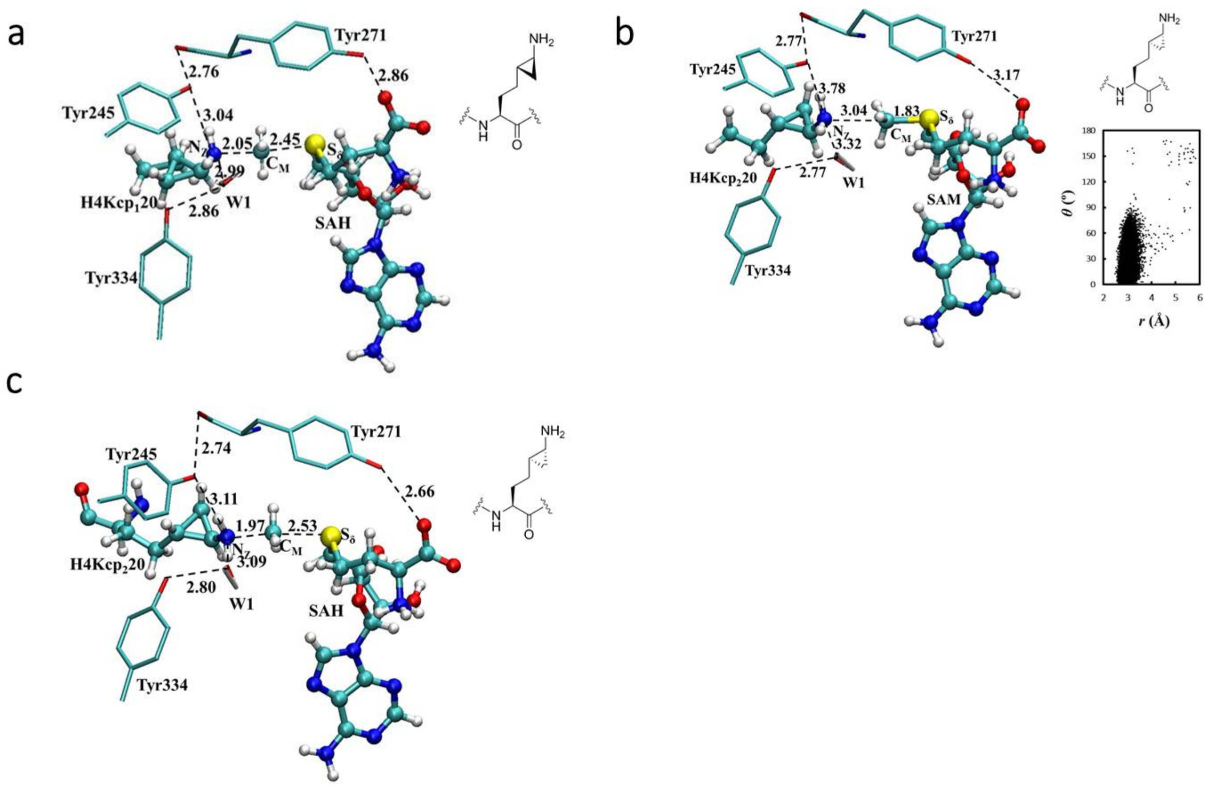

Figure S42. Representative active site structures of the complexes of SETD8 for the methylation reactions in Fig. 5a involving sterically demanding lysine analogs (other than those already given in Fig. 5b-5d). **(a)** Representative active site structure near transition state for the methylation involving one of two K_{cp} (see the chemical structure inserted next to the active site structure). SETD8 is shown in sticks, and SAM and lysine analog are in balls and sticks. Some average distances from the simulations are also given (in Å). **(b)** Representative active site structure of the reactant complex of SETD8 containing the other K_{cp}. The distribution map on the right shows the alignment of $N_\zeta H_2$ and the transferable methyl group in the reactant complex in terms of the distance (*r*) between N_ζ and C_M and the angle (θ) between the direction of electron lone pair on N_ζ and the C_M-S bond (the zero degree being the best alignment for the electron lone pair and the C_M-S bond). **(c)** The structure near transition state for the methylation reaction; the corresponding reactant complex is given in Fig. S43b.

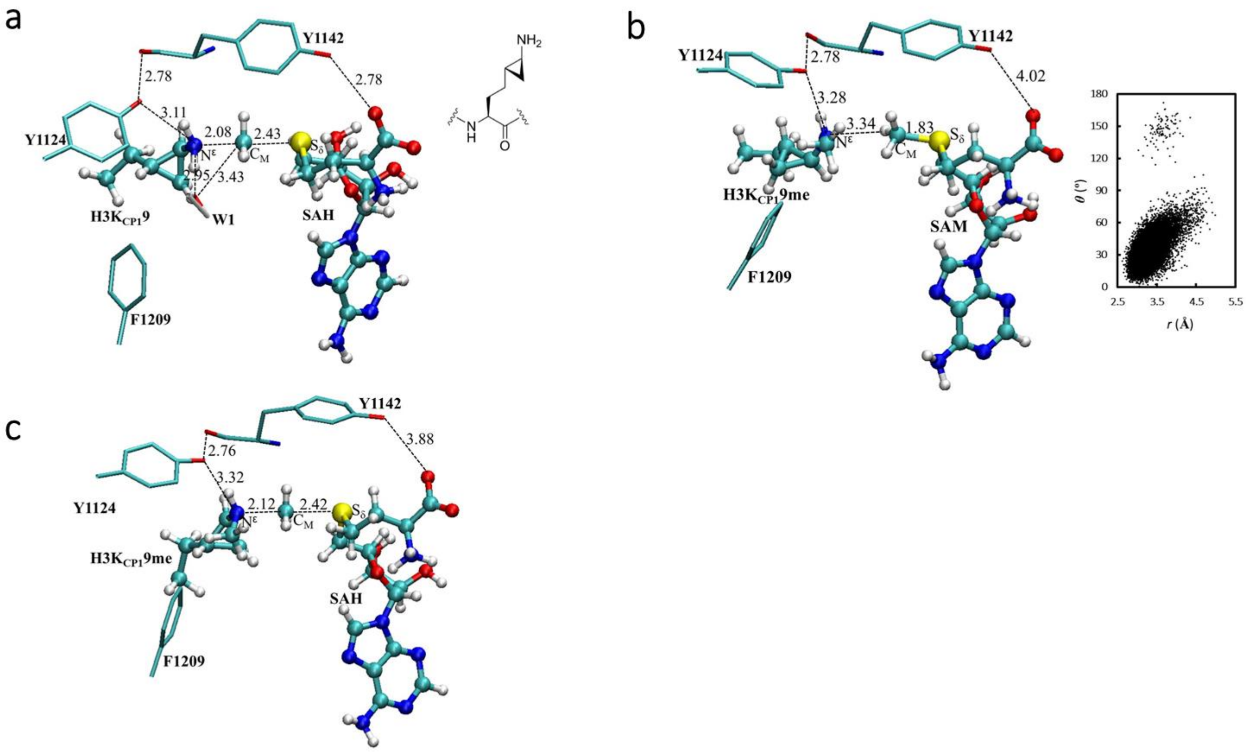

Figure S43. Representative active site structures of the complexes of GLP (other than those already given in Fig. 6b-6d) for the methylation reactions given in Fig. 6a involving one of two K_{cp} (see the chemical structure inserted in Fig. S44a). **(a)** Representative active site structure near transition state for monomethylation. **(b)** Representative active site structure of the reactant complex of GLP for di-methylation. **(c)** The structure near transition state for di-methylation; the corresponding reactant complex is given in Fig. S44b.

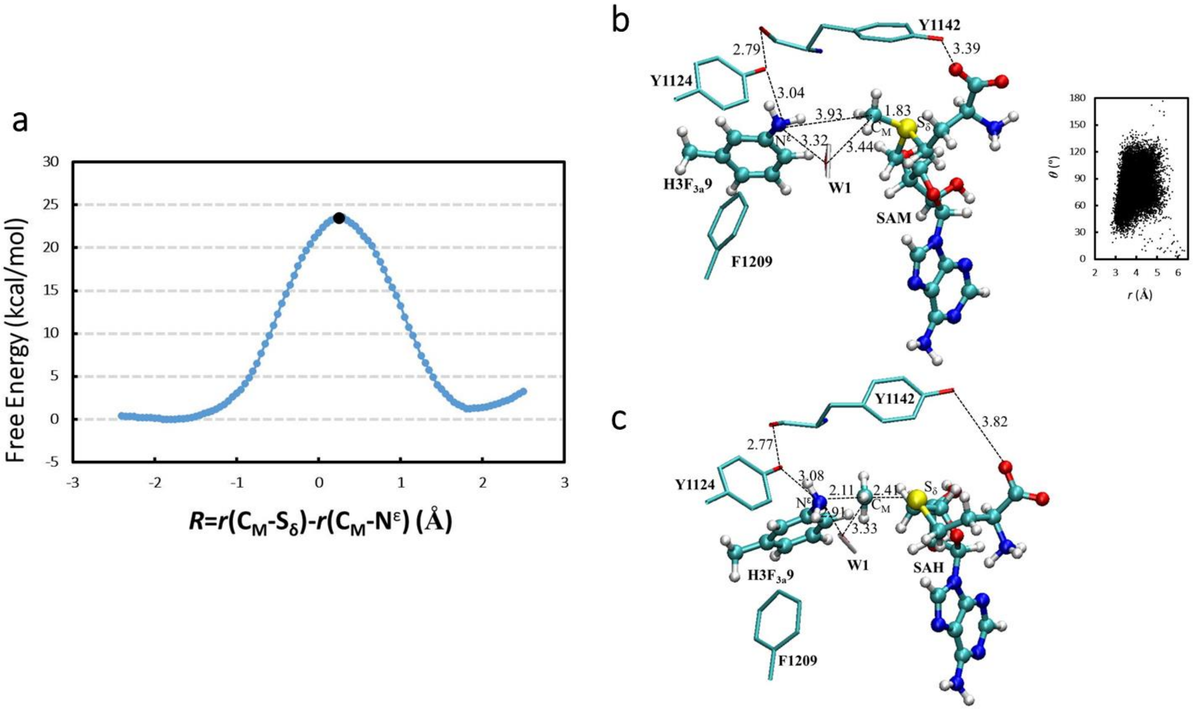

Figure S44. a) Free energy profile for the first methylation reaction in GLP involving F_{3a} with a free energy barrier of 23.6 kcal mol^{-1}. **b)** Representative active site structure of the reactant complex of GLP for the first methyl transfer to F_{3a} along with the distribution map. **c)** Representative active site structure of the near transition state for the methylation of F_{3a}.

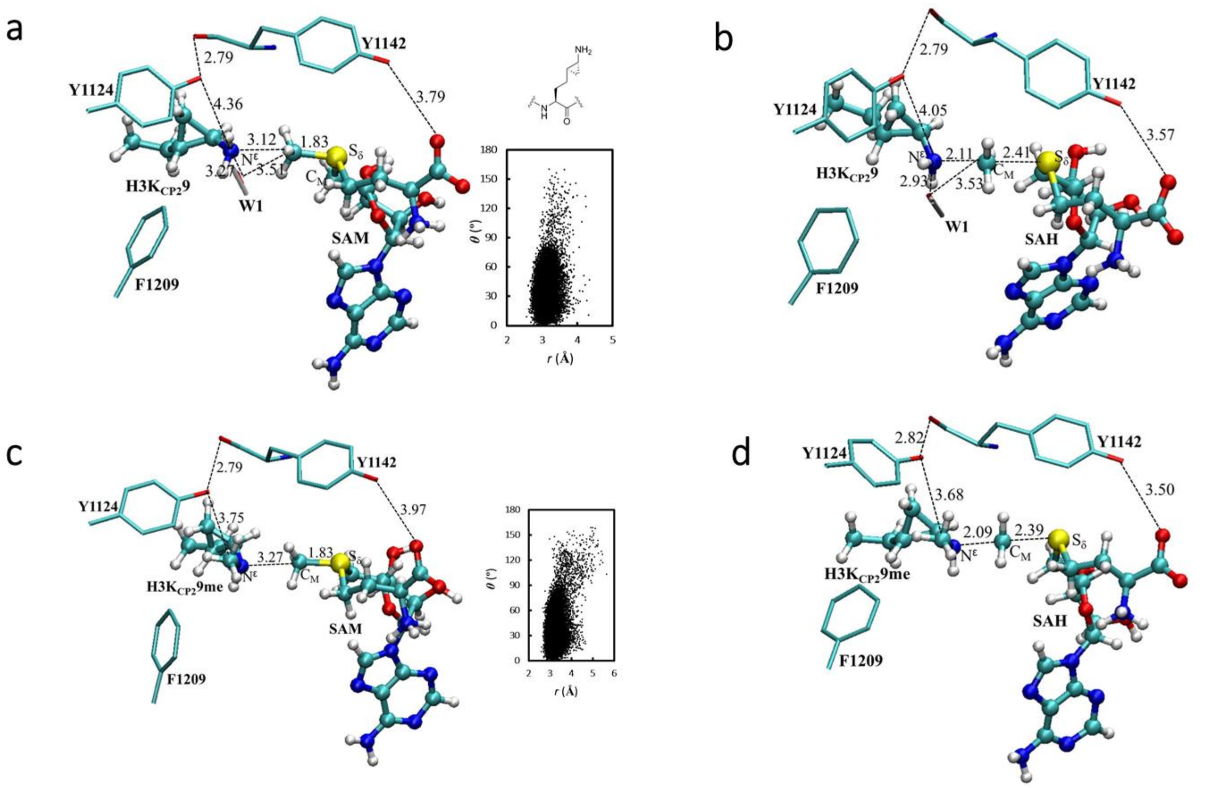

Figure S45. Representative active site structures of the complexes of GLP for the methylation reactions involving the other K_{cp} (see the chemical structure inserted in Fig. S46a). **(a)** Representative active site structure of the reactant complex of GLP for monomethylation along with the distribution map. **(b)** Representative active site structure near transition state for monomethylation. **(c)** Representative active site structure of the reactant complex of GLP for di-methylation. **(d)** The structure near transition state for di-methylation; the corresponding reactant complex is given in Fig. S46c.

9. References

1. Culhane, J. C., Wang, D., Yen, P. M., Cole, P. A. Comparative analysis of small molecules and histone substrate analogues as LSD1 lysine demethylase inhibitors. *J. Am. Chem. Soc.* 132, 3164–3176 (2010).

2. Patnaik, D., Chin, H. G., Pierre-Olivier Este`ve, Benner, J., Jacobsen, S. E., and Pradhan, S. Substrate specificity and kinetic mechanism of mammalian G9a histone H3 methyltransferase. *J. Biol. Chem.* 279, 53248–53258 (2004).
